# Supplementary material for: Cholesterol-stabilized membrane-active nanopores with anticancer activities
Source: Nat Commun. 2022 Oct 10;13:5985. doi: 10.1038/s41467-022-33639-5 (PMC9551035; doi:10.1038/s41467-022-33639-5)
Supplement: Supplementary file 1 — Supplementary Information [file 41467_2022_33639_MOESM1_ESM.pdf]

Supplementary Information

Cholesterol-Stabilized Membrane-Active Nanopores with Anticancer Activities

Jie Shen,[1,2] Yongting Gu,[1] Lingjie Ke,[1] Qiuping Zhang,[1] Yin Cao,[1] Yuchao Lin,[1] Zhen Wu,[1] Caisheng Wu,[1] Yuguang Mu,[3] Yun-Long Wu,[1] Changliang Ren,[1,4]* and Huaqiang Zeng[2]*

[1] Fujian Provincial Key Laboratory of Innovative Drug Target Research and State Key Laboratory of Cellular Stress Biology, School of Pharmaceutical Sciences, Xiamen University, Xiamen, Fujian 361102, China

[2] College of Chemistry, Fuzhou University, Fuzhou, Fujian 350116, China

[3] School of Biological Sciences, Nanyang Technological University, Singapore 637551

[4] Shenzhen Research Institute of Xiamen University, Shenzhen, Guangdong 518057, China

Corresponding emails: changliang.ren@xmu.edu.cn and hqzeng@fzu.edu.cn

1. **Supplementary Note 1**……………………………………………………………**S2**
2. **Supplementary Methods**…………………………………………………………**S3**
   - Synthetic Procedures and Compound Characterizations…………………………S3
   - SPQ Assay………………………………………………………………………S11
   - Cation Selectivity Study using HPTS Assay……………………………………S12
   - Dynamic Light Scattering Assay…………………………………………………S13
   - Molecular Dynamics Simulation …………………………………………………S14
   - In vitro Hemolytic Activity Assessment……………………………………………S15
3. **Supplementary Figures 1-42**………………………………………………………**S16**
4. **Supplementary ^{1}H NMR and ^{13}C NMR Spectra**…………………………………**S56**
5. **Supplementary References**…………………………………………………………**S78**

Supplementary Note 1

All the reagents were obtained from commercial suppliers and used as received unless otherwise noted. Aqueous solutions were prepared from MilliQ water. Flash column chromatography was performed using pre-coated 0.2 mm silica plates from Selecto Scientific. Chemical yields refer to pure isolated substances. ^{1}H NMR spectra were recorded on Bruker ACF-400 (400 MHz) using $CDCl_3$ or DMSO-d_6 as the solvent. $CDCl_3$ is referenced at $\delta = 7.26$ ppm and DMSO-d_6 at $\delta = 2.50$ ppm. Coupling constants (*J* values) are reported in Hertz (Hz). ^{1}H NMR data are recorded in the order: chemical shift value, multiplicity (s, singlet; d, doublet; t, triplet; q, quartet; m, multiplet; br, broad), number of protons. ^{13}C NMR spectra are proton-decoupled and recorded on Bruker ACF-400 (400 MHz) using $CDCl_3$ or DMSO-d_6 as the solvent. $CDCl_3$ is referenced at $\delta = 77$ ppm and DMSO-d_6 is referenced at 39.5 ppm. $CDCl_3$ (99.8%-Deuterated) and DMSO-d_6 (99.9%-Deuterated) were purchased from Sigma-Aldrich and used without further purification. Mass spectra were acquired with Waters 3100 mass spectrometer. Single channel current measurements in a planar lipid bilayer were carried out using Planar Lipid Bilayer Workstation (Warner Instruments, Hamden, CT). UV-Vis spectra were recorded on UV-Vis absorption spectrophotometer (UV1800, Shimadzu, Japan).

Supplementary Methods

Synthetic Procedures and Compound Characterizations

Ts-OEG-1

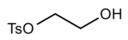

Ts-OEG-1

4-toluenesulfonyl chloride (3.81 g, 20.0 mmol) was dissolved in anhydrous ethylene glycol (30 mL) to which triethylamine (2.78 mL, 20.0 mmol) was added. The reaction was allowed to stir at room temperature for 14 h. The reaction mixture was then dissolved in dichloromethane (150 mL) and washed with water (3 x 100 mL). Romoval of dichloromethane solvent in vacuo gave the crude product, which was purified by flash column chromatography (methanol : dichloromethane = 1 : 50) to afford pure product **Ts-OEG-1** as a colorless liquid. Yield: 2.64 g, 61%. ^{1}H NMR (400 MHz, $CDCl_3$) δ 7.81 (d, *J* = 8.2 Hz, 2H), 7.36 (d, *J* = 8.0 Hz, 2H), 4.14 (dd, *J* = 6.2, 2.7 Hz, 2H), 3.82 (dd, *J* = 6.1, 2.8 Hz, 2H), 2.46 (s, 3H). ^{13}C NMR (100 MHz, $CDCl_3$) δ 145.18, 132.60, 130.01, 128.03, 71.65, 60.83, 21.72. MS-ESI: calculated for $[M+H]^+$ ($C_9H_{13}O_4S$)： *m/z* 217.05, found: *m/z* 217.17.

Ts-OEG-3

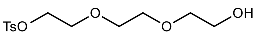

Ts-OEG-3

Triethylene glycol (3.00 g, 20.0 mmol) and 4-toluenesulfonyl chloride (3.81 g, 20.0 mmol) were dissolved in anhydrous dichloromethane (80 mL) to which triethylamine (2.78 mL, 20.0 mmol) was added. The reaction was allowed to stir at room temperature for 14 h. Romoval of the solvent in vacuo gave the crude product, which was purified by flash column chromatography (methanol : dichloromethane = 3 : 100) to afford pure product **Ts-OEG-3** as a colorless liquid. Yield: 2.19 g, 36%. ^{1}H NMR (400 MHz, $CDCl_3$) δ 7.85 – 7.76 (m, 2H), 7.34 (dd, *J* = 8.6, 0.6 Hz, 2H), 4.21 – 4.13 (m, 2H), 3.71 (ddd, *J* = 6.4, 5.3, 4.1 Hz, 4H), 3.62 – 3.55 (m, 6H), 2.45 (s, 3H). ^{13}C NMR (100 MHz, $CDCl_3$) δ 144.95, 132.86, 129.89, 128.02, 72.50, 70.81, 70.30, 69.18, 68.73, 61.78, 21.70. MS-ESI: calculated for $[M+H]^+$ ($C_{13}H_{21}O_6S$)： *m/z* 305.11, found: *m/z* 305.25.

Ts-OEG-5

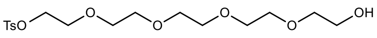

Ts-OEG-5

Pentaethylene glycol (2.38 g, 10.0 mmol) and 4-toluenesulfonyl chloride (1.91 g, 10.0 mmol) were dissolved in anhydrous dichloromethane (40 mL) to which triethylamine (1.39 mL, 10.0 mmol) was added. The reaction was allowed to stir at room temperature overnight. Romoval of the solvent in vacuo gave the crude product, which was purified by flash colunm (methanol : dichloromethane = 3 : 100) to afford pure product **Ts-OEG-3** as a colorless liquid. Yield: 1.18 g, 30%. ^{1}H NMR (400 MHz, $CDCl_3$) δ 7.73 (d, *J* = 8.4 Hz, 2H), 7.28 (d, *J* = 8.0 Hz, 2H), 4.09 (dd, *J* = 5.3, 4.3 Hz, 2H), 3.66 – 3.46 (m, 19H), 2.38 (s, 3H). ^{13}C NMR (100 MHz, $CDCl_3$) δ 144.85, 132.91, 129.86, 128.02, 72.62, 70.75, 70.58, 70.55, 70.51, 70.46, 70.24, 69.28, 68.70, 61.72, 21.70. MS-ESI: calculated for $[M+H]^+$ ($C_{17}H_{29}O_8S$)： *m/z* 393.16, found: *m/z* 393.39.

The synthesis of **Ts-OEG-7** follows the similar protocol as **Ts-OEG-5**

Ts-OEG-7

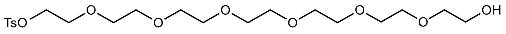

Ts-OEG-7

^{1}H NMR (400 MHz, $CDCl_3$) δ 7.80 (d, J = 8.3 Hz, 2H), 7.34 (d, J = 8.1 Hz, 2H), 4.19 – 4.10 (m, 2H), 3.74 – 3.58 (m, 26H), 2.45 (s, 3H). ^{13}C NMR (100 MHz, $CDCl_3$) δ 144.84, 132.93, 129.86, 128.03, 72.79, 70.72, 70.59, 70.54, 70.51, 70.48, 70.45, 70.16, 69.30, 68.66, 61.67, 21.69. MS-ESI: calculated for $[M+H]^+$ ($C_{21}H_{37}O_{10}S$)： *m/z* 481.21, found: *m/z* 481.36.

Dimethyl 5-hydroxyisophthalate

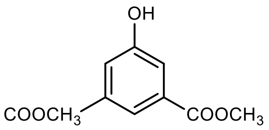

5-hydroxyisophthalic acid (3.64 g, 20.0 mmol) was dissolved in methanol (50 mL) to which concentrated H_2SO_4 (6.0 mL) was added. The mixture was heated under relux for 48 h. After removing most of organic solvent in vacuo, the wet residue was transferred into water (1.0 L) to yield a white precipitate, which was filtered and washed with water (3 x 300 mL) to afford pure product dimethyl 5-hydroxyisophthalate as a white solid. Yield: 3.84 g, 92%. ^{1}H NMR (400 MHz, DMSO-d_6) δ 10.32 (s, 1H), 7.92 (t, J = 1.6 Hz, 1H), 7.57 (d, J = 1.6 Hz, 2H), 3.89 (s, 6H). ^{13}C NMR (100 MHz, DMSO-d_6) δ 165.80 , 158.29 , 131.69 , 120.72 , 120.53 , 52.77 . MS-ESI: calculated for $[M+H]^+$ ($C_{10}H_{11}O_5$)： *m/z* 211.06, found: *m/z* 211.20.

Ch-H

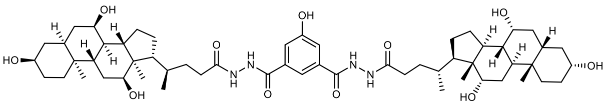

Ch-H

Dimethyl 5-hydroxyisophthalate (42 mg, 0.2 mmol) was dissolved in hot methanol (6 mL) to which hydrated hydrazine (0.2 mL) was added. The reaction mixture was heated under reflux for 8 h. Solvent was removed in vacuo to give the crude product 5-hydroxyisophthalohydrazide, which was directly used without further purification. 5-hydroxyisophthalohydrazide (0.4 mmol), cholic acid (327 mg, 0.8 mmol) and BOP (389 mg, 0.88 mmol) were dissolved in DMF (10.0 mL) to which DIEA (1.52 mL, 0.88 mmol) was added. The reaction was allowed to stir at room temperature for 14 h. After removing organic solvent in vacuo, acetonitrile (60 mL) was added to precipitate out the crude product, which was further purified by preparative HPLC to give pure product **Ch-H** as a white solid. Yield: 19.8 mg, 10.0%. ^{1}H NMR (400 MHz, DMSO-d_6) δ 10.29 (s, 2H), 10.14 (s, 1H), 9.88 (s, 2H), 7.79 (s, 1H), 7.41 (d, *J* = 1.3 Hz, 2H), 4.06 (s, 6H), 3.82 (s, 2H), 3.63 (s, 2H), 3.20 (dd, *J* = 8.2, 3.8 Hz, 2H), 2.23 – 1.96 (m, 12H), 1.74 (ddd, *J* = 29.8, 15.6, 9.5 Hz, 12H), 1.48 – 1.17 (m, 21H), 1.00 – 0.80 (m, 15H), 0.65 – 0.53 (m, 6H). ^{13}C NMR (100 MHz, DMSO-d_6) δ 172.42, 165.56, 157.84, 134.75, 117.83, 71.49, 70.90, 66.71, 46.62, 46.23, 41.98, 41.87, 35.78, 35.61, 35.36, 34.87, 31.97, 30.86, 29.03, 27.81, 26.68, 23.31, 23.12, 17.56, 12.87, 12.81. MS-ESI: calculated for $[M+H]^+$ ($C_{56}H_{87}O_{11}N_4$)： *m/z* 991.64, found: *m/z* 991.86.

Dimethyl 5-(2-hydroxyethoxy)isophthalate (a1)

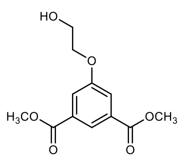

a1

A suspension of dimethyl 5-hydroxyisophthalate (312 mg, 2.00 mmol), **Ts-OEG-1** (649 mg, 3.00 mmol) and potassium carbonate (415 mg, 3.00 mmol) in DMF (20 mL) was heated at 85 °C for 12 h. Romoval of the solvent in vacuo gave the crude product, which was dissolved in dichloromethane (50 mL), washed with water (3 x 50 mL) and purified by flash column chromatography (methanol : dichloromethane = 1 : 50) to afford pure product **a1** as a colorless liquid. Yield: 356 mg, 70%. ^{1}H NMR (400 MHz, $CDCl_3$) δ 8.25 (t, *J* = 1.4 Hz, 1H), 7.73 (d, *J* = 1.4 Hz, 2H), 4.17 – 4.12 (m, 2H), 3.99 – 3.96 (m, 2H), 3.91 (s, 6H). ^{13}C NMR (100 MHz, $CDCl_3$) δ 166.09, 158.76, 131.77, 123.27, 119.85, 69.86, 61.13, 52.50. MS-ESI: calculated for $[M+H]^+$ ($C_{12}H_{15}O_6$)： *m/z* 255.09, found: *m/z* 255.21.

Dimethyl 5-(2-(2-(2-hydroxyethoxy)ethoxy)ethoxy)isophthalate (a3)

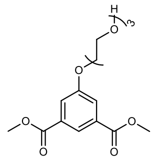

a3

^{1}H NMR (400 MHz, $CDCl_3$) δ 8.31 (t, *J* = 1.4 Hz, 1H), 7.80 (d, *J* = 1.4 Hz, 2H), 4.25 (dd, *J* = 5.3, 4.0 Hz, 2H), 3.96 – 3.89 (m, 8H), 3.77 – 3.72 (m, 6H), 3.66 - 3.62 (m, 2H). ^{13}C NMR (100 MHz, $CDCl_3$) δ 166.15, 158.81, 131.76, 123.25, 119.98, 72.52, 70.93, 70.38, 69.55, 68.01, 61.78, 52.49. MS-ESI: calculated for $[M+H]^+$ ($C_{16}H_{23}O_8$)： *m/z* 343.14, found: *m/z* 343.26.

Ch-P1

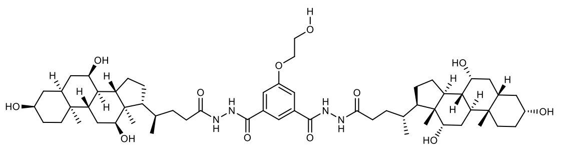

Ch-P1

a1 (127 mg, 0.50 mmol) was dissolved in hot methanol (15 mL) to which hydrated hydrazine (0.50 mL) was added. The reaction mixture was heated under reflux for 8 h. Solvent was removed in vacuo to give the crude product **b1**, which was directly used without further purification. **b1** (0.50 mmol), cholic acid (409 mg, 1.00 mmol) and BOP (487 mg, 1.1 mmol) were dissolved in DMF (10 mL) to which DIEA (0.38 mL, 2.2 mmol) was added. The reaction was allowed to stir at room temperature for 14 h. After removing organic solvent in *vacuo*, acetonitrile (100 mL) was added to precipitate out the crude product, which was further purified by preparative HPLC to give pure product **Ch-P1** as a white solid. Yield: 42 mg, 8.1%. ^{1}H NMR (400 MHz, DMSO-*d6*) δ 10.41 (s, 2H), 9.92 (s, 2H), 7.96 (s, 1H), 7.59 (d, *J* = 1.2 Hz, 2H), 4.11 (t, *J* = 4.8 Hz, 2H), 3.81 (s, 2H), 3.78 – 3.75 (m, 2H), 3.62 (s, 4H), 3.48 (s, 4H), 3.23 – 3.15 (m, 2H), 2.24 – 1.95 (m, 10H), 1.83 – 1.62 (m, 12H), 1.46 – 1.15 (m, 24H), 0.99 - 0.93 (m, 7H), 0.86 – 0.80 (m, 8H), 0.62 (s, 6H). ^{13}C NMR (100 MHz, DMSO-*d6*) δ 172.45, 165.12, 159.04, 134.62, 119.70, 116.84, 71.48, 70.90, 66.70, 59.89, 46.62, 46.23, 41.97, 41.86, 35.78, 35.61, 35.35, 34.86, 31.98, 30.86, 29.03, 27.80, 26.67, 23.30, 23.11, 17.55, 12.87. MS-ESI: calculated for $[M+H]^+$ ($C_{58}H_{91}O_{12}N_4$)： *m/z* 1035.66, found: *m/z* 1035.89.

The synthesis of **Ch-P3** and **Ch-P7** follows the similar protocol as **Ch-P1**

Ch-P3

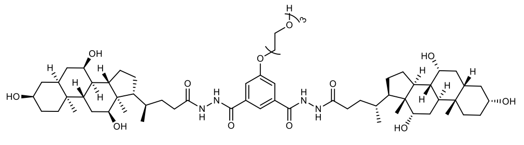

Ch-P3

^{1}H NMR (400 MHz, DMSO-d_6) δ 10.40 (s, 2H), 9.92 (s, 2H), 7.97 (s, 1H), 7.60 (d, J = 1.1 Hz, 2H), 4.23 (d, J = 3.5 Hz, 2H), 3.81 (s, 4H), 3.62 (dd, J = 5.5, 3.0 Hz, 4H), 3.57 – 3.54 (m, 4H), 3.48 (s, 4H), 3.43 (s, 4H), 3.19 (dd, J = 8.1, 3.7 Hz, 2H), 2.25 – 1.96 (m, 10H), 1.83 – 1.63 (m, 12H), 1.50 – 1.16 (m, 24H), 0.98 (d, J = 6.4 Hz, 7H), 0.87 – 0.77 (m, 8H), 0.60 (s, 6H). ^{13}C NMR (100 MHz, DMSO-d_6) δ 172.45, 165.08, 158.78, 134.64, 119.85, 116.78, 72.84, 71.48, 70.90, 70.44, 70.27, 69.23, 68.18, 66.71, 60.66, 46.62, 46.23, 41.98, 41.87, 35.78, 35.61, 35.36, 34.87, 31.98, 30.86, 29.03, 27.80, 26.68, 23.31, 23.12, 17.55, 12.87. MS-ESI: calculated for $[M+H]^+$ ($C_{62}H_{99}O_{14}N_4$)： *m/z* 1123.72, found: *m/z* 1123.91.

Ch-P7

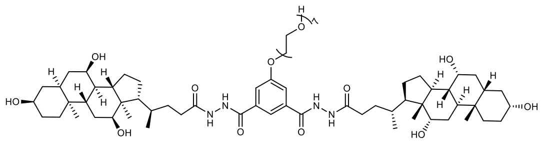

Ch-P7

^{1}H NMR (400 MHz, DMSO-d_6) δ 10.40 (s, 2H), 9.92 (s, 2H), 7.97 (s, 1H), 7.60 (d, J = 1.1 Hz, 2H), 4.36 (d, J = 4.3 Hz, 2H), 4.22 (s, 2H), 4.16 (d, J = 3.4 Hz, 2H), 4.05 (d, J = 3.3 Hz, 2H), 3.80 (d, J = 2.9 Hz, 4H), 3.62 (dd, J = 5.7, 3.1 Hz, 4H), 3.57 – 3.44 (m, 20H), 3.40 (dd, J = 7.8, 3.0 Hz, 2H), 3.18 (dd, J = 8.7, 6.1 Hz, 2H), 2.25 – 1.97 (m, 10H), 1.85 – 1.59 (m, 12H), 1.54 – 1.10 (m, 24H), 0.97 (s, 7H), 0.86 – 0.79 (m, 8H), 0.61 (s, 6H). ^{13}C NMR (100 MHz, DMSO-d_6) δ 170.29, 162.91, 156.64, 132.48, 117.70, 114.64, 70.66, 69.33, 68.75, 68.26, 68.12, 68.09, 67.07, 66.03, 64.56, 58.51, 44.48, 44.08, 39.83, 39.71, 33.63, 33.46, 33.21, 32.72, 29.83, 28.71, 26.88, 25.66, 24.53, 21.16, 20.97, 15.40, 10.72. MS-ESI: calculated for $[M+H]^+$ ($C_{70}H_{115}O_{18}N_4$)： *m/z* 1299.82, found: *m/z* 1300.44.

Ch-P5

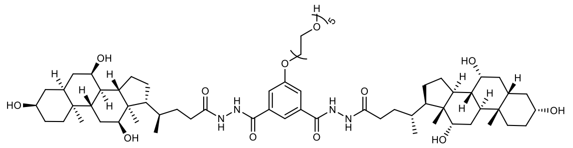

Ch-P5

Ch-H (198 mg, 0.2 mmol) and **Ts-OEG-5** (174 mg, 0.4 mmol) were dissolved in DMF (10 mL) to which K_2CO_3 (56 mg, 0.4 mmol) was added. The mixture was heated at 85 ºC for 24 h. The reaction mixture was then filtered and the solvent was removed in vacuo. The reaction mixture was re-dissolved in methanol (15 mL), to which trifluoroacetic acid (0.3 mL) was added. Removal of the solvent afforded the crude product which was further purified by preparative HPLC to give pure product **Ch-P5** as a white solid. Yield: 44 mg, 18%. ^{1}H NMR (400 MHz, DMSO-d_6) δ 10.40 (s, 2H), 9.92 (s, 2H), 7.97 (s, 1H), 7.60 (d, J = 1.1 Hz, 2H), 4.23 (s, 2H), 3.81 (s, 4H), 3.63 – 3.61 (m, 4H), 3.58 – 3.55 (m, 3H), 3.51 (d, J = 4.4 Hz, 9H), 3.48 (d, J = 5.1 Hz, 4H), 3.43 – 3.40 (m, 4H), 3.22 – 3.16 (m, 2H), 2.27 – 1.97 (m, 12H), 1.75 (ddd, J = 39.3, 25.8, 10.8 Hz, 12H), 1.50 – 1.15 (m, 22H), 0.96 (t, J = 10.4 Hz, 7H), 0.89 – 0.76 (m, 8H), 0.65 – 0.52 (m, 6H). ^{13}C NMR (100 MHz, DMSO-d_6) δ 170.30, 162.92, 156.64, 132.48,

117.70, 114.64, 70.65, 69.33, 68.75, 68.26, 68.13, 68.10, 68.08, 67.07, 66.03, 64.56, 58.51, 44.48, 44.09, 39.83, 39.72, 33.46, 33.21, 32.72, 28.71, 26.88, 25.66, 24.53, 21.16, 20.97, 15.40, 10.72. MS-ESI: calculated for $[M+H]^+$ ($C_{66}H_{107}O_{16}N_4$): *m/z* 1211.77, found: *m/z* 1212.02.

Dimethyl 5-methoxyisophthalate (c1)

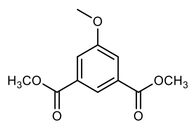

c1

A suspension of dimethyl 5-hydroxyisophthalate (420 mg, 2.00 mmol), iodomethane (0.25 mL, 4.00 mmol), and potassium carbonate (415 mg, 3.00 mmol) in DMF (20 mL) was heated at 85 °C for 12 h. Romoval of the solvent in vacuo gave the crude product, which was dissolved in dichloromethane (50 mL), washed with water (3 x 50 mL) and purified by flash column chromatography (ethyl acetate : n-hexane = 1 : 8) to afford pure product **c1** as a white solid. Yield: 381 mg, 85%. ^{1}H NMR (400 MHz, $CDCl_3$) δ 8.26 (t, *J* = 1.4 Hz, 1H), 7.74 (d, *J* = 1.3 Hz, 2H), 3.93 (s, 6H), 3.88 (s, 3H). ^{13}C NMR (100 MHz, $CDCl_3$) δ 166.19, 159.67, 131.75, 122.96, 119.29, 55.81, 52.47. MS-ESI: calculated for $[M+H]^+$ ($C_{11}H_{13}O_5$): *m/z* 225.08, found: *m/z* 225.22.

Dimethyl 5-ethoxyisophthalate (c2)

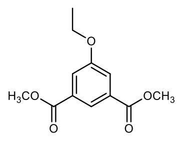

c2

A suspension of dimethyl 5-hydroxyisophthalate (420 mg, 2.00 mmol), bromoethane (0.22 mL, 3.00 mmol) and potassium carbonate (415 mg, 3.00 mmol) in DMF (20 mL) was heated at 85 °C for 12 h. Romoval of the solvent in vacuo gave the crude product, which was dissolved in dichloromethane (50 mL), washed with water (3 x 50 mL) and purified by flash column chromatography (ethyl acetate : n-hexane = 1 : 8) to afford pure product **c2** as a white solid. Yield: 367 mg, 77%. ^{1}H NMR (400 MHz, $CDCl_3$) δ 8.25 (t, *J* = 1.4 Hz, 1H), 7.73 (d, *J* = 1.4 Hz, 2H), 4.11 (q, *J* = 7.0 Hz, 2H), 3.93 (s, 6H), 1.44 (t, *J* = 7.0 Hz, 3H). ^{13}C NMR (100 MHz, $CDCl_3$) δ 166.25, 159.03, 131.69, 122.81, 119.82, 64.14, 52.44, 14.69. MS-ESI: calculated for $[M+H]^+$ ($C_{12}H_{15}O_5$): *m/z* 239.09, found: *m/z* 239.17.

The synthesis of **c4, c6** and **c8** follow the similar protocol as **c2**

Dimethyl 5-butoxyisophthalate (c4)

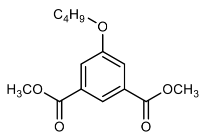

c4

^{1}H NMR (400 MHz, $CDCl_3$) δ 8.25 (t, *J* = 1.5 Hz, 1H), 7.74 (d, *J* = 1.5 Hz, 2H), 4.04 (t, *J* = 6.5 Hz, 2H), 3.93 (s, 6H), 1.83 – 1.73 (m, 2H), 1.55 – 1.43 (m, 2H), 0.98 (t, *J* = 7.4 Hz, 3H). ^{13}C NMR (100 MHz, $CDCl_3$) δ 166.28, 159.25, 131.68, 122.76, 119.84, 68.31, 52.45, 31.14, 19.20, 13.85. MS-ESI: calculated for $[M+H]^+$ ($C_{14}H_{19}O_5$): *m/z* 267.12, found: *m/z* 267.28.

Dimethyl 5-(hexyloxy)isophthalate (c6)

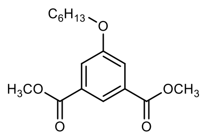

c6

^{1}H NMR (400 MHz, $CDCl_3$) δ 8.25 (t, *J* = 1.4 Hz, 1H), 7.74 (d, *J* = 1.4 Hz, 2H), 4.03 (t, *J* = 6.5 Hz, 2H), 3.93 (s, 6H), 1.80 (dq, *J* = 13.0, 6.5 Hz, 2H), 1.46 (dd, *J* = 10.3, 4.7 Hz, 2H), 1.34 (dq, *J* = 7.0, 3.5 Hz, 4H), 0.91 (t, *J* = 7.4 Hz, 3H). ^{13}C NMR (100 MHz, $CDCl_3$) δ 166.30, 159.24, 131.65, 122.75, 119.83, 68.62, 52.48, 31.55, 29.07, 25.68, 22.64, 14.09. MS-ESI: calculated for $[M+H]^+$ ($C_{16}H_{23}O_5$)： *m/z* 295.15, found: *m/z* 295.36.

Dimethyl 5-(octyloxy)isophthalate (c8)

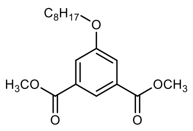

c8

^{1}H NMR (400 MHz, $CDCl_3$) δ 8.25 (t, *J* = 1.4 Hz, 1H), 7.74 (d, *J* = 1.5 Hz, 2H), 4.03 (t, *J* = 6.5 Hz, 2H), 3.93 (s, 6H), 1.80 (dq, *J* = 13.1, 6.6 Hz, 2H), 1.52 – 1.40 (m, 2H), 1.35 – 1.25 (m, 8H), 0.89 (t, *J* = 7.4 Hz, 3H). ^{13}C NMR (100 MHz, $CDCl_3$) δ 166.30, 159.24, 131.65, 122.75, 119.84, 68.62, 52.48, 31.84, 29.34, 29.27, 29.11, 26.00, 22.71, 14.17. MS-ESI: calculated for $[M+H]^+$ ($C_{18}H_{27}O_5$)： *m/z* 323.19, found: *m/z* 323.36.

Ch-C1

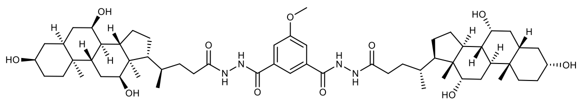

Ch-C1

Dimethyl 5-methoxyisophthalate (**c1**, 90 mg, 0.40 mmol) was dissolved in hot methanol (15 mL) to which hydrated hydrazine (0.40 mL) was added. The reaction mixture was heated under reflux for 8 h. Solvent was removed in vacuo to give the crude product **d1**, which was directly used without further purification. **d1** (0.40 mmol), cholic acid (327 mg, 0.80 mmol) and BOP (389 mg, 0.88 mmol) were dissolved in DMF (10 mL) to which DIEA (0.31 mL, 1.80 mmol) was added. The reaction was allowed to stir at room temperature for 14 h. After removing organic solvent in vacuo, acetonitrile (100 mL) was added to precipitate out the crude product, which was further purified by preparative HPLC to give pure product **Ch-C1** as a white solid. Yield: 26 mg, 6.2%. ^{1}H NMR (400 MHz, DMSO-d_6) δ 10.41 (s, 2H), 9.92 (s, 2H), 7.97 (s, 1H), 7.59 (d, *J* = 1.3 Hz, 2H), 4.36 (d, *J* = 4.3 Hz, 2H), 4.16 (d, *J* = 3.4 Hz, 2H), 4.05 (d, *J* = 3.3 Hz, 2H), 3.88 (s, 3H), 3.81 (d, *J* = 2.7 Hz, 2H), 3.63 (s, 2H), 3.19 (dd, *J* = 15.2, 10.7 Hz, 2H), 2.28 – 1.94 (m, 10H), 1.86 – 1.59 (m, 12H), 1.52 – 1.17 (m, 23H), 0.98 (d, *J* = 6.4 Hz, 7H), 0.86 – 0.76 (m, 8H), 0.62 (s, 6H). ^{13}C NMR (100 MHz, DMSO-d_6) δ 172.46, 165.11, 159.58, 134.64, 119.70, 116.28, 71.48, 70.90, 66.70, 56.18, 46.62, 46.23, 41.97, 41.86, 35.78, 35.61, 35.35, 34.86, 31.98, 30.86, 29.03, 27.80, 26.67, 23.31, 23.11, 17.55, 12.87. MS-ESI: calculated for $[M+H]^+$ ($C_{57}H_{89}O_{11}N_4$)： *m/z* 1005.65, found: *m/z* 1005.85.

The synthesis of **Ch-C2**, **Ch-C4**, **Ch-C6** and **Ch-C8** follows the similar protocol as **Ch-C1**

Ch-C2

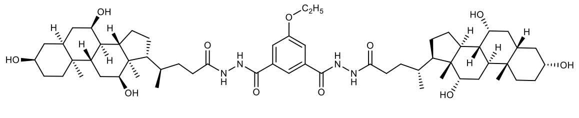

Ch-C2

^{1}H NMR (400 MHz, DMSO-*d*$_6$) δ 10.40 (s, 2H), 9.92 (s, 2H), 7.96 (s, 1H), 7.57 (d, *J* = 1.2 Hz, 2H), 4.18 – 4.12 (m, 2H), 3.81 (s, 4H), 3.61 (t, *J* = 9.4 Hz, 8H), 2.29 – 1.97 (m, 10H), 1.86 – 1.62 (m, 12H), 1.46 – 1.19 (m, 26H), 1.04 – 0.91 (m, 7H), 0.87 – 0.78 (m, 8H), 0.62 (s, 6H). ^{13}C NMR (100 MHz, DMSO-*d*$_6$) δ 170.30, 162.98, 156.70, 132.48, 117.47, 114.57, 69.33, 68.75, 64.56, 62.07, 46.93, 44.48, 44.08, 39.83, 39.72, 33.63, 33.46, 33.21, 32.72, 29.84, 28.71, 26.88, 25.65, 24.53, 21.16, 20.97, 15.40, 12.88, 10.72. MS-ESI: calculated for $[M+H]^+$ ($C_{58}H_{91}O_{11}N_4$)： *m/z* 1019.67, found: *m/z* 1019.87.

Ch-C4

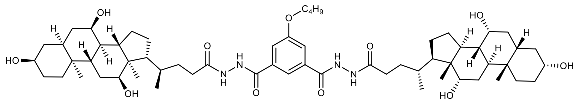

Ch-C4

^{1}H NMR (400 MHz, DMSO-*d*$_6$) δ 10.40 (s, 2H), 9.92 (s, 2H), 7.96 (s, 1H), 7.58 (s, 2H), 4.09 (t, *J* = 6.1 Hz, 2H), 3.82 (s, 4H), 3.63 (s, 8H), 2.21 – 1.97 (m, 10H), 1.81 – 1.65 (m, 12H), 1.49 – 1.22 (m, 26H), 0.97 (dd, *J* = 9.4, 6.8 Hz, 11H), 0.84 (d, *J* = 10.7 Hz, 8H), 0.62 (s, 6H). ^{13}C NMR (100 MHz, DMSO-*d*$_6$) δ 172.45, 165.12, 159.01, 134.61, 119.65, 116.74, 71.48, 70.90, 68.25, 66.71, 49.07, 46.62, 46.23, 41.98, 41.86, 35.78, 35.61, 35.35, 34.86, 31.98, 31.07, 30.86, 29.03, 27.80, 26.67, 23.31, 23.11, 19.17, 17.55, 14.17, 12.87. MS-ESI: calculated for $[M+H]^+$ ($C_{60}H_{95}O_{11}N_4$)： *m/z* 1047.70, found: *m/z* 1047.96.

Ch-C6

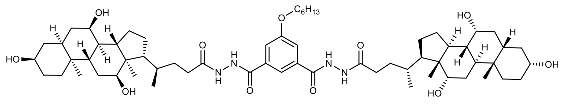

Ch-C6

^{1}H NMR (400 MHz, DMSO-*d*$_6$) δ 10.41 (s, 2H), 9.93 (s, 2H), 7.97 (s, 1H), 7.59 (d, *J* = 1.2 Hz, 2H), 4.09 (d, *J* = 6.4 Hz, 2H), 3.83 (s, 2H), 3.64 (s, 2H), 3.49 (br, 6H), 3.25 – 3.19 (m, 2H), 2.26 – 1.97 (m, 10H), 1.85 - 1.65 (m, 14H), 1.45 - 1.20 (m, 27H), 1.01 – 0.80 (m, 20H), 0.62 (s, 6H). ^{13}C NMR (100 MHz, DMSO-*d*$_6$) δ 172.45, 165.11, 159.01, 134.60, 119.64, 116.73, 71.48, 70.90, 68.53, 66.71, 46.62, 46.23, 41.98, 41.86, 35.78, 35.61, 35.36, 34.86, 31.98, 31.43, 30.86, 29.03, 28.98, 27.80, 26.67, 25.61, 23.31, 23.11, 22.58, 17.55, 14.41, 12.87. MS-ESI: calculated for $[M+H]^+$ ($C_{62}H_{99}O_{11}N_4$)： *m/z* 1075.73, found: *m/z* 1075.98.

Ch-C8

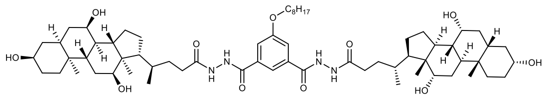

Ch-C8

^{1}H NMR (400 MHz, DMSO-*d*$_6$) δ 10.41 (s, 2H), 9.93 (s, 2H), 7.97 (s, 1H), 7.59 (s, 2H), 4.10 (t, *J* = 6.2 Hz, 2H), 3.83 (s, 2H), 3.64 (s, 2H), 3.52 (br, 6H), 3.26 – 3.16 (m, 2H), 2.26-2.01 (m, 10H), 1.85 - 1.66 (m, 14H), 1.45 – 1.23 (m, 31H), 1.03 – 0.81 (m, 20H), 0.63 (s, 6H). ^{13}C NMR (100 MHz, DMSO-*d*$_6$) δ 172.45, 165.11, 159.01, 134.60, 119.64, 116.73, 71.48,

70.90, 68.53, 66.71, 46.63, 46.23, 41.98, 41.86, 35.78, 35.60, 35.35, 34.86, 31.98, 31.72, 30.86, 29.19, 29.17, 29.02, 27.80, 26.67, 25.94, 23.31, 23.11, 22.58, 17.55, 14.46, 12.87. MS-ESI: calculated for $[M+H]^+$ ($C_{64}H_{103}O_{11}N_4$)： *m/z* 1103.76, found: *m/z* 1104.07.

6-Methoxy-1-(3-sulfonatopropyl) quinolinium (SPQ) Assay

EYPC (0.6 ml, 25 mg/mL in $CHCl_3$, Avanti Polar Lipids, USA) and cholesterol (3.8 mg) were dissolved in $CHCl_3$ (10 mL). $CHCl_3$ was removed under reduced pressure at 35 °C. After drying the resulting film under high vacuum overnight at room temperature, the film was hydrated $NaNO_3$ solution (1.5 mL, 200 mM) containing Cl^--sensitive dye 6-methoxy-N-(3-sulfopropyl)quinolinium (SPQ) (0.5 mM) in thermostatic shaker-incubator at 37 °C for 2 h to give a milky suspension. The mixture was then subjected to 8 freeze-thaw cycles: freezing in liquid N_2 for 30 s and heating at 37 °C for 1.5 mins. The vesicle suspension was extruded through polycarbonate membrane (0.1 μm) to produce a homogeneous suspension of large unilamellar vesicles (LUVs) of about 140 nm in diameter with SPQ encapsulated inside. The suspension of LUVs was dialyzed for 16 h with gentle stirring (300 r/min, 4 °C) using membrane tube (MWCO = 10,000) against the same $NaNO_3$ buffer solution (200 mM, without SPQ) for 6 times to remove the unencapsulated SPQ to yield LUVs with lipids at concentration of 13 mM.

The SPQ-containing LUV suspension (30 μL, 13 mM in 200 mM $NaNO_3$) was added to a NaCl solution (1.75 mL, 200 mM) to create an extravesicular chloride gradient. A solution of **Ch-C1** in DMSO at different concentrations was then injected into the suspension under gentle stirring. Upon the addition of channels, the emission of SPQ was immediately monitored at 430 nm with excitations at 360 nm for 300 seconds using fluorescence spectrophotometer (Hitachi, Model F-7100, Japan) after which time an aqueous solution of Triton X-100 (20 μL, 20% v/v) was immediately added to completely destruct the chloride gradient. The final transport trace was obtained by normalizing the fluorescence intensity using the following Supplementary Equation (1).

$$I_f = (I_t - I_1) / (I_0 - I_1) \qquad (1)$$

where I_f = Fractional emission intensity, I_t = Fluorescence intensity at time t, I_1 = Fluorescence intensity after addition of Triton X-100 and I_0 = Initial fluorescence intensity .

Cation Selectivity Study using HPTS Assay

The HPTS-containing LUV suspension (30 μL, 13 mM in 10 mM HEPES buffer containing 100 mM NaCl at pH = 7.0) was added to a HEPES buffer solution (1.75 mL, 10 mM HEPES, 100 mM MCl at pH = 8.0, where M^+ = Li^+, Na^+, K^+, Rb^+ and Cs^+) to create a pH gradient for ion transport study. A solution of **Ch-C1** at concentration of 5 μM in DMSO was then injected into the suspension under gentle stirring. Upon the addition of **Ch-C1**, the emission of HPTS was immediately monitored at 510 nm with excitations at both 460 and 403 nm recorded simultaneously for 300 seconds using fluorescence spectrophotometer (Hitachi, Model F-7100, Japan) after which time an aqueous solution of Triton X-100 (20 μL, 20% v/v) was immediately added to achieve the maximum change in dye fluorescence emission. The final transport trace was obtained as a ratiometric value of I_{460}/I_{403} and normalized based on the ratiometric value of I_{460}/I_{403} after addition of triton.

Dynamic Light Scattering Assay

Dynamic light scattering (DLS) experiments were perfomed on a NanoBrook Omni Particle Size Analyzer (Brookhaven Instrumennts Corporation, Holtsville, NY). In a typical experiment, the cholesterol-containing LUVs suspension (30 μL, 13 mM in 10 mM HEPES buffer containing 100 mM NaCl at pH = 7.5) was diluted to a HEPES buffer solution (1.75 mL, 10 mM HEPES, 100 mM NaCl at pH = 7.5). A solution of channels in DMSO was then added into the solution, which was equilibrated with gentle shaking at 25 ^{o}C for 5 minutes before measurement. The hydrodynamic sizes of LUVs were recoreded as intensity mean from the average of three DLS experiments at 25 ^{o}C.

Molecular Dynamics Simulations

The structural models were built using Gaussian 09, and then optimized using the generalized amber force field (GAFF).[1] The partial charges were fitted based on Restrained Electrostatic Potential (RESP) method in which the electric potential was calculated with Gaussian 09 at the level of HF/6-31G*. All MD simulations were performed with GROMACS package (version 4.6.3)[2] for 200 ns at 300 K. A leap-frog algorithm was used to integrate Newton's equation of motion, and the time step was set to 2 fs along with the application of LINCS algorithm[3] by which all the covalent bonds between hydrogen atoms and heavy atoms were constrained. Simulation systems were solvated in TIP3P water model.[4] Sodium and chloride ions, characterized by Joung et al.,[5] were used as the counter ions and added to reach a concentration of 0.17 M. A cutoff of 1.2 nm was used for both van der Waals interaction calculation and short-range electrostatic interaction calculation, and correspondingly Particle mesh Ewald (PME)9[6] method was employed to deal with the long-range electrostatic interaction. All the visualization work was done by PyMol, version 1.5.0.3. The POPC lipid bilayer was modeled by all atom amber lipid14 force field.[7]

In vitro Hemolytic Activity Assessment

Mice blood was isolated in a sterile environment and diluted to a 4% concentration with HEPES buffer as RBCs solution. **Ch-C1** was mixed with diluted RBCs into different concentrations of drug/erythrocyte mixture and transferred to a 96-well plate, with the HEPES-free buffer and 0.5% Triton-X (Adamas) set as negative and positive controls, respectively. After 24 h culturing at 37°C, the 96-well plate was placed in a centrifuge at 448 × g for 5 min. The supernatant was transferred to a new 96-well plate, and the OD value at 576 nm was measured by a microplate reader. The hemolytic activity was calculated following Supplementary Equation (2).

$$\%\text{Hemolytic Activity}=\frac{OD_{576}(\textbf{Ch-C1})\text{-}OD_{576}(\text{HEPES})}{OD_{576}(\text{Triton-X})\text{-}OD_{576}(\text{HEPES})}\times 100\% \quad (2)$$

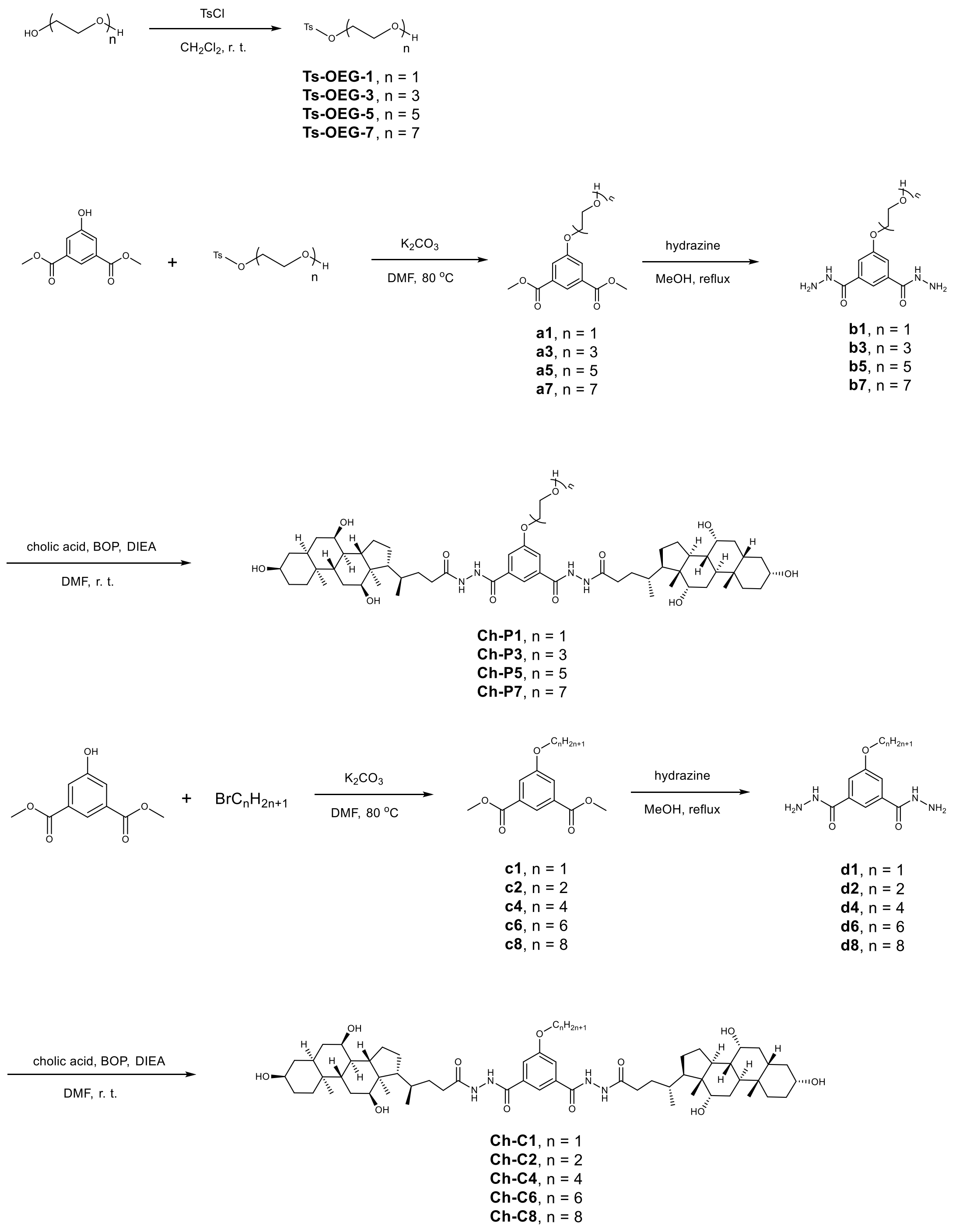

Supplementary Fig. 1 Synthesis of channels. Synthetic route that affords channel molecules.

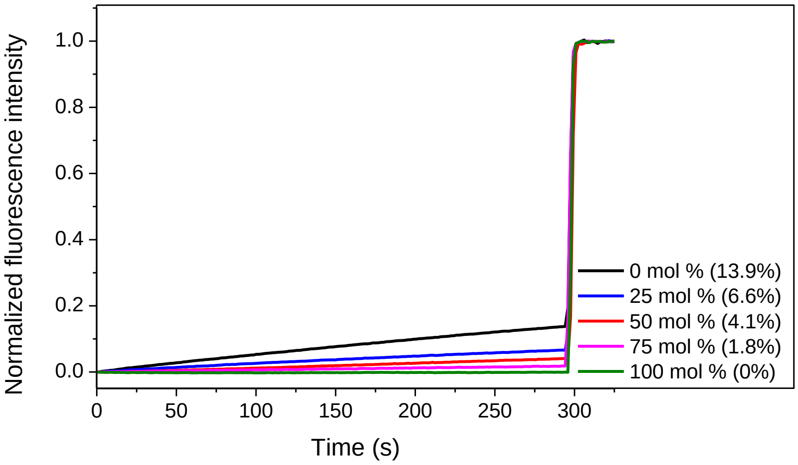

Supplementary Fig. 2 Background of HPTS assay. Background signals for the LUVs containing different contents of cholesterol (0, 25, 50, 75, 100 mol% relative to lipid) in the HPTS assay. HPTS = 8-hydroxy-1,3,6-pyrenetrisulfonate; LUV = large unilamellar vesicles. Source data are provided as a Source Data file.

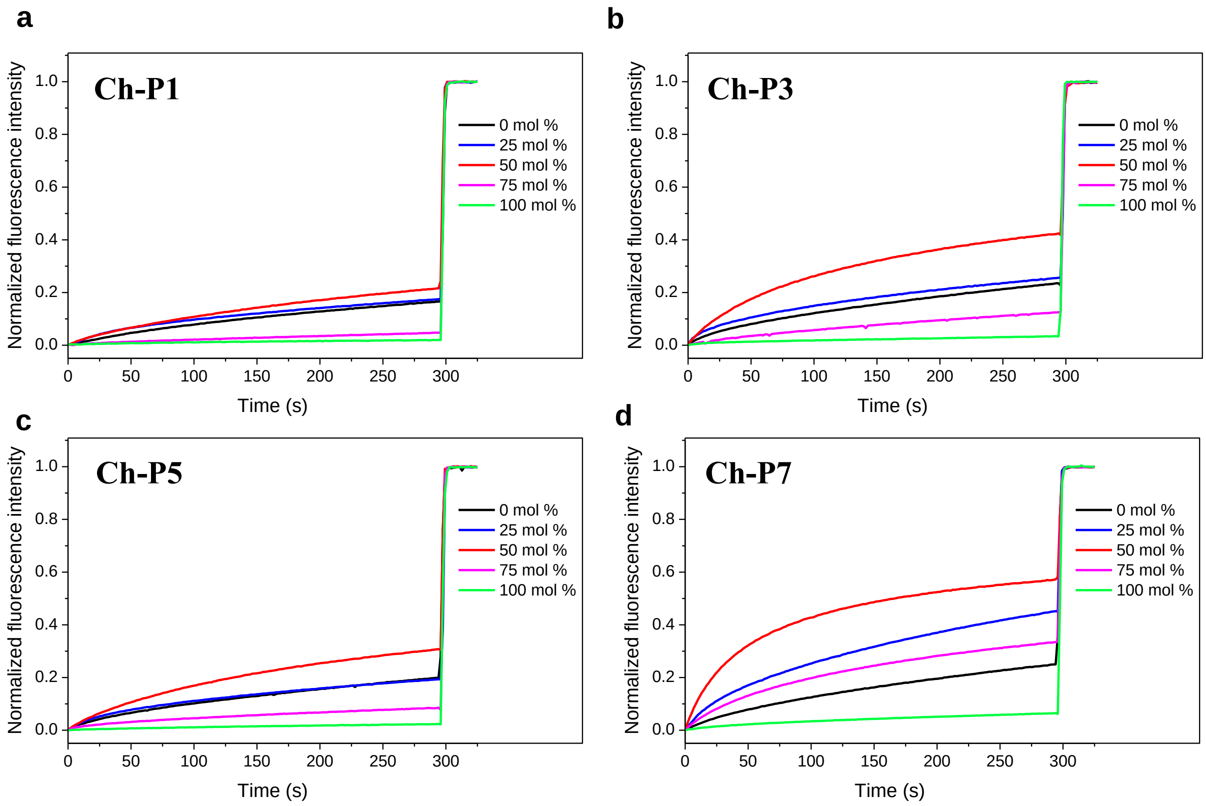

Supplementary Fig. 3 HPTS assay for transmembrane transport activity study of nanopores. Ion transport curves of **a Ch-P1**, **b Ch-P3**, **c Ch-P5** and **d Ch-P7** at concentration of 8 μM using LUVs containing different contents of cholesterol (0, 25, 50, 75, 100 mol% relative to lipid) in the HPTS assay. HPTS = 8-hydroxy-1,3,6-pyrenetrisulfonate; LUV = large unilamellar vesicles. The ratiometric values of I_{460}/I_{403} were normalized based on the value of I_{460}/I_{403} at t = 300 s right after addition of triton. Source data are provided as a Source Data file.

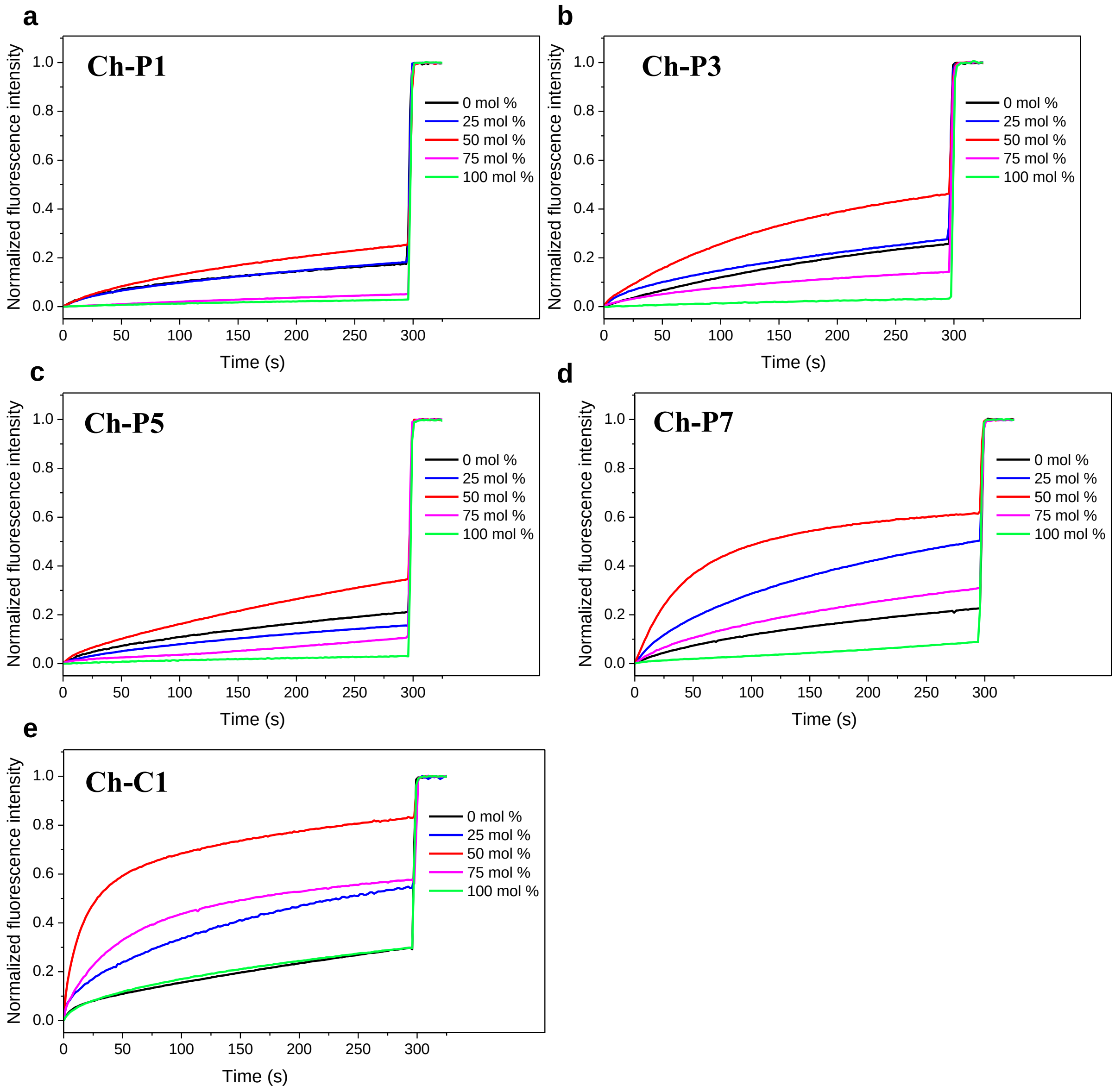

Supplementary Fig. 4 HPTS assay for transmembrane transport activity study of nanopores. Ion transport curves of **a Ch-P1**, **b Ch-P3**, **c Ch-P5**, **d Ch-P7** and **e Ch-C1** at concentration of 8 μM using LUVs containing different contents of cholesterol (0, 25, 50, 75, 100 mol% relative to lipid) in a repeated HPTS assay. HPTS = 8-hydroxy-1,3,6-pyrenetrisulfonate; LUV = large unilamellar vesicles. The ratiometric values of I_{460}/I_{403} were normalized based on the value of I_{460}/I_{403} at t = 300 s right after addition of triton. Source data are provided as a Source Data file.

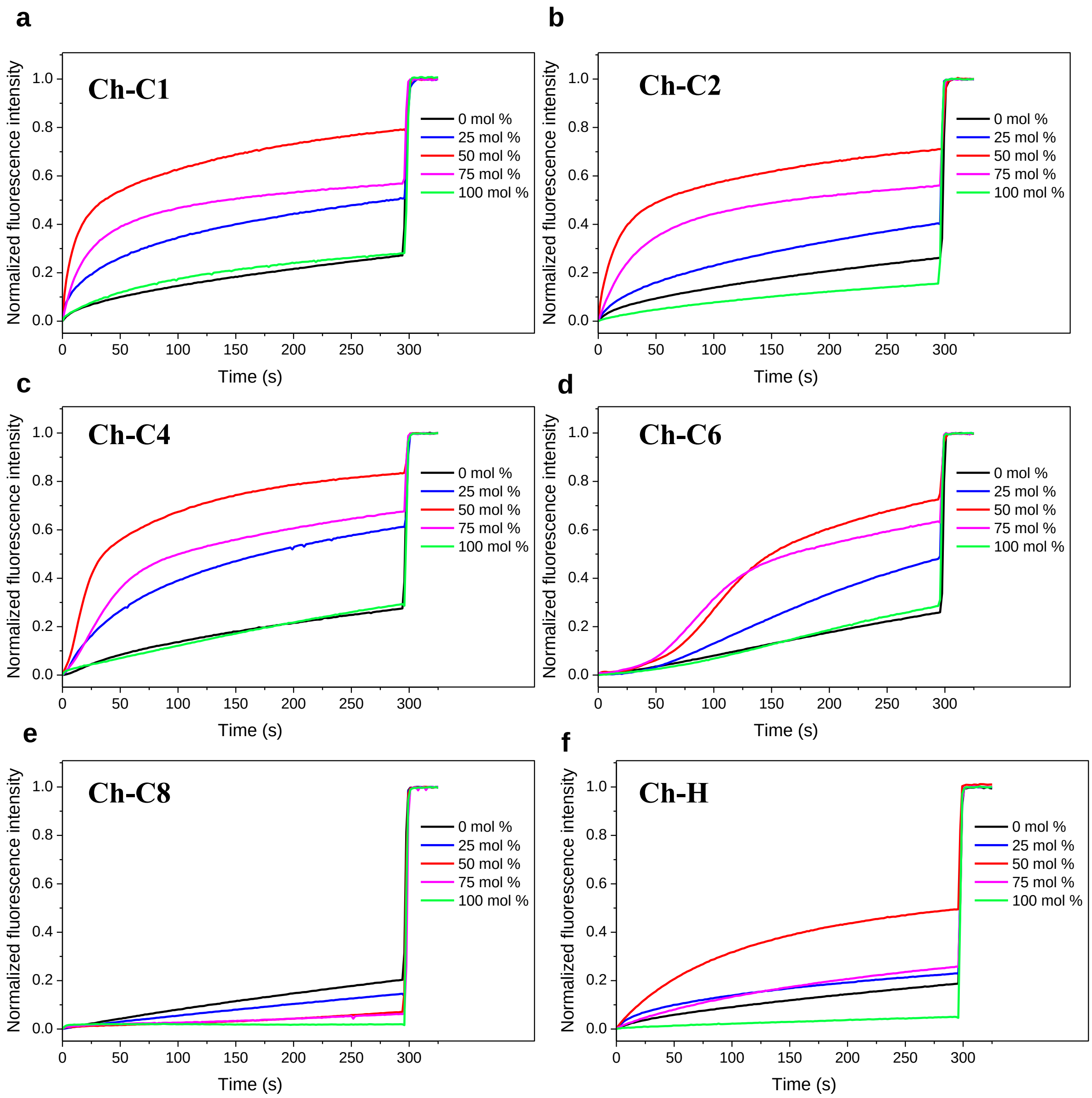

Supplementary Fig. 5 HPTS assay for transmembrane transport activity study of nanopores. Ion transport curves of **a Ch-C1**, **b Ch-C2**, **c Ch-C4**, **d Ch-C6**, **e Ch-C8** and **f Ch-H** at concentration of 8 μM using LUVs containing different contents of cholesterol (0, 25, 50, 75, 100 mol% relative to lipid) in the HPTS assay. HPTS = 8-hydroxy-1,3,6-pyrenetrisulfonate; LUV = large unilamellar vesicles. The ratiometric values of I_{460}/I_{403} were normalized based on the value of I_{460}/I_{403} at t = 300 s right after addition of triton. Source data are provided as a Source Data file.

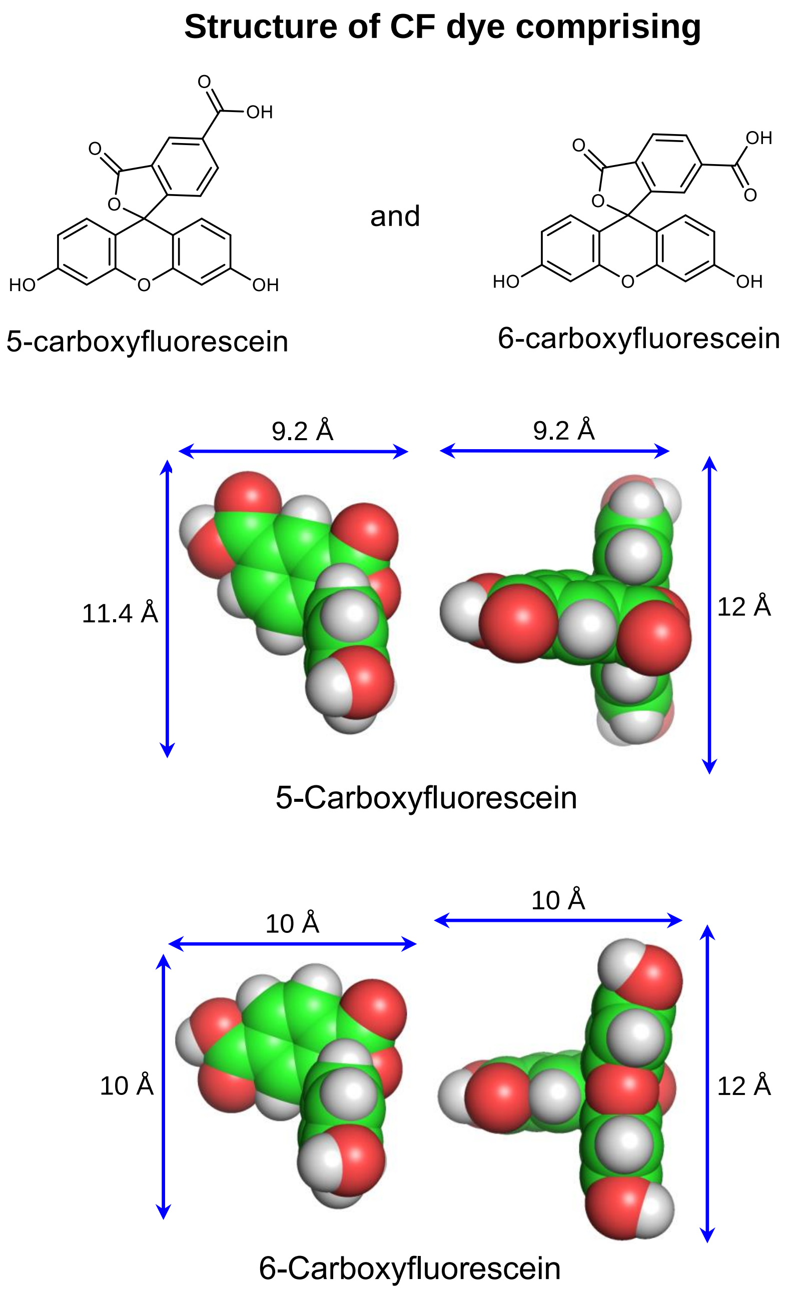

Supplementary Fig. 6 Molecular structure of CF dye. Chemical structures and CPK models of 5(6)-Carboxyfluorescein isomers among which the smaller dimensions are 10 Å x 10 Å rather than 9 Å x 11 Å in order to pass the pores. CPK = Corey-Pauling-Koltun.

CF Dye Leakage Assay using Cholesterol-Containing LUVs

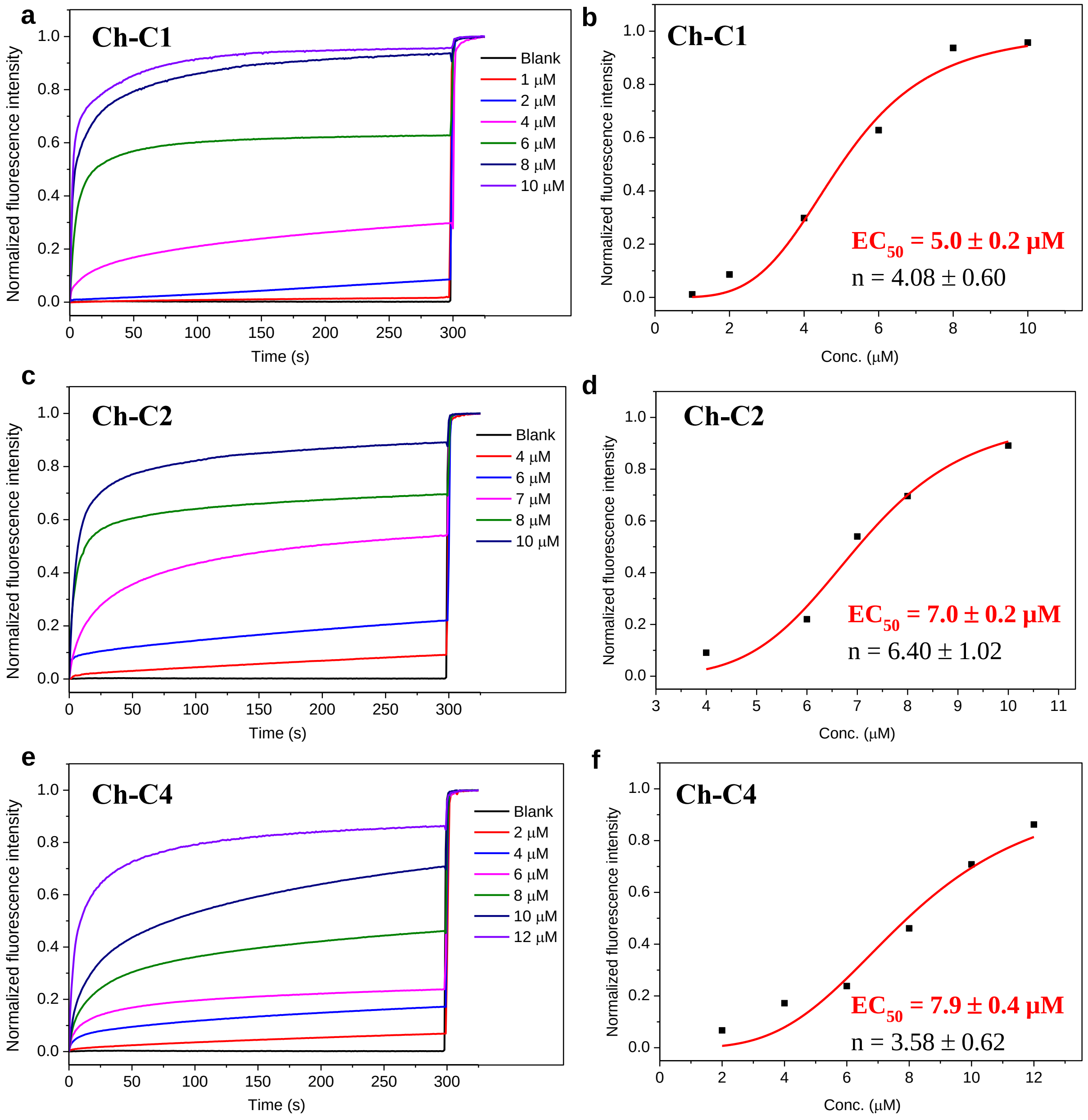

Supplementary Fig. 7 CF dye leakage assay for transmembrane transport activity study of nanopore. Fluorescence intensity changes of CF dye (λ_{ex}=492 nm, λ_{em}=517 nm) after additions of **a**, **b Ch-C1**, **c**, **d Ch-C2** and **e**, **f Ch-C4** at different concentrations and the corresponding EC_{50} values for efflux of CF dye. CF = 5(6)-carboxyfluorescein. Inside LUV: 10 mM HEPES, 500 mM CF, pH = 7.5; outside LUV: 10 mM HEPES, pH = 7.5. LUV = large unilamellar vesicles. Source data are provided as a Source Data file.

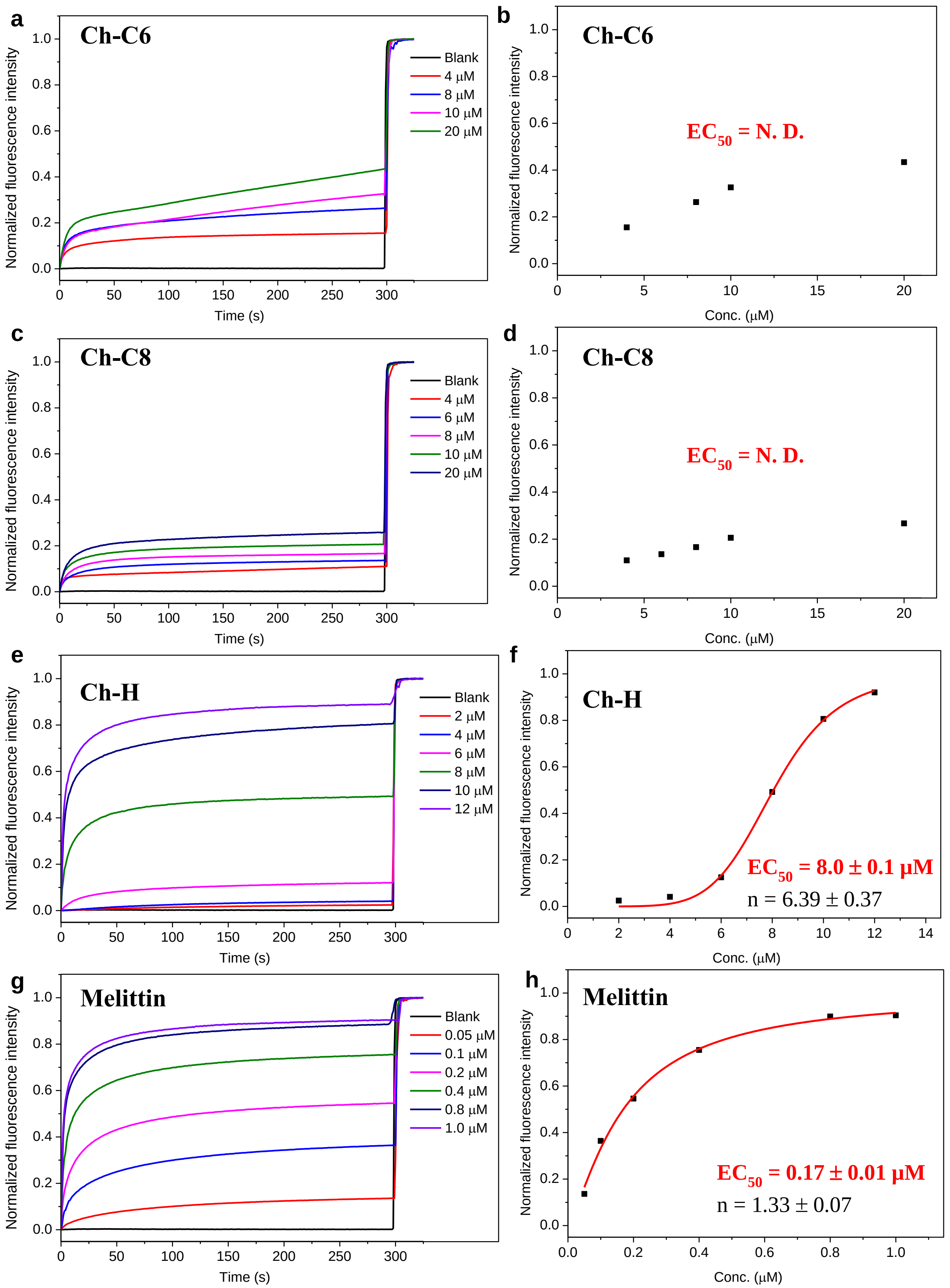

Supplementary Fig. 8 CF dye leakage assay for transmembrane transport activity study of nanopore. Fluorescence intensity changes of CF dye (λ_{ex}=492 nm, λ_{em}=517 nm) after additions of **a**, **b Ch-C6**, **c**, **d Ch-C8**, **e**, **f Ch-H** and **g**, **h** Melittin at different concentrations and determination of corresponding EC_{50} values for efflux of CF dye. CF = 5(6)-carboxyfluorescein. Inside LUV: 10 mM HEPES, 500 mM CF, pH = 7.5; outside LUV: 10 mM HEPES, pH = 7.5. LUV = large unilamellar vesicles. Source data are provided as a Source Data file.

CF Dye Leakage Assay using Cholesterol-Free LUVs

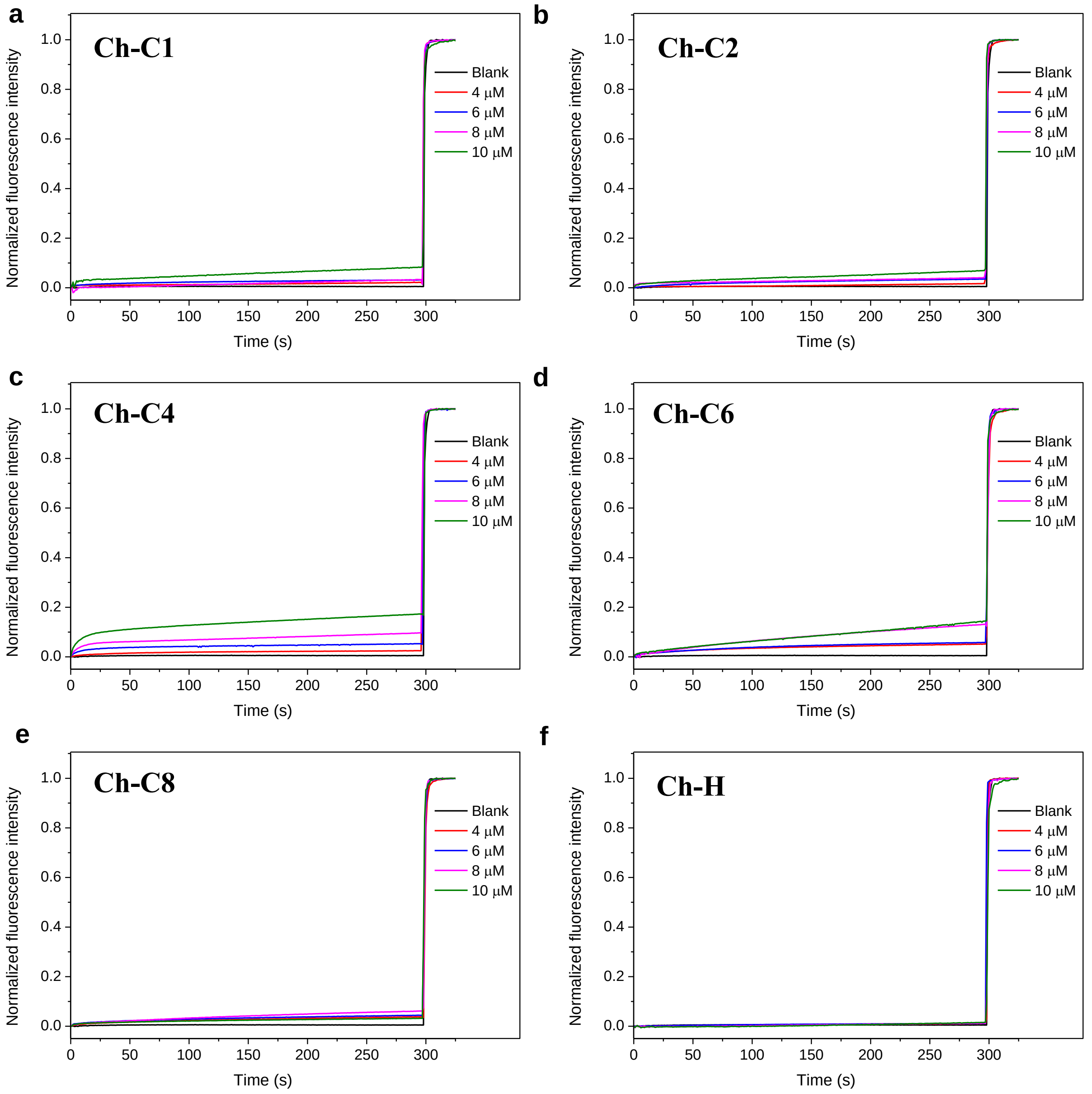

Supplementary Fig. 9 CF dye leakage assay for transmembrane transport activity study of nanopores in the absence of cholesterol. Fluorescence intensity changes of CF dye (λ_{ex}= 492 nm, λ_{em}= 517 nm) after additions of **a Ch-C1**, **b Ch-C2**, **c Ch-C4**, **d Ch-C6**, **e Ch-C8** and **f Ch-H** at different concentrations in the absence of cholesterol. CF = 5(6)-carboxyfluorescein. These experiments demonstrate an important role played by cholesterol in the membrane to aid the channel formation. Inside LUV: 10 mM HEPES, 500 mM CF, pH = 7.5; outside LUV: 10 mM HEPES, pH = 7.5. LUV = large unilamellar vesicles. Source data are provided as a Source Data file.

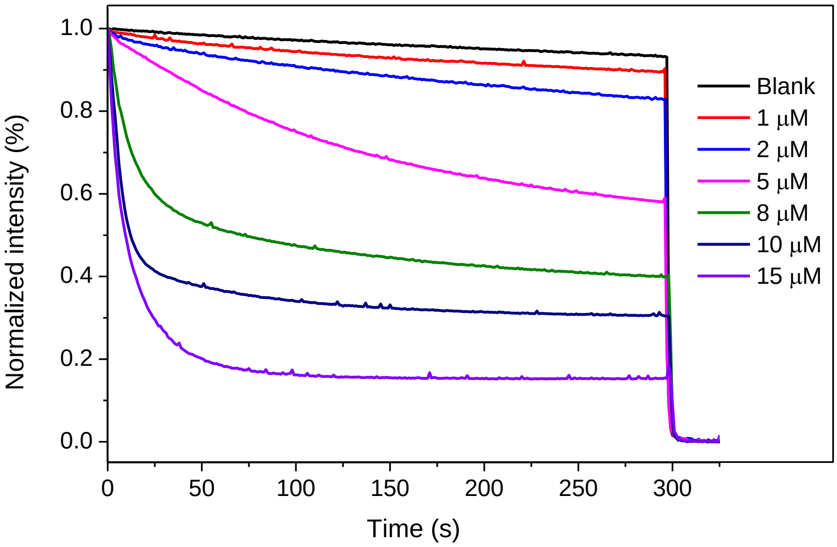

Supplementary Fig. 10 SPQ assay for anion transport activity study of nanopores. Normalized fluorescence intensity changes of the chloride-sensitive SPQ dye (excitation wavelength: 360 nm; emission wavelength: 430 nm) after the addition of **Ch-C1** at different concentrations in the presence of cholesterol (50 mol% relative to lipid). Inside LUV: 200 mM $NaNO_3$ and 0.5 mM SPQ. SPQ = 6-methoxy-N-(3-sulfopropyl) quinolinium. Outside LUV: 200 mM NaCl. LUV = large unilamellar vesicles. Source data are provided as a Source Data file.

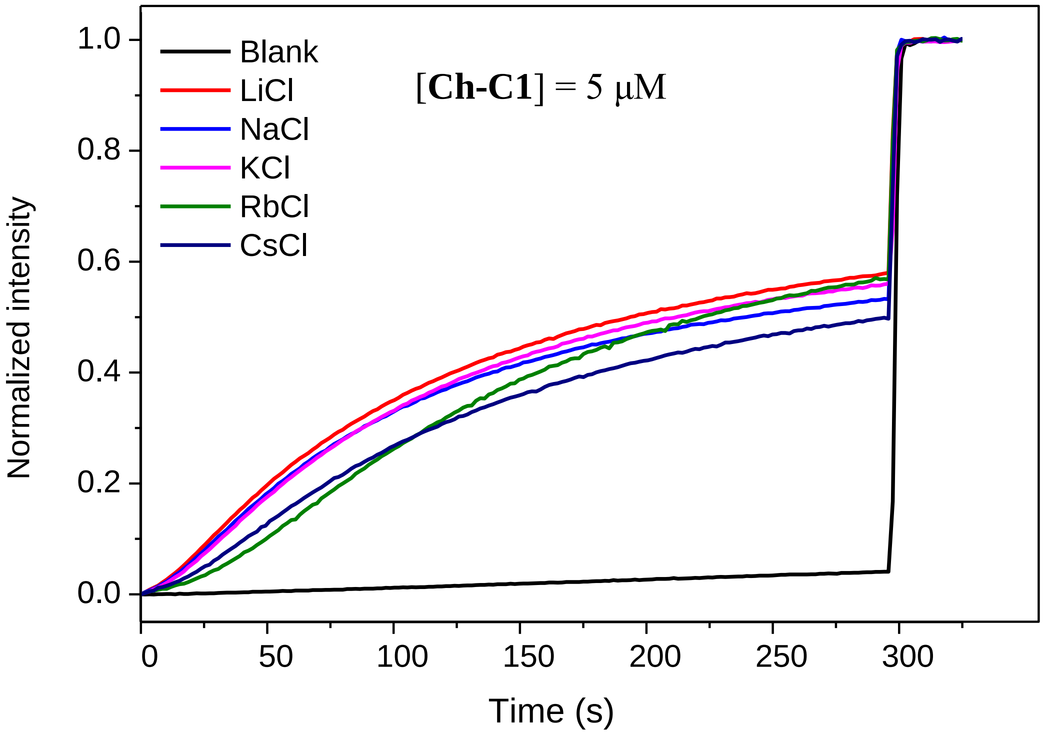

Supplementary Fig. 11 HPTS assay for cation transport activity study of nanopore. Transport selectivity of alkali metal ions by **Ch-C1** (5 μM) in the presence of cholesterol (50 mol% relative to lipid) obtained from the HPTS assay by varying the extravesicular MCl ($M^+ = Li^+$, Na^+, K^+, Rb^+ and Cs^+). HPTS = 8-hydroxy-1,3,6-pyrenetrisulfonate. Source data are provided as a Source Data file.

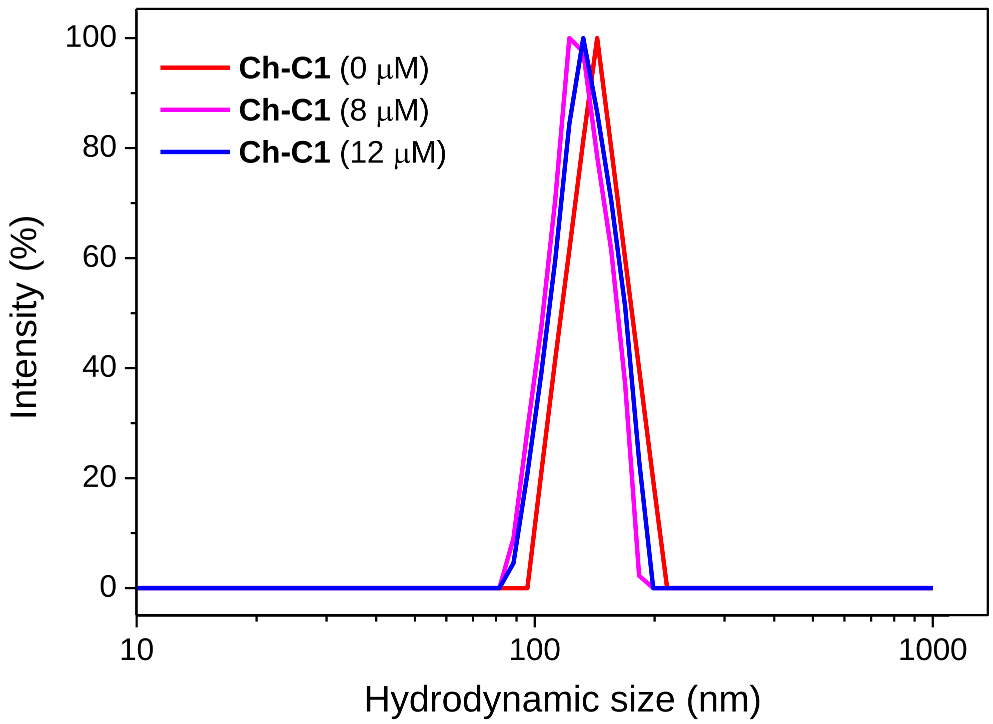

Supplementary Fig. 12 Membrane integrity study. The hydrodynamic size distributions of cholesterol-containing LUVs before and after the incorporation of 8 or 12 µM of **Ch-C1** (3.7 and 5.6 mol% relative to lipids, respectively). The marginable changes in the hydrodynamic size of LUVs upon the incorporation of different concentration of **Ch-C1** rule out the possible re-assembly of LUVs to yield LUVs of significantly smaller or larger sizes. LUV = large unilamellar vesicles. Source data are provided as a Source Data file.

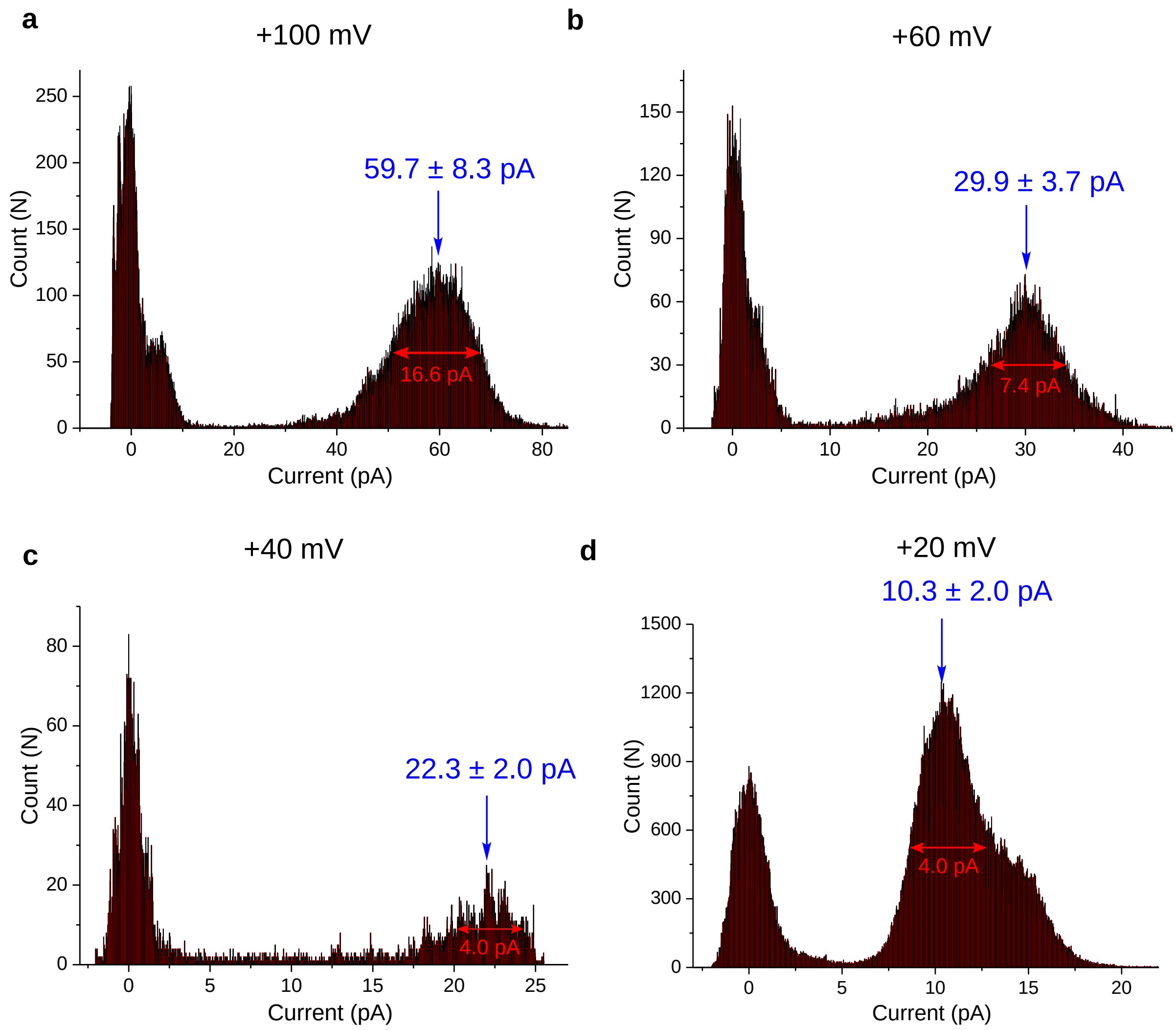

Supplementary Fig. 13 Single channel study of nanopores. Histograms of single channel currents of **Ch-C1** recorded in symmetric baths (*cis* chamber = *trans* chamber = 1 M KCl) at transmembrane potentials of **a** 100, **b** 60, **c** 40 and **d** 20 mv. Relative errors were obtained by dividing the width between the two half height points by 2. Source data are provided as a Source Data file.

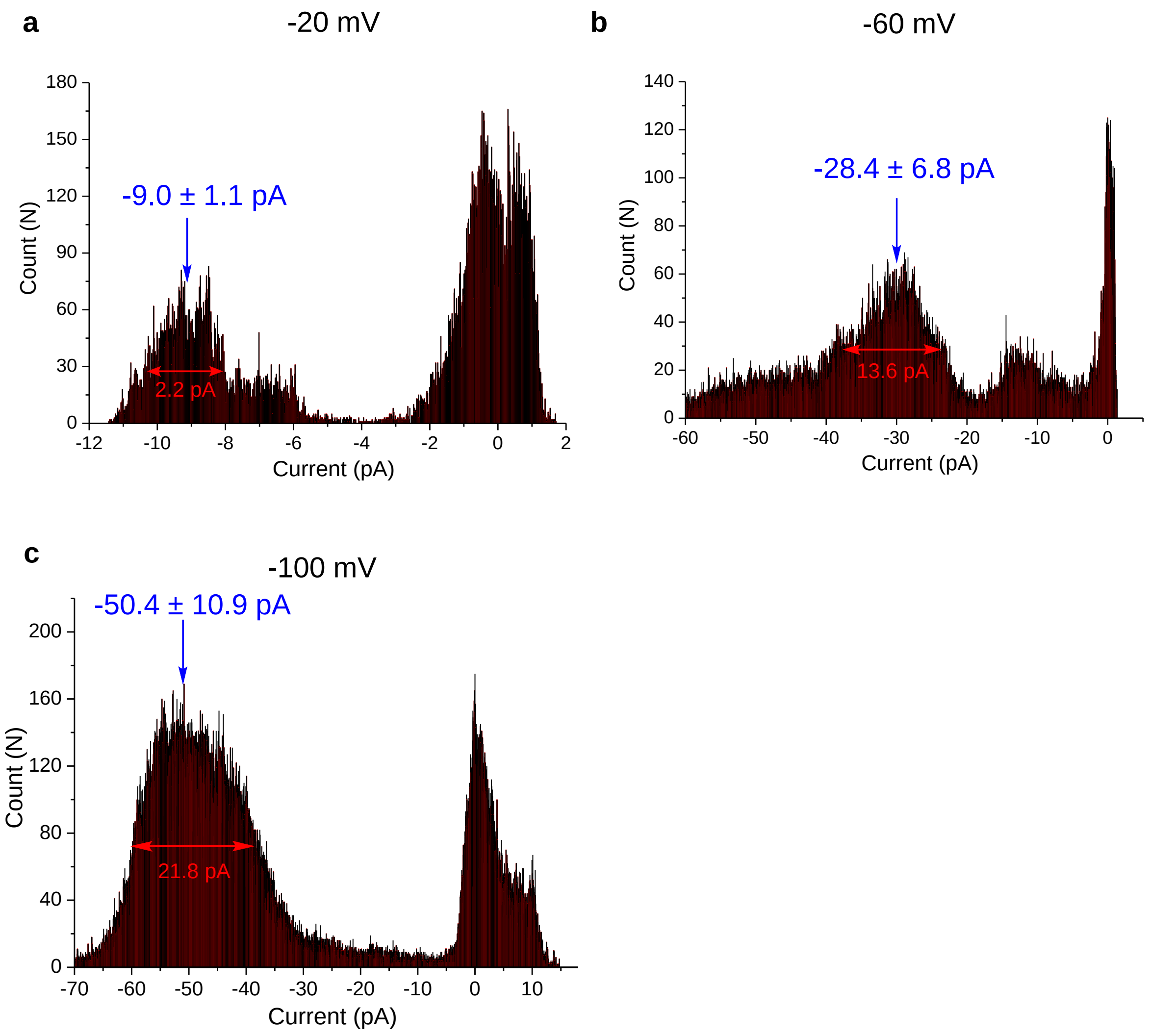

Supplementary Fig. 14 Single channel study of nanopores. Histograms of single channel currents of **Ch-C1** recorded in symmetric baths (*cis* chamber = *trans* chamber = 1 M KCl) at transmembrane potentials of **a** -20, **b** -60 and **c** -100 mv. Relative errors were obtained by dividing the width between the two half height points by 2. Source data are provided as a Source Data file.

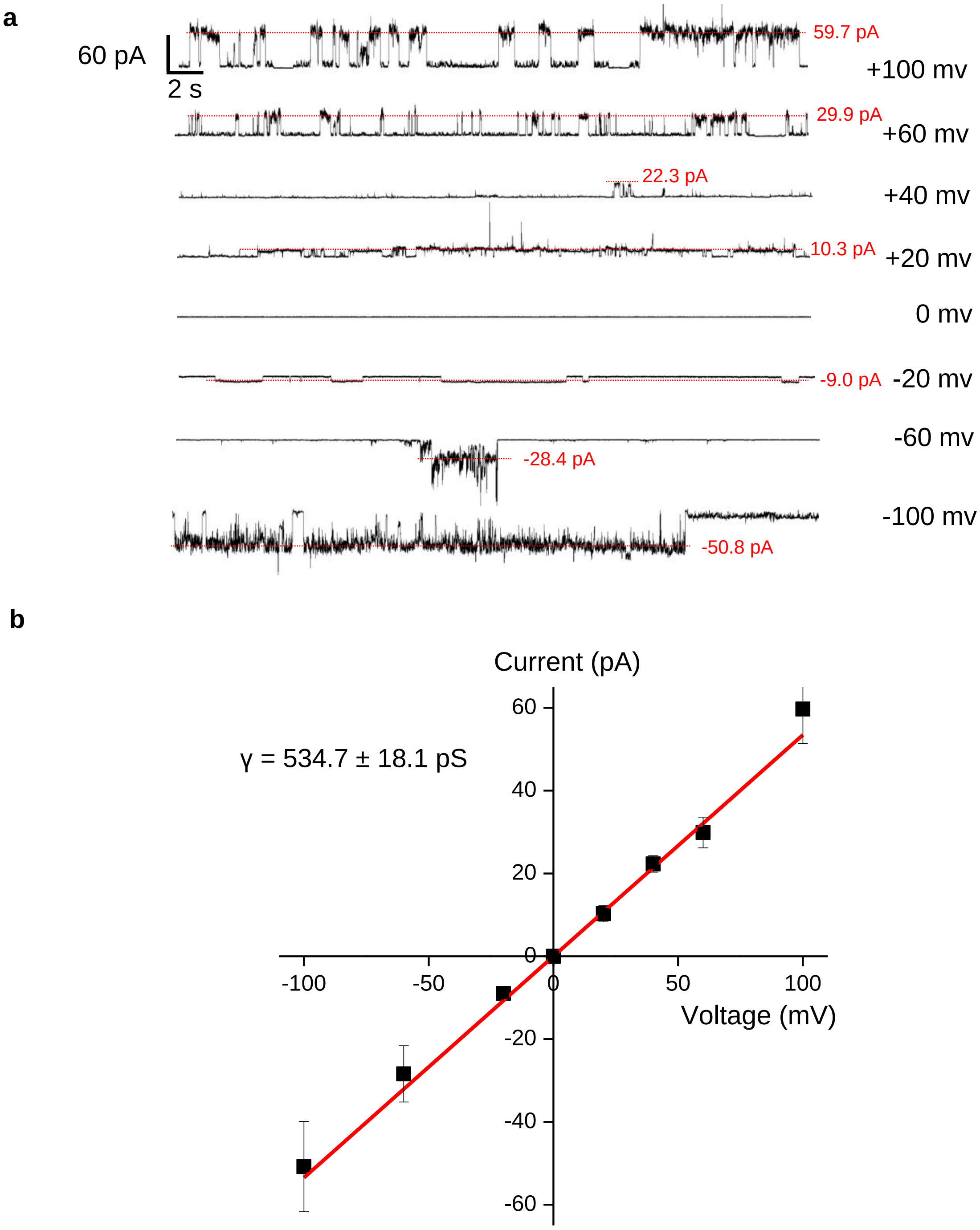

Supplementary Fig. 15 Single channel study of nanopores. a Single channel current traces of **Ch-C1** recorded at various voltages in symmetric baths (*cis* chamber = *trans* chamber = 1 M KCl). Dotted red lines refer to the mean current values that were obtained from histograms and used to obtain the conductance value. **b** Current-voltage (I-V) curve for obtaining the ion conductance (γ) for **Ch-C1**. The pore size was estimated to be 1.67 nm. Data are represented as mean current values ± half width at half maximum. n = 3 independent experiments. Source data are provided as a Source Data file.

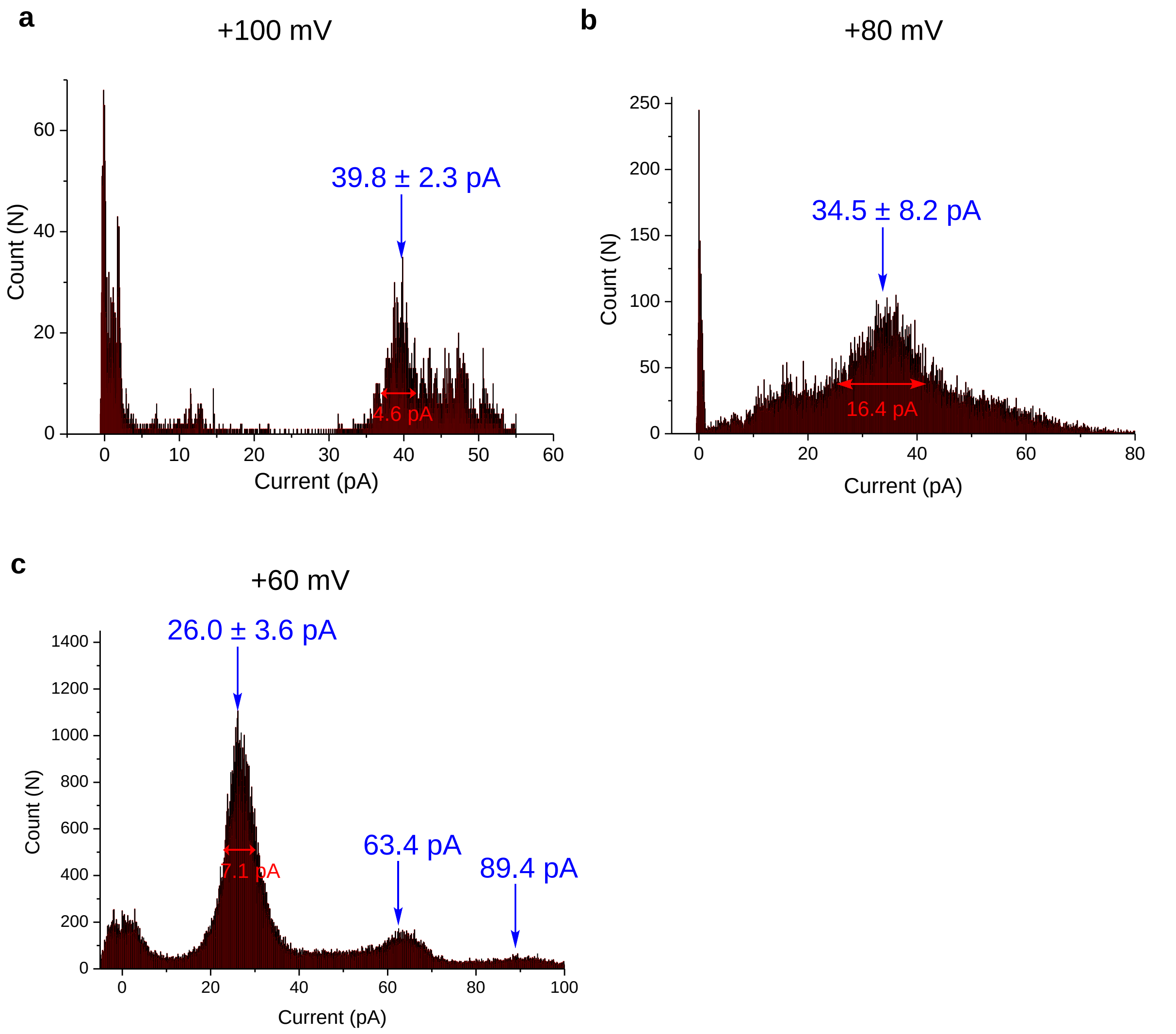

Supplementary Fig. 16 Single channel study of nanopores. Histograms of single channel currents of **Ch-C1** recorded in symmetric baths (*cis* chamber = *trans* chamber = 1 M KCl) at transmembrane potentials of **a** 100, **b** 80 and **c** 60 mv in a repeated assay. Relative errors were obtained by dividing the width between the two half height points by 2. Source data are provided as a Source Data file.

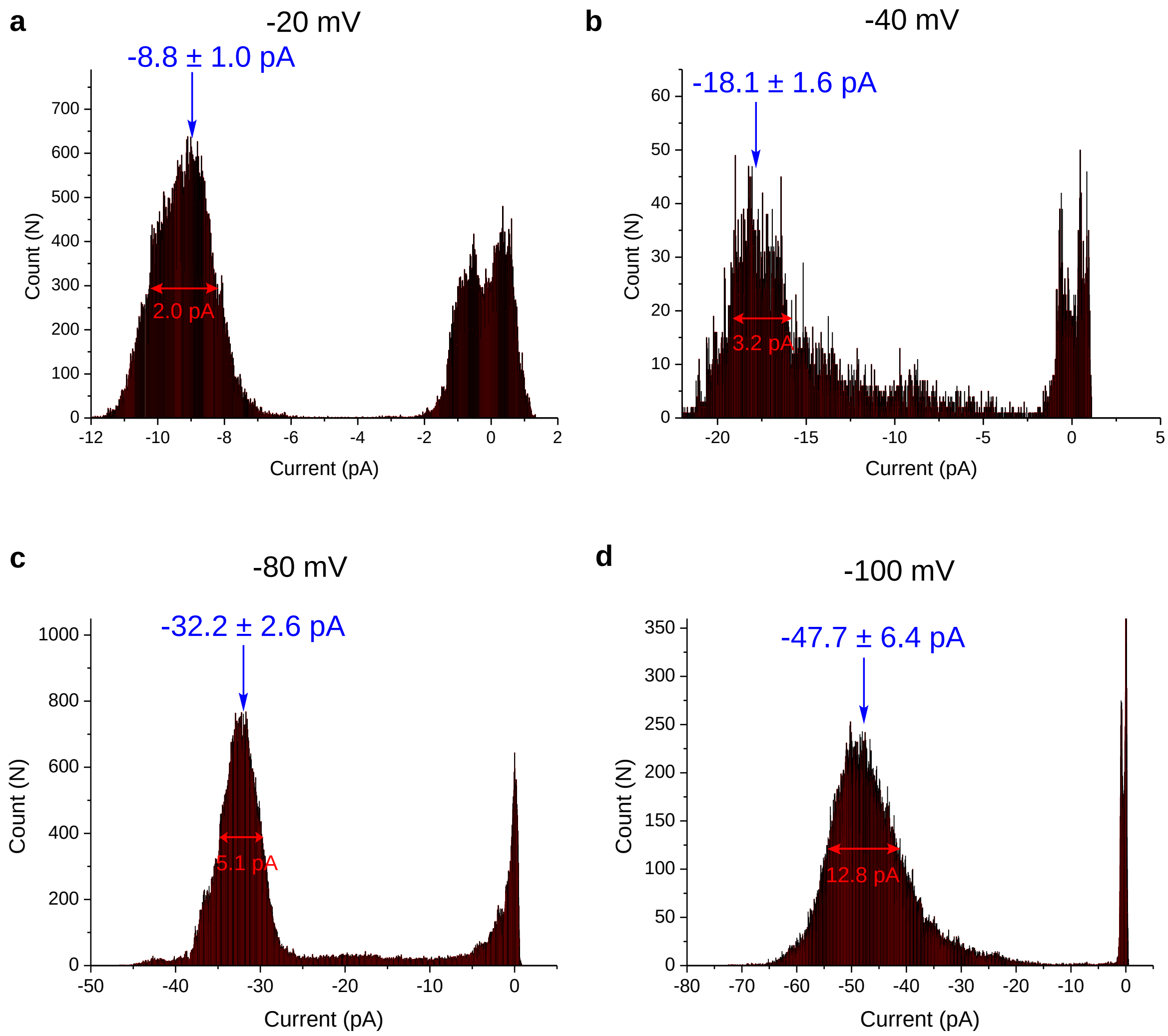

Supplementary Fig. 17 Single channel study of nanopores. Histograms of single channel currents of **Ch-C1** recorded in symmetric baths (*cis* chamber = *trans* chamber = 1 M KCl) at transmembrane potentials of **a** -20, **b** -40, **c** -80 and **d** -100 mv in a repeated assay. Relative errors were obtained by dividing the width between the two half height points by 2. Source data are provided as a Source Data file.

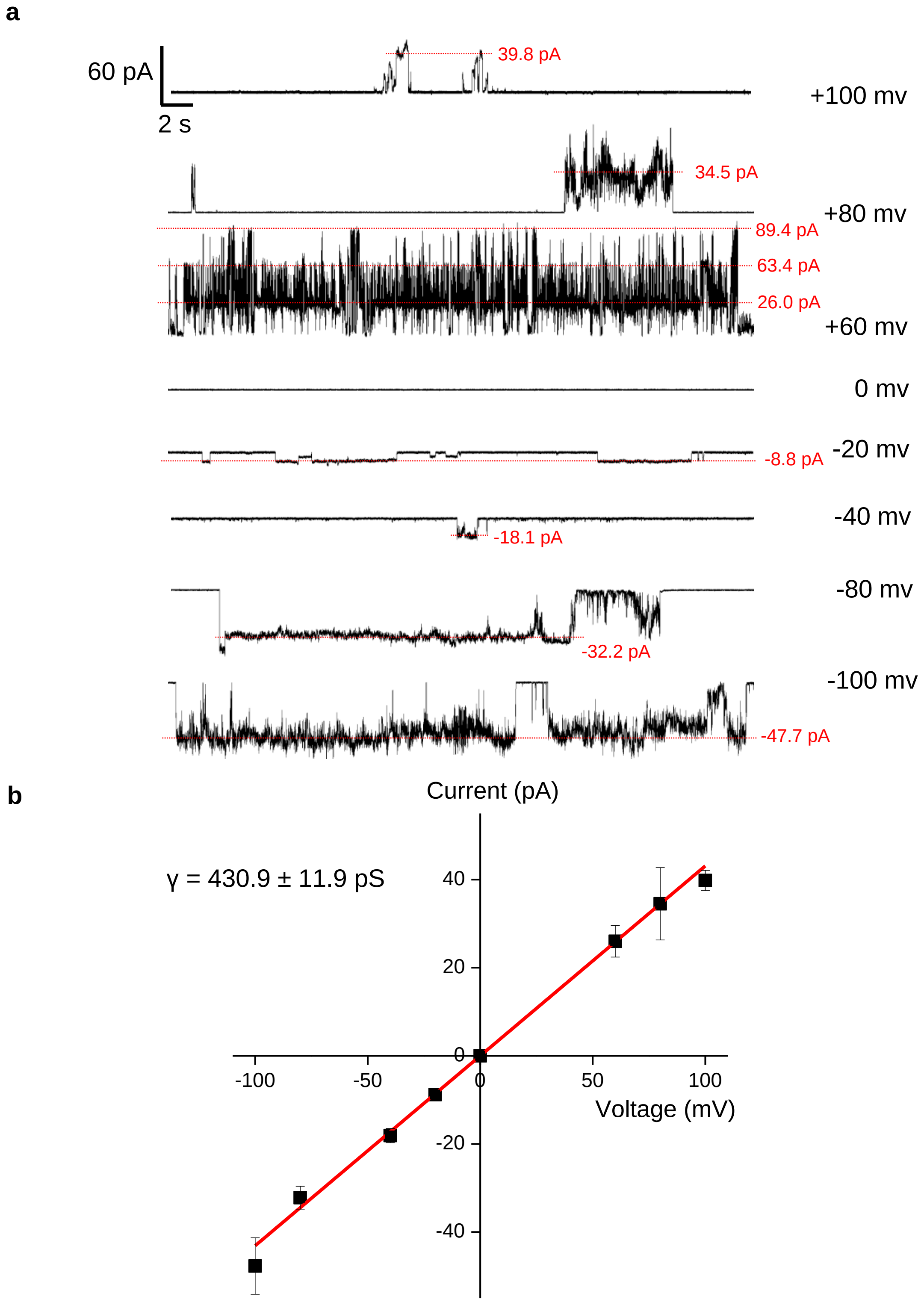

Supplementary Fig. 18 Single channel study of nanopores. a Single channel current traces of **Ch-C1** recorded at various voltages in symmetric baths (*cis* chamber = *trans* chamber = 1 M KCl) in a repeated assay. Dotted red lines refer to the mean current values that were obtained from histograms and used to obtain the conductance value. **b** Current-voltage (I-V) curve for obtaining the ion conductance (γ) for **Ch-C1**. The pore size was estimated to be 1.48 nm. Data are represented as mean current values ± half width at half maximum. n = 3 independent experiments. Source data are provided as a Source Data file.

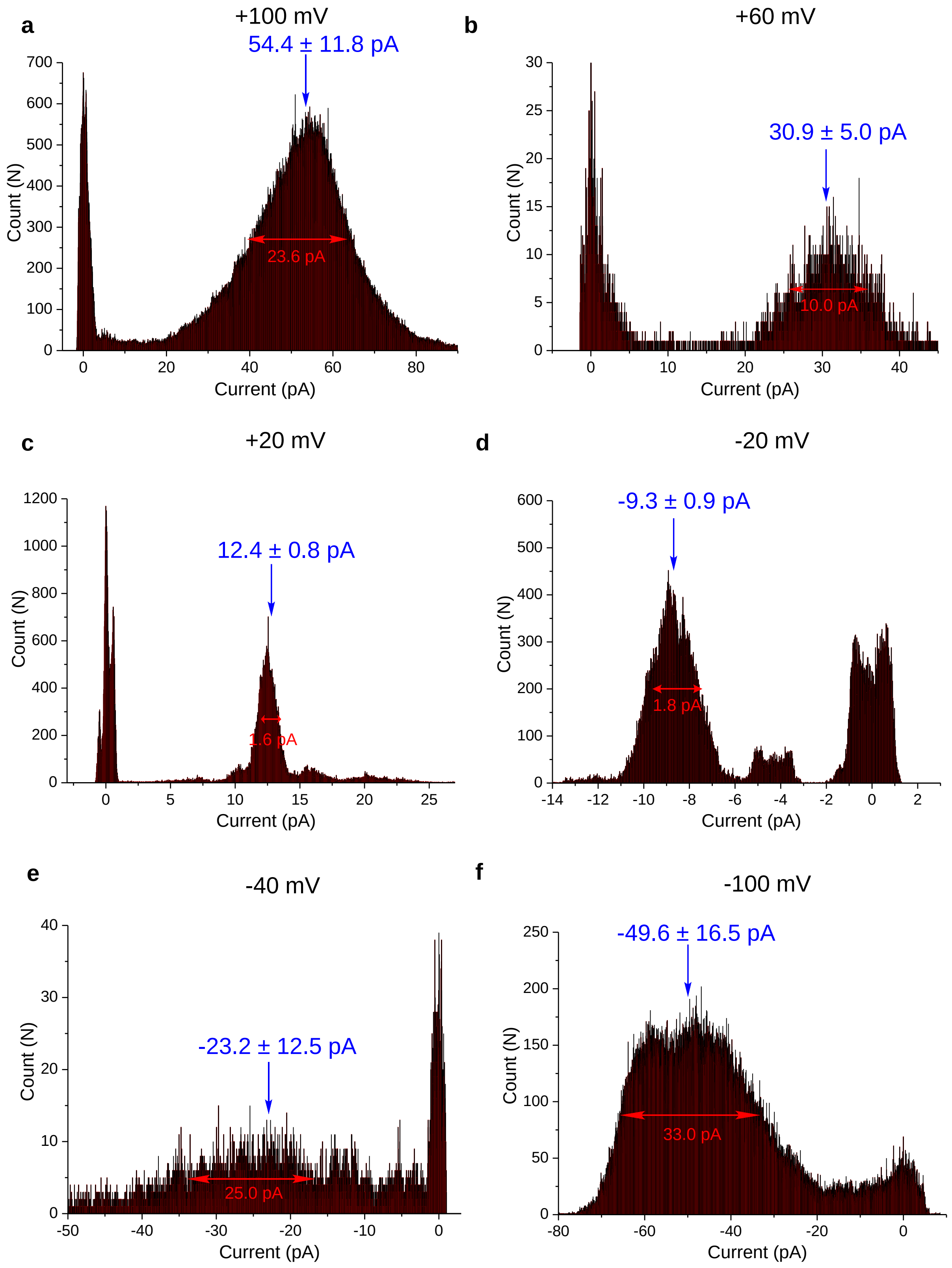

Supplementary Fig. 19 Single channel study of nanopores. Histograms of single channel currents of **Ch-C1** recorded in symmetric baths (*cis* chamber = *trans* chamber = 1 M KCl) at transmembrane potentials of **a** 100, **b** 60, **c** 20, **d** -20, **e** -40 and **f** -100 mv in a repeated assay. Relative errors were obtained by dividing the width between the two half height points by 2. Source data are provided as a Source Data file.

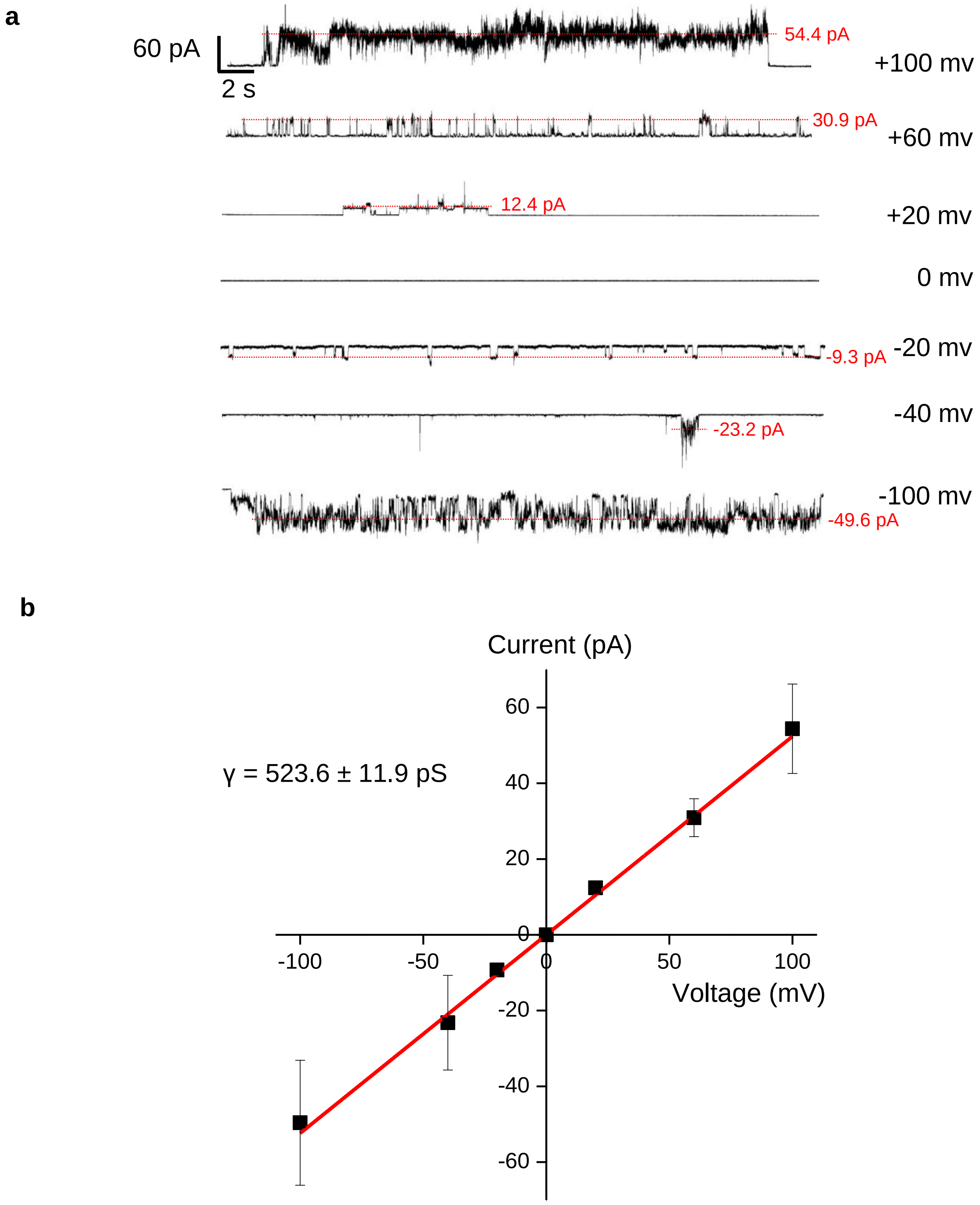

Supplementary Fig. 20 Single channel study of nanopores. a Single channel current traces of **Ch-C1** recorded at various voltages in symmetric baths (*cis* chamber = *trans* chamber = 1 M KCl) in a repeated assay. Dotted red lines refer to the mean current values that were obtained from histograms and used to obtain the conductance value. **b** Current-voltage (I-V) curve for obtaining the ion conductance (γ) for **Ch-C1**. The pore size was estimated to be 1.65 nm. Data are represented as mean current values ± half width at half maximum. n = 3 independent experiments. Source data are provided as a Source Data file.

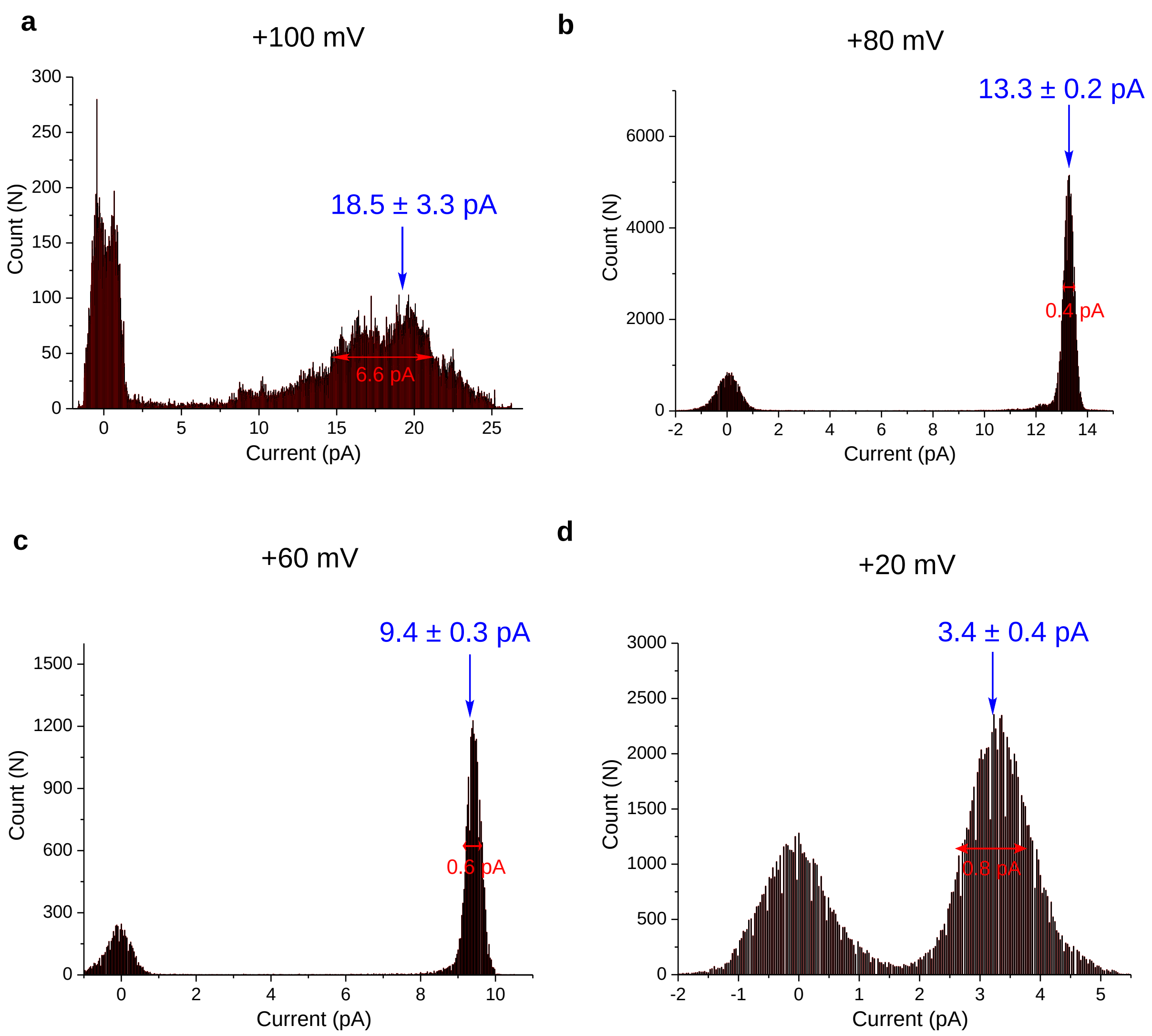

Supplementary Fig. 21 Single channel study of nanopores. Histograms of single channel currents of **Ch-C4** recorded in symmetric baths (*cis* chamber = *trans* chamber = 1 M KCl) at transmembrane potentials of **a** 100, **b** 80, **c** 60 and **d** 20 mv. Relative errors were obtained by dividing the width between the two half height points by 2. Source data are provided as a Source Data file.

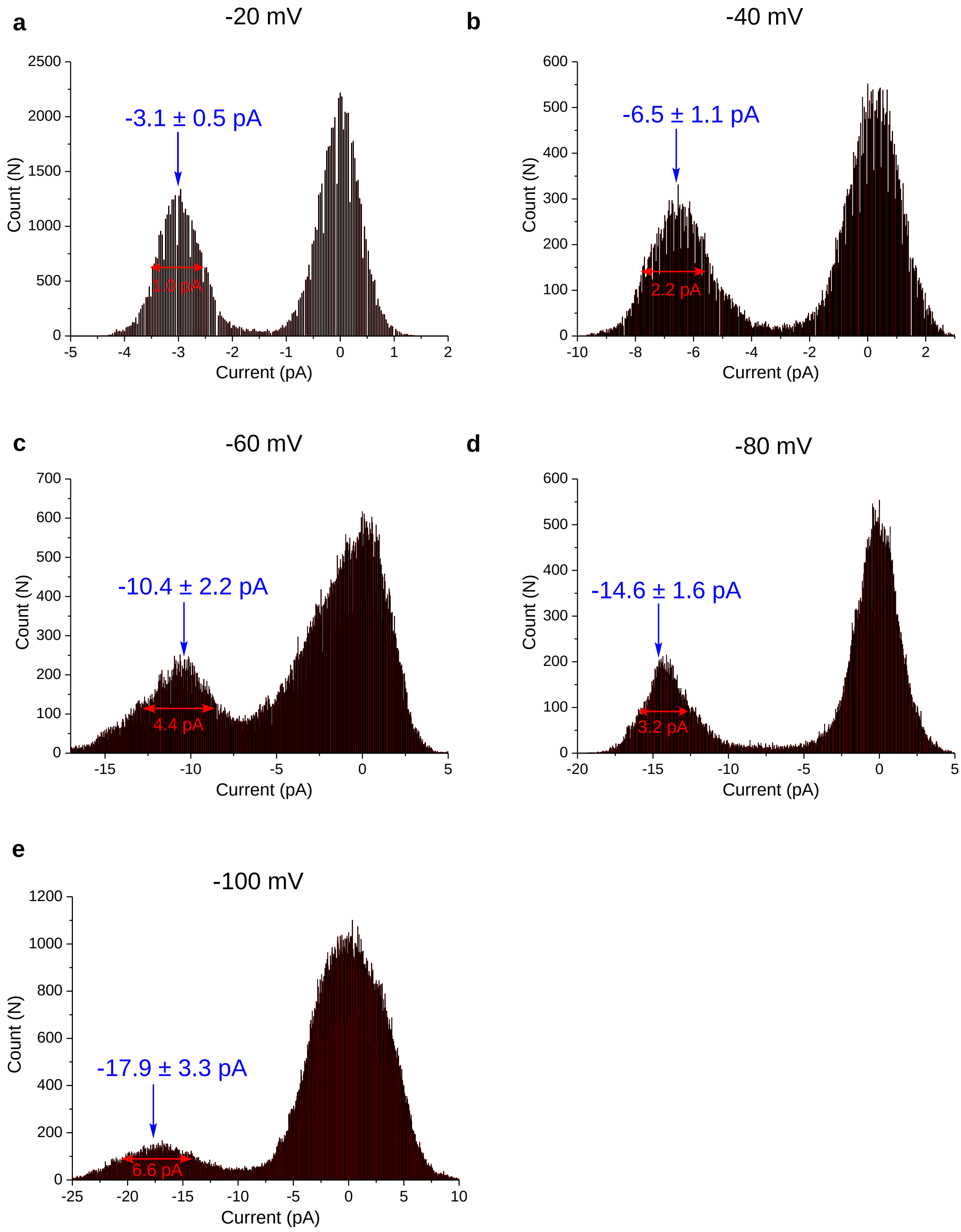

Supplementary Fig. 22 Single channel study of nanopores. Histograms of single channel currents of **Ch-C4** recorded in symmetric baths (*cis* chamber = *trans* chamber = 1 M KCl) at transmembrane potentials of **a** -20, **b** -40, **c** -60, **d** -80 and **e** -100 mv. Relative errors were obtained by dividing the width between the two half height points by 2. Source data are provided as a Source Data file.

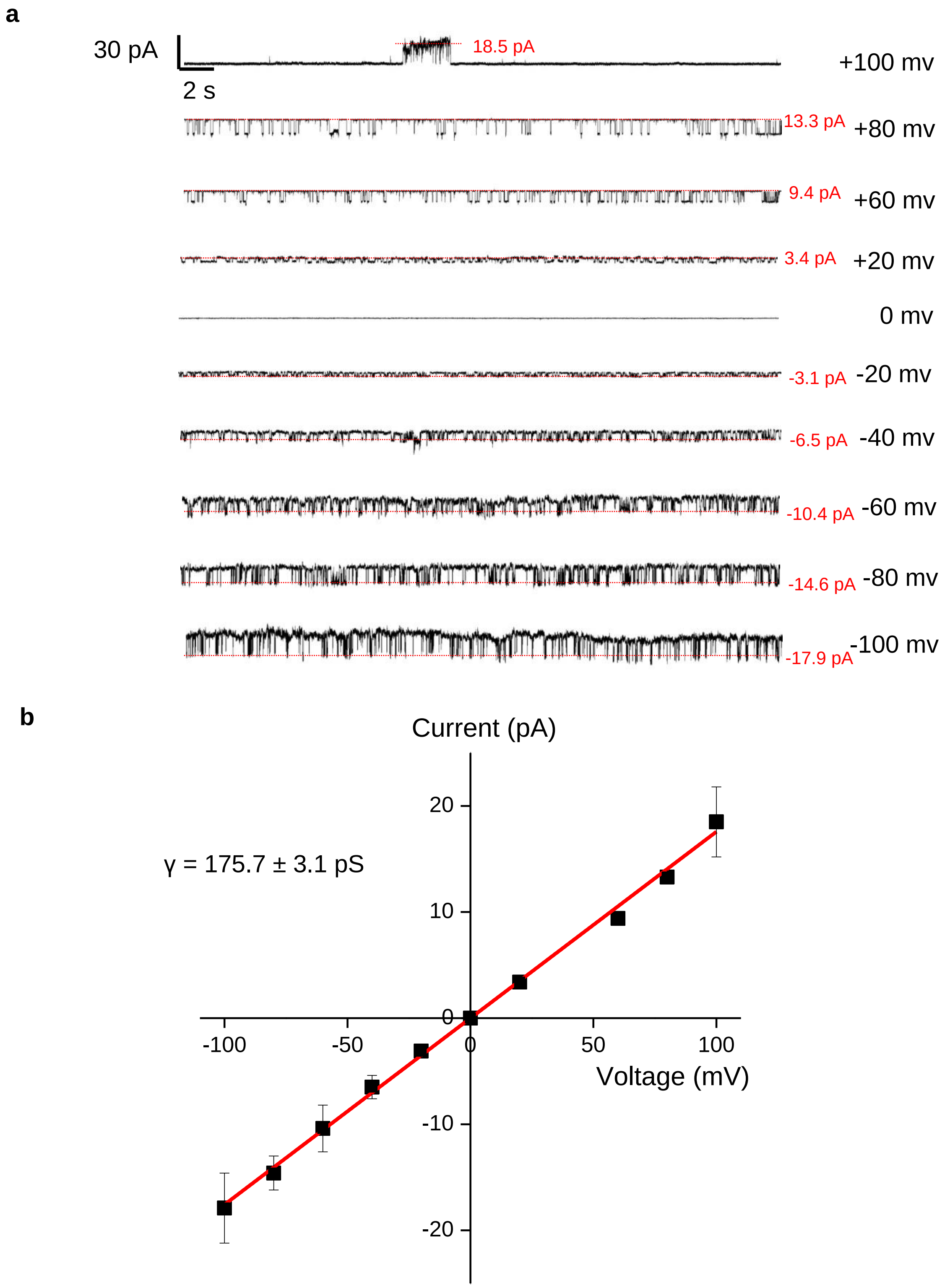

Supplementary Fig. 23 Single channel study of nanopores. a Single channel current traces of **Ch-C4** recorded at various voltages in symmetric baths (*cis* chamber = *trans* chamber = 1 M KCl). Dotted red lines refer to the mean current values that were obtained from histograms and used to obtain the conductance value. **b** Current-voltage (I-V) curve for obtaining the ion conductance (γ) for **Ch-C4**. The pore size was estimated to be 0.90 nm. Data are represented as mean current values ± half width at half maximum. n = 3 independent experiments. Source data are provided as a Source Data file.

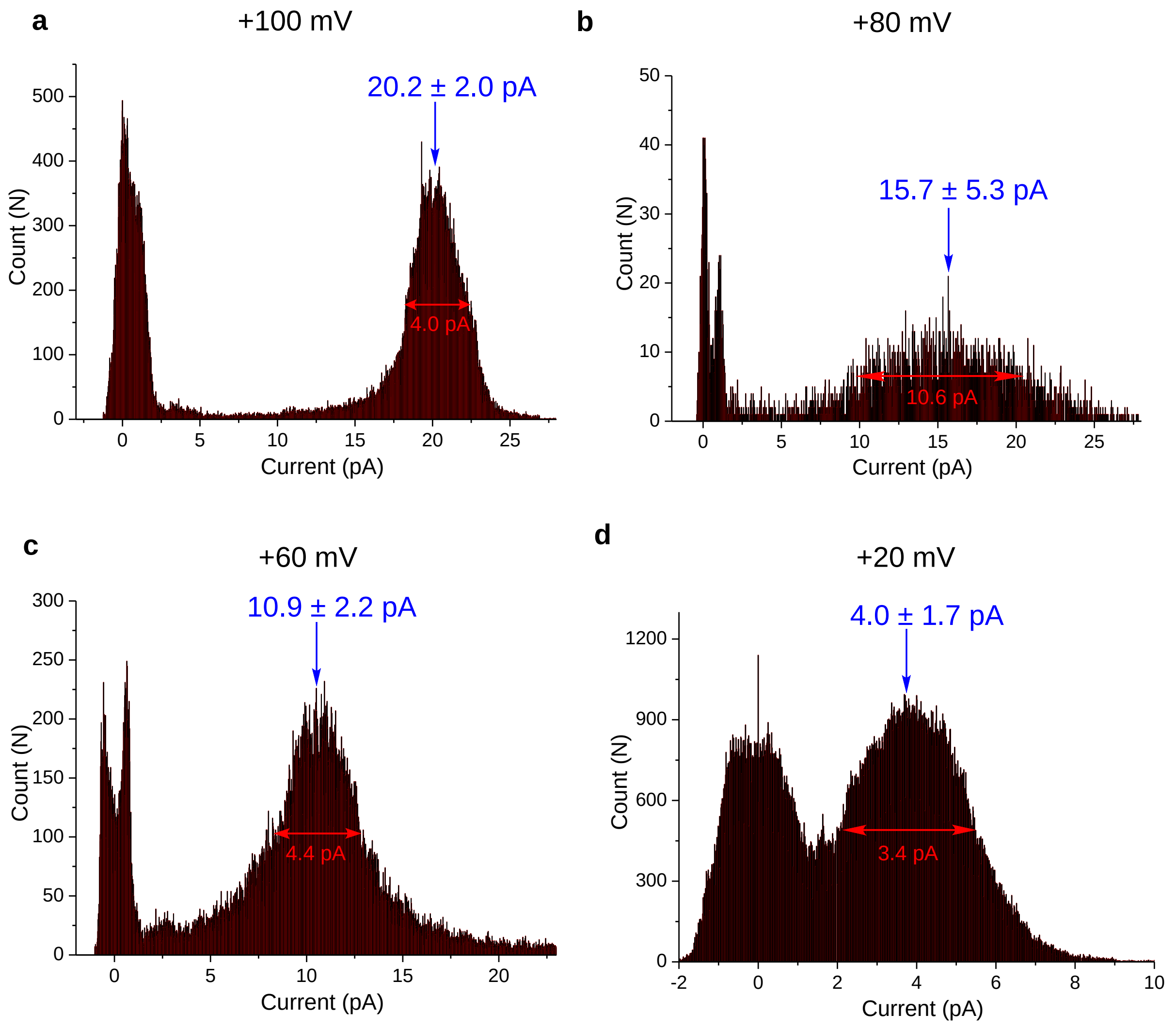

Supplementary Fig. 24 Single channel study of nanopores. Histograms of single channel currents of **Ch-C4** recorded in symmetric baths (*cis* chamber = *trans* chamber = 1 M KCl) at transmembrane potentials of **a** 100, **b** 80, **c** 60 and **d** 20 mv in a repeated assay. Relative errors were obtained by dividing the width between the two half height points by 2. Source data are provided as a Source Data file.

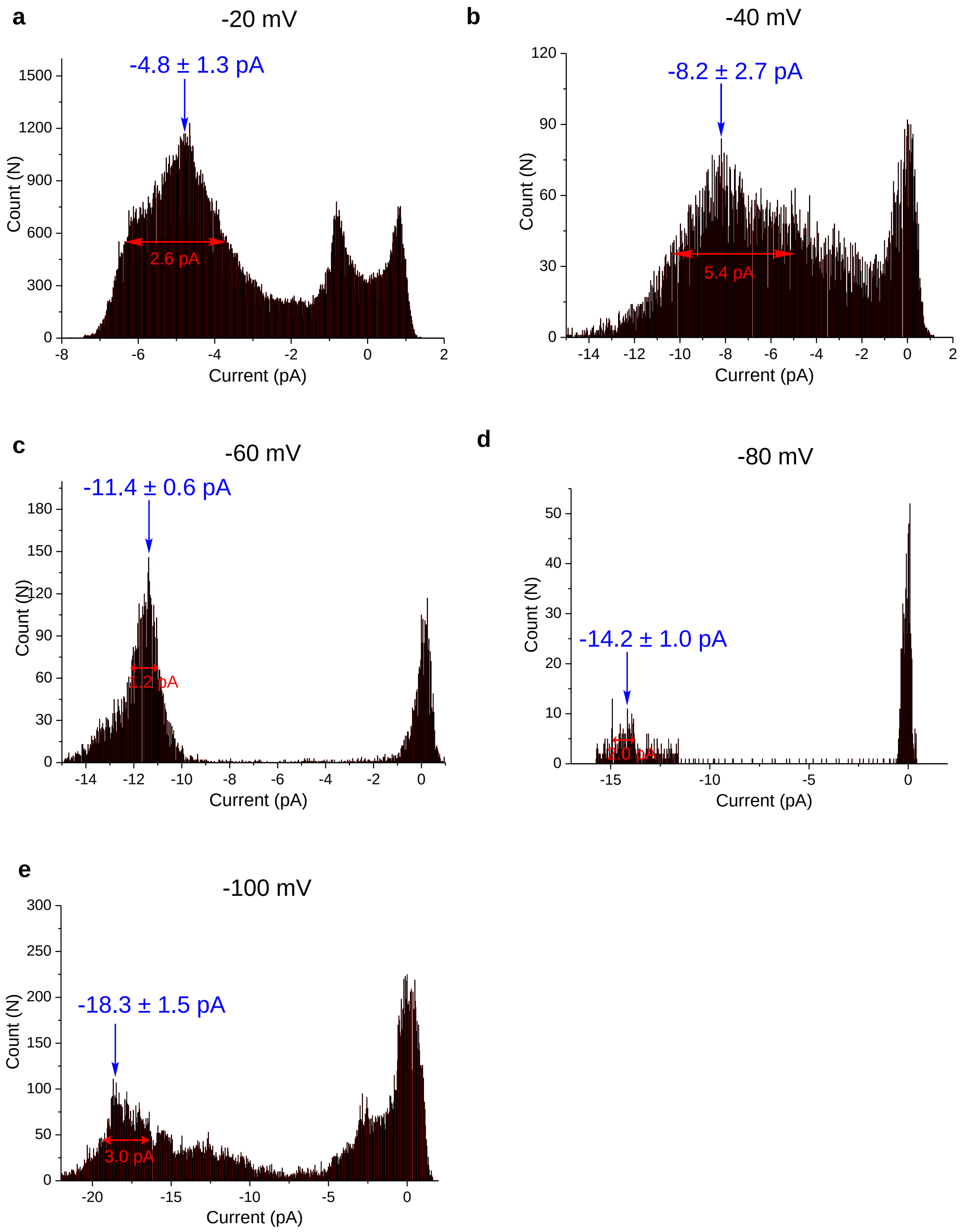

Supplementary Fig. 25 Single channel study of nanopores. Histograms of single channel currents of **Ch-C4** recorded in symmetric baths (*cis* chamber = *trans* chamber = 1 M KCl) at transmembrane potentials of **a** -20, **b** -40, **c** -60, **d** -80 and **e** -100 mv in a repeated assay. Relative errors were obtained by dividing the width between the two half height points by 2. Source data are provided as a Source Data file.

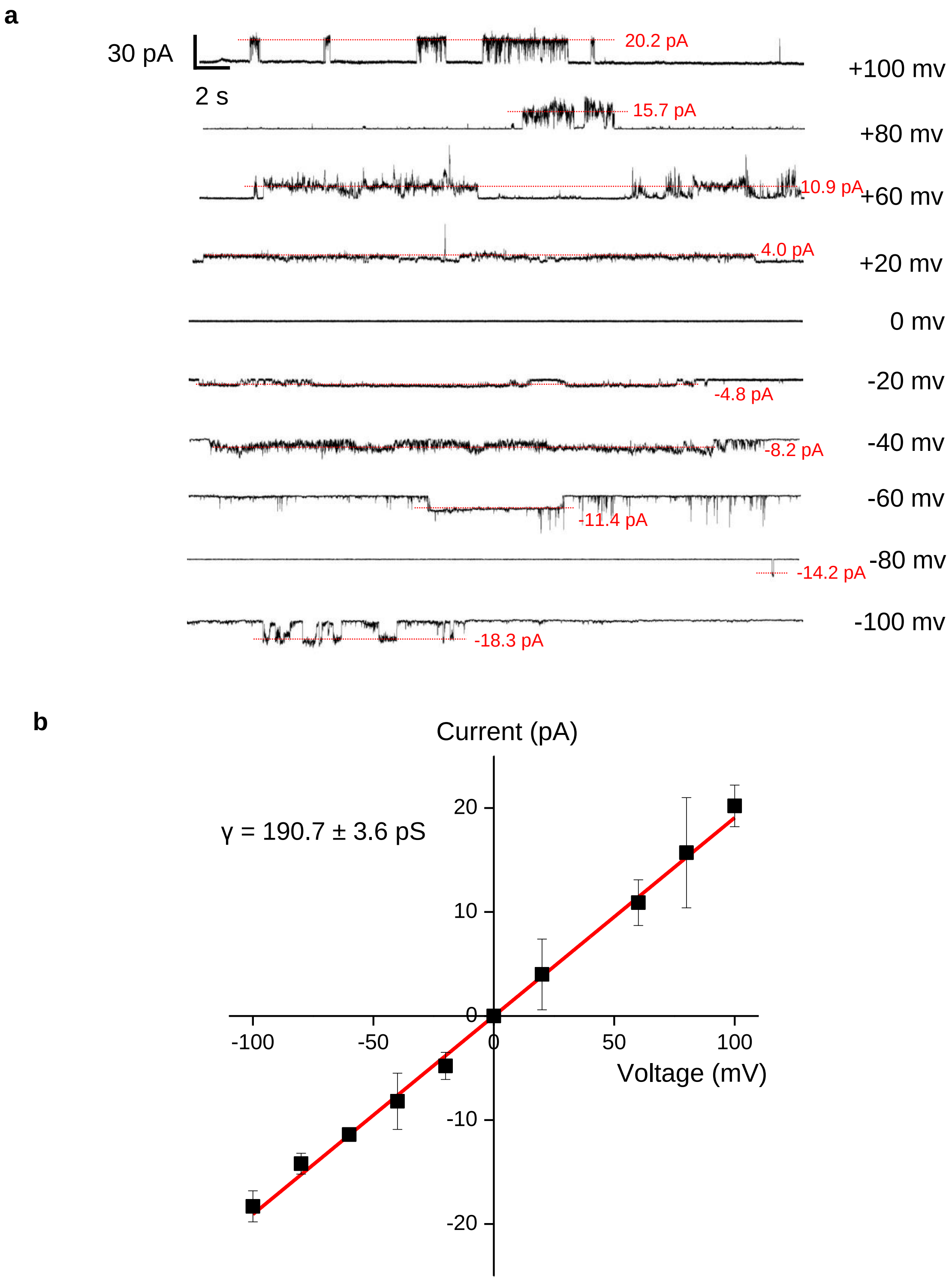

Supplementary Fig. 26 Single channel study of nanopores. a Single channel current traces of **Ch-C4** recorded at various voltages in symmetric baths (*cis* chamber = *trans* chamber = 1 M KCl) in a repeated assay. Dotted red lines refer to the mean current values that were obtained from histograms and used to obtain the conductance value. **b** Current-voltage (I-V) curve for obtaining the ion conductance (γ) for **Ch-C4**. The pore size was estimated to be 0.94 nm. Data are represented as mean current values ± half width at half maximum. n = 3 independent experiments. Source data are provided as a Source Data file.

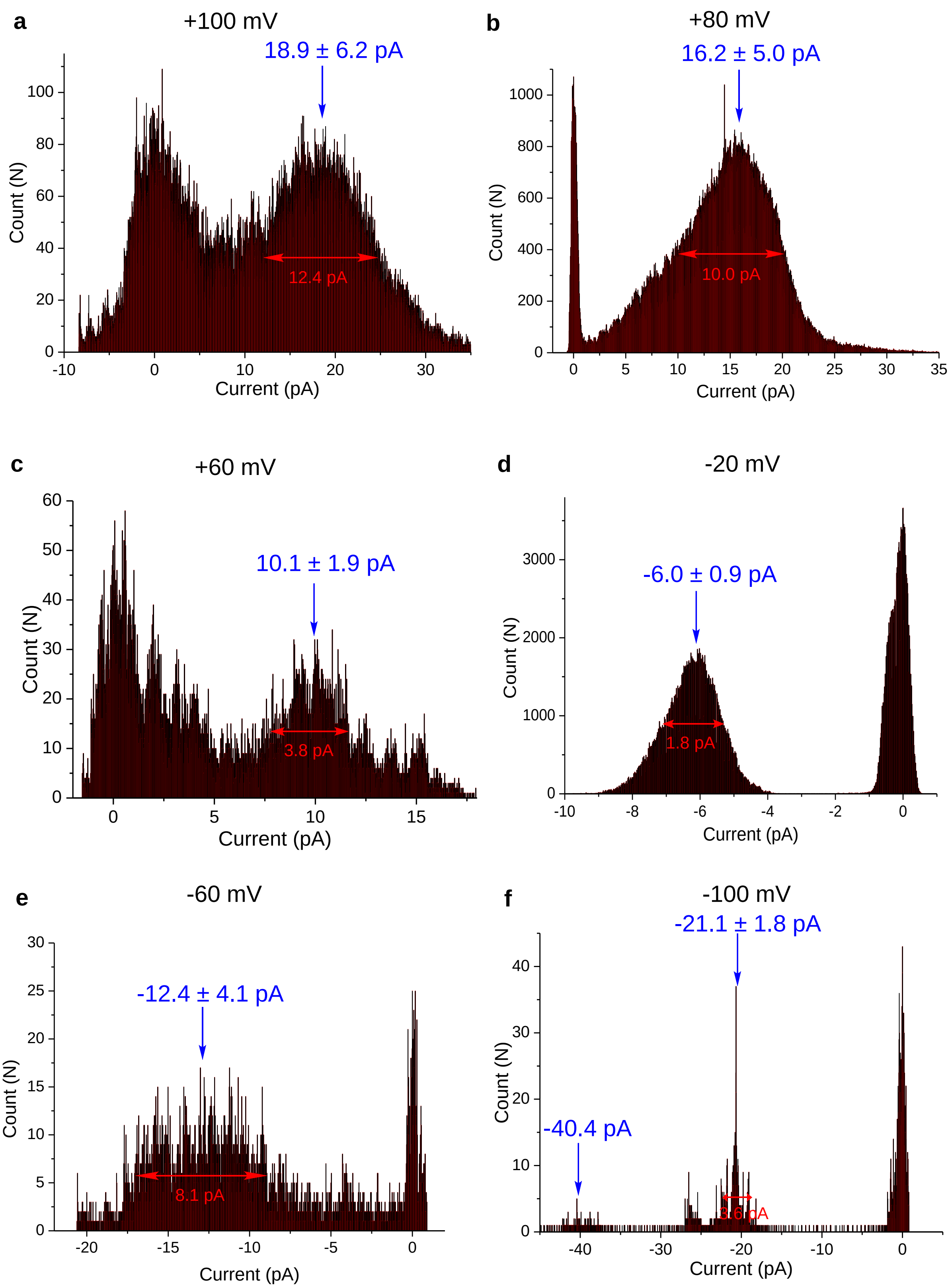

Supplementary Fig. 27 Single channel study of nanopores. Histograms of single channel currents of **Ch-C4** recorded in symmetric baths (*cis* chamber = *trans* chamber = 1 M KCl) at transmembrane potentials of **a** 100, **b** 80, **c** 60, **d** -20, **e** -60 and **f** -100 mv in a repeated assay. Relative errors were obtained by dividing the width between the two half height points by 2. Source data are provided as a Source Data file.

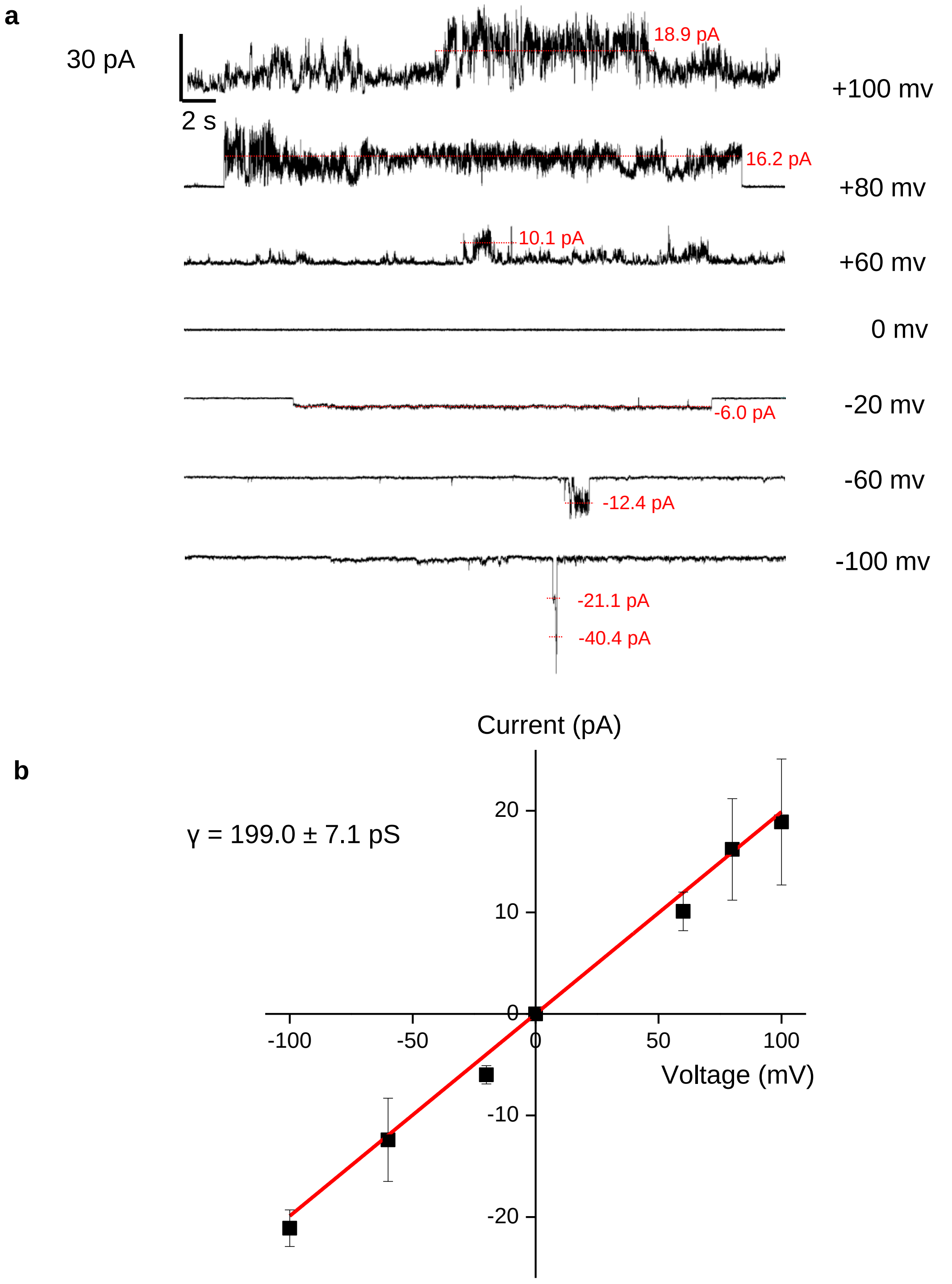

Supplementary Fig. 28 Single channel study of nanopores. a Single channel current traces of **Ch-C4** recorded in symmetric baths (*cis* chamber = *trans* chamber = 1 M KCl) in a repeated assay. Dotted red lines refer to the mean current values that were obtained from histograms and used to obtain the conductance value. **b** Current-voltage (I-V) curve for obtaining the ion conductance (γ) for **Ch-C4**. The pore size was estimated to be 0.98 nm. Data are represented as mean current values ± half width at half maximum. n = 3 independent experiments. Source data are provided as a Source Data file.

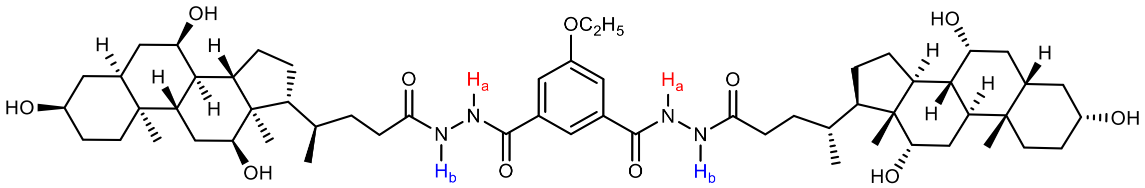

Supplementary Fig. 29 ^{1}H NMR spectra characterization of channel molecules upon addition of

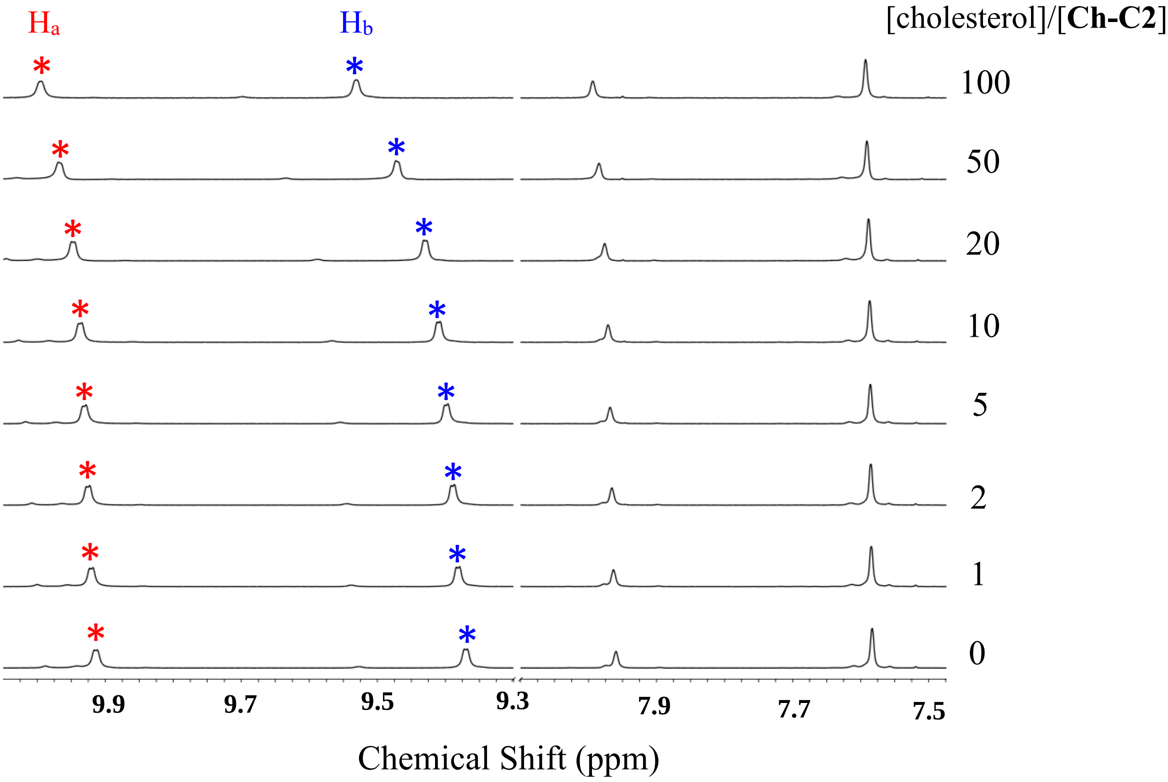

cholesterol. ^{1}H NMR titration experiments involving titrating 0 - 100 equivalents of cholesterol into THF-d_8 containing **Ch-C2** at 5 mM. The overall changes of 0.08 ppm and 0.16 ppm for H_a and H_b were observed, respectively.

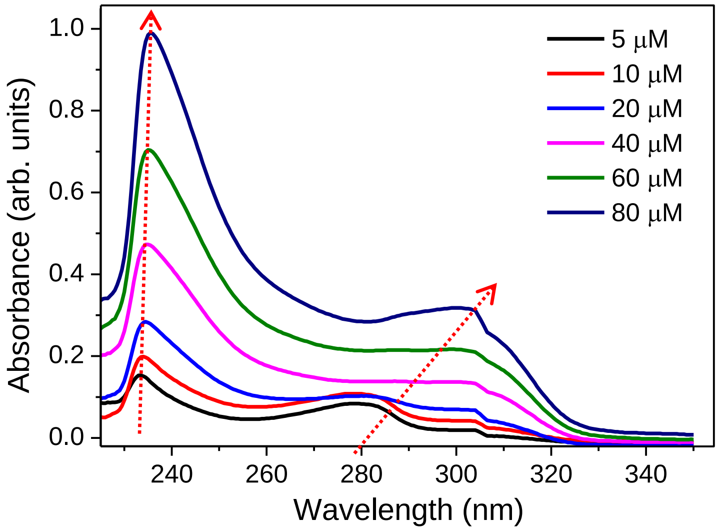

Supplementary Fig. 30 UV-vis spectra characterization of channel molecules. UV-vis absorption spectra of **Ch-C2** in THF at various concentrations at 20 °C. Increasing the concentration of **Ch-C2** from 5 μM to 80 μM resulted in overall red shifts of 2.5 and 21 nm for maximum and minor absorption peaks, respectively. Source data are provided as a Source Data file.

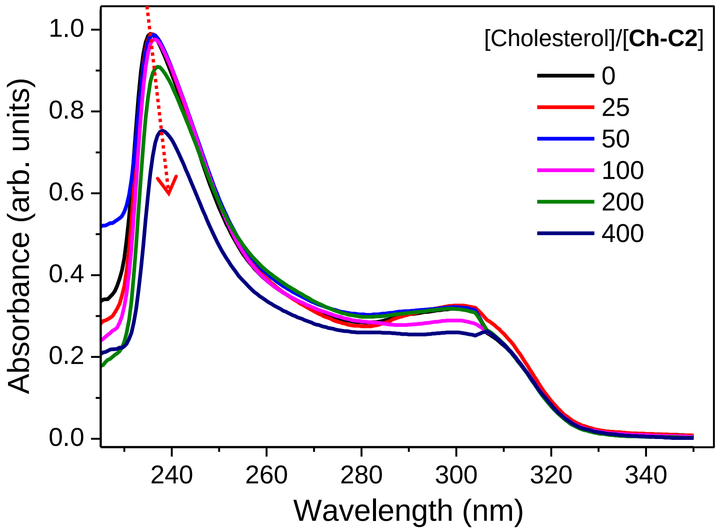

Supplementary Fig. 31 UV-vis spectra characterization of channel molecules upon addition of cholesterol. UV-vis absorption spectra of **Ch-C2** (80 μM) in THF in the presence of 0 - 400 equivalents of cholesterol at 20 °C. An overall red shift in maximum absorption wavelength (λ_{abs}) by 2.5 nm and a 24% decrease in maximum absorption intensity were observed. The background signals of the THF solutions containing the same concentration of cholesterol have been subtracted. Source data are provided as a Source Data file.

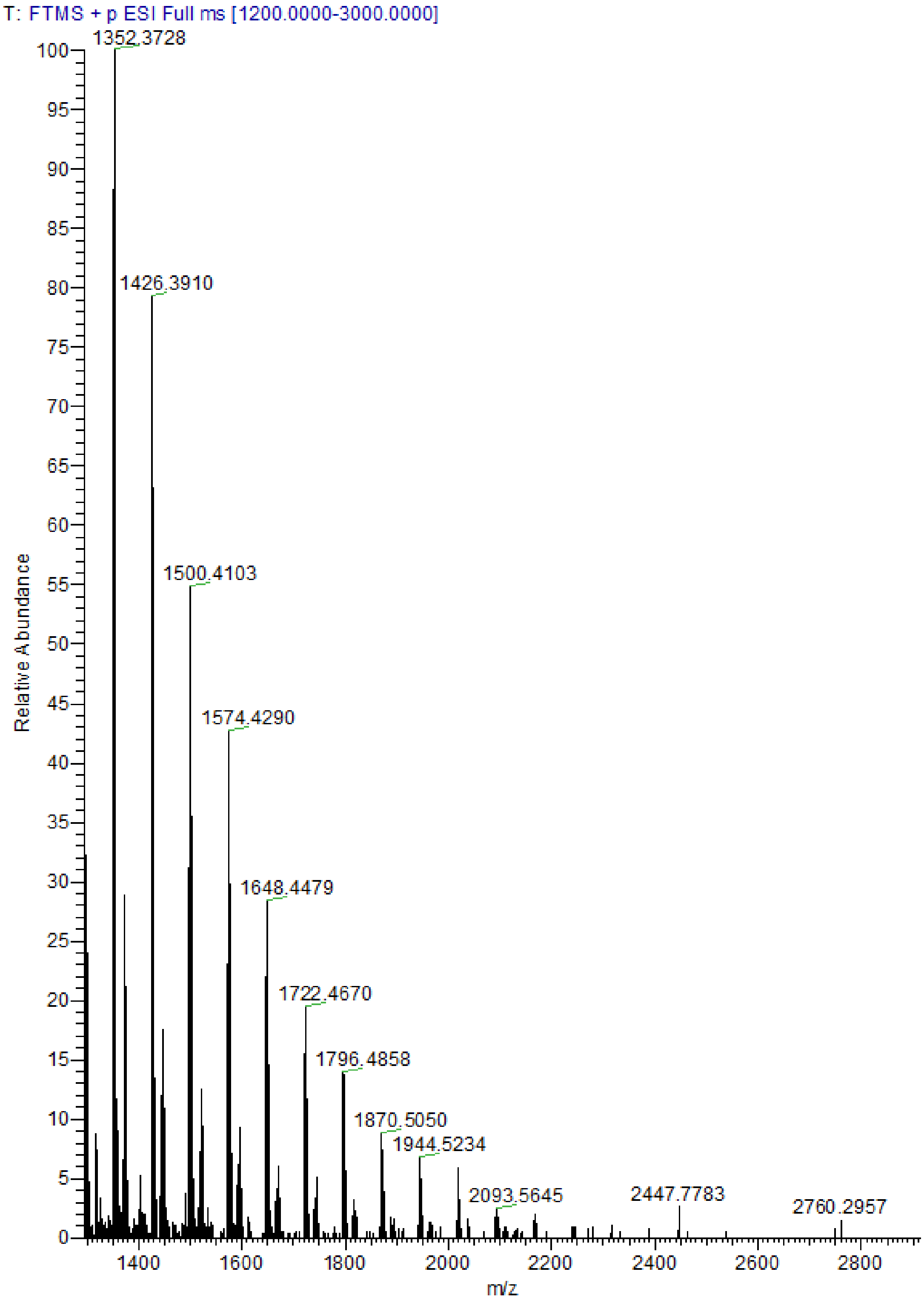

Supplementary Fig. 32 HRMS spectra characterization of channel self-assembly. High-resolution mass spectrum of a blank solution containing no channel molecules.

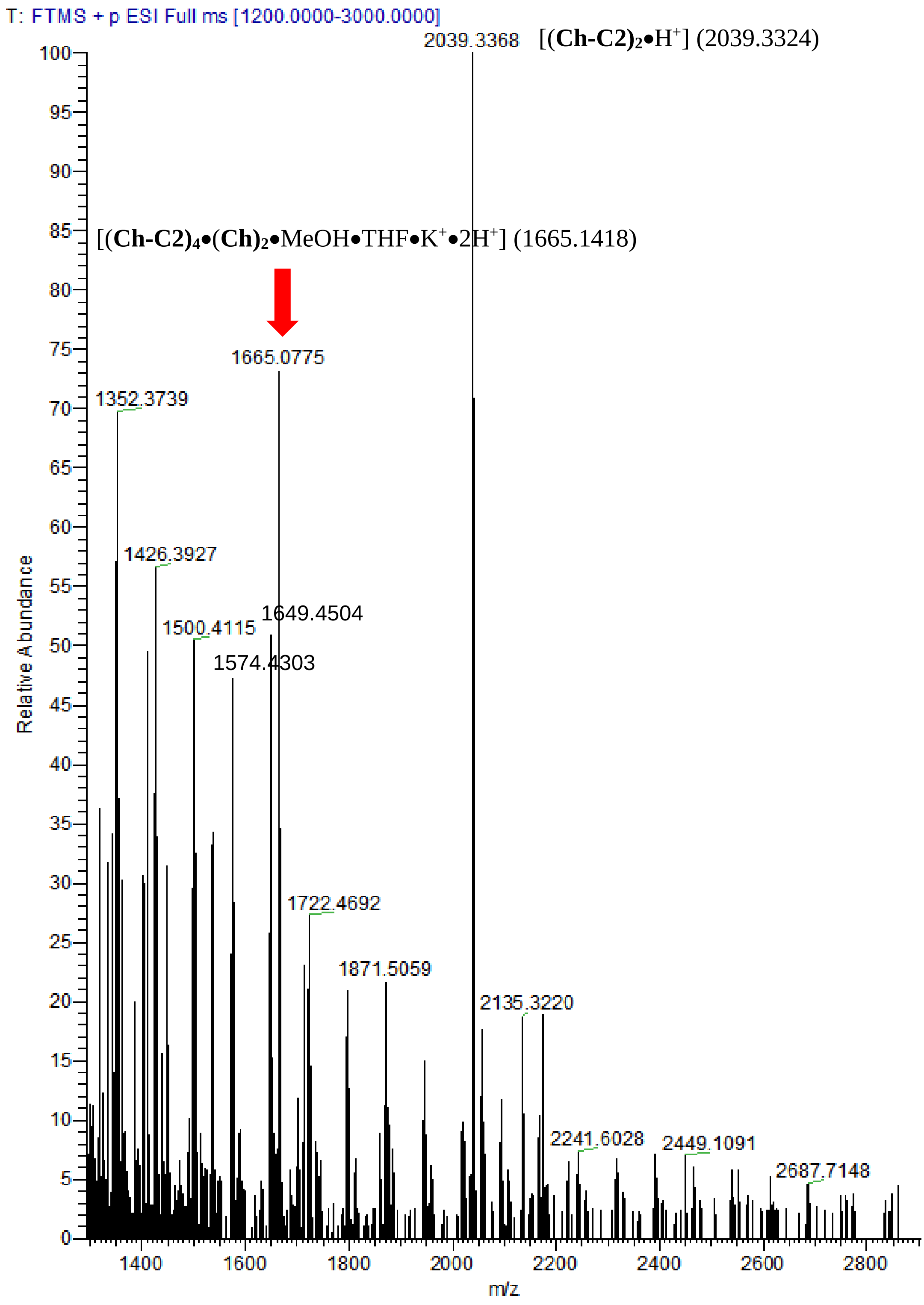

Supplementary Fig. 33 HRMS spectra characterization of channel self-assembly. High-resolution mass spectrum of **Ch-C2** (10 μM) in the presence of 100 equivalents of cholesterol (1 mM). ([(**Ch-C2**)$_2$•H]$^+_{cal.}$ = 2039.3324, [(**Ch-C2**)$_2$•H]$^+_{found}$ = 2039.3368; [(**Ch-C2**)$_4$•(**Ch**)$_2$•MeOH•THF•K•H$_2$]$^{3+}_{cal.}$ = 1665.1418, [(**Ch-C2**)$_4$•(**Ch**)$_2$•MeOH•THF•K•H$_2$]$^{3+}_{found}$ = 1665.0775)

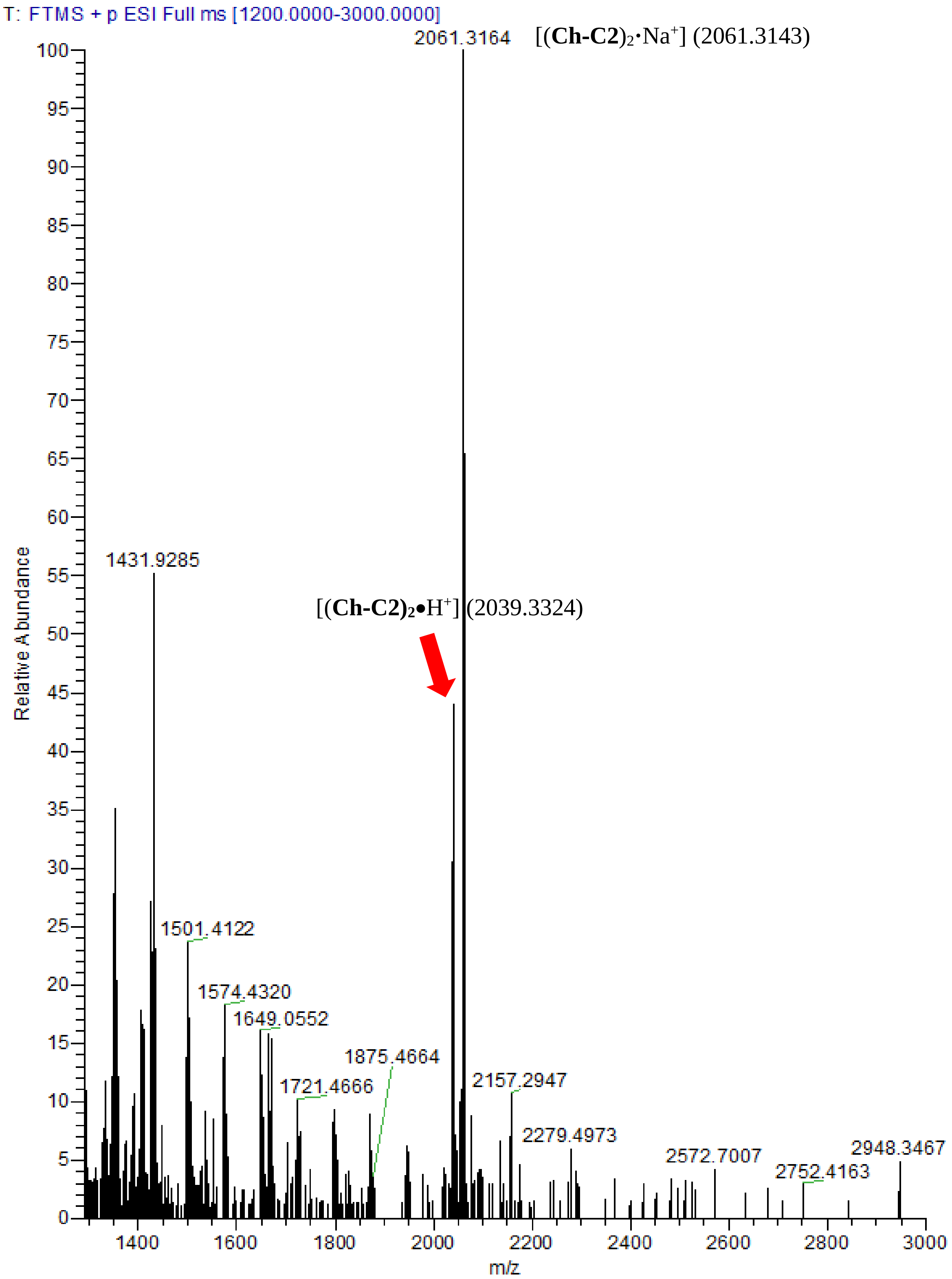

Supplementary Fig. 34 HRMS spectra characterization of channel self-assembly. High-resolution mass spectrum of **Ch-C2** (10 μM) in the presence of 100 equivalents of cholesterol (1 mM). ([2**Ch-C2**•Na]$^{+}_{calculated}$ = 2061.3143, [2**Ch-C2**•Na]$^{+}_{found}$ = 2061.3164)

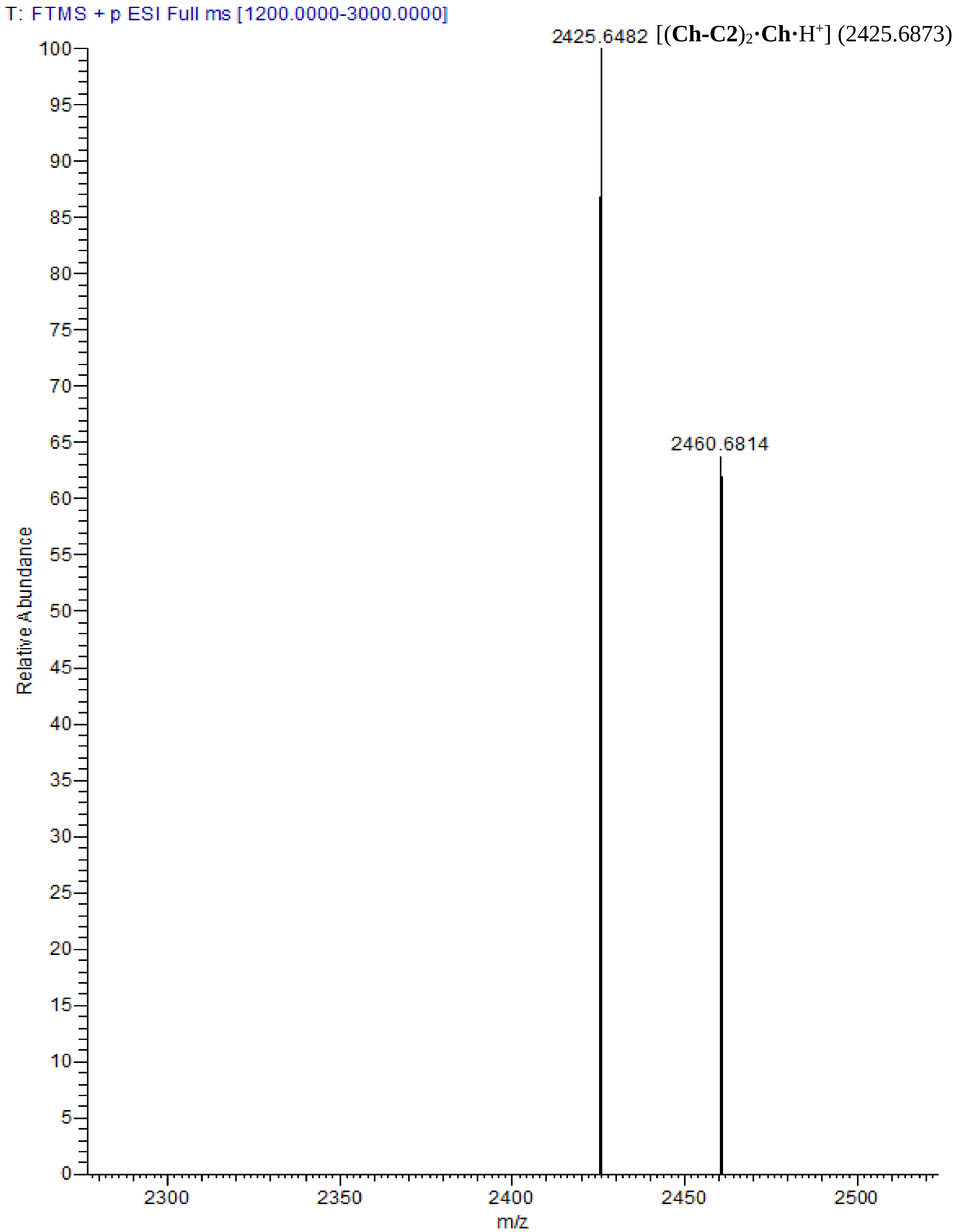

Supplementary Fig. 35 HRMS spectra characterization of channel self-assembly. The identified high-resolution mass spectrum of [2**Ch-C2**•**Ch**•H]$^+$. ([2**Ch-C2**•**Ch**•H]$^+_{cal.}$ = 2425.6873, [2**Ch-C2**•**Ch**•H]$^+_{found}$ = 2425.6482)

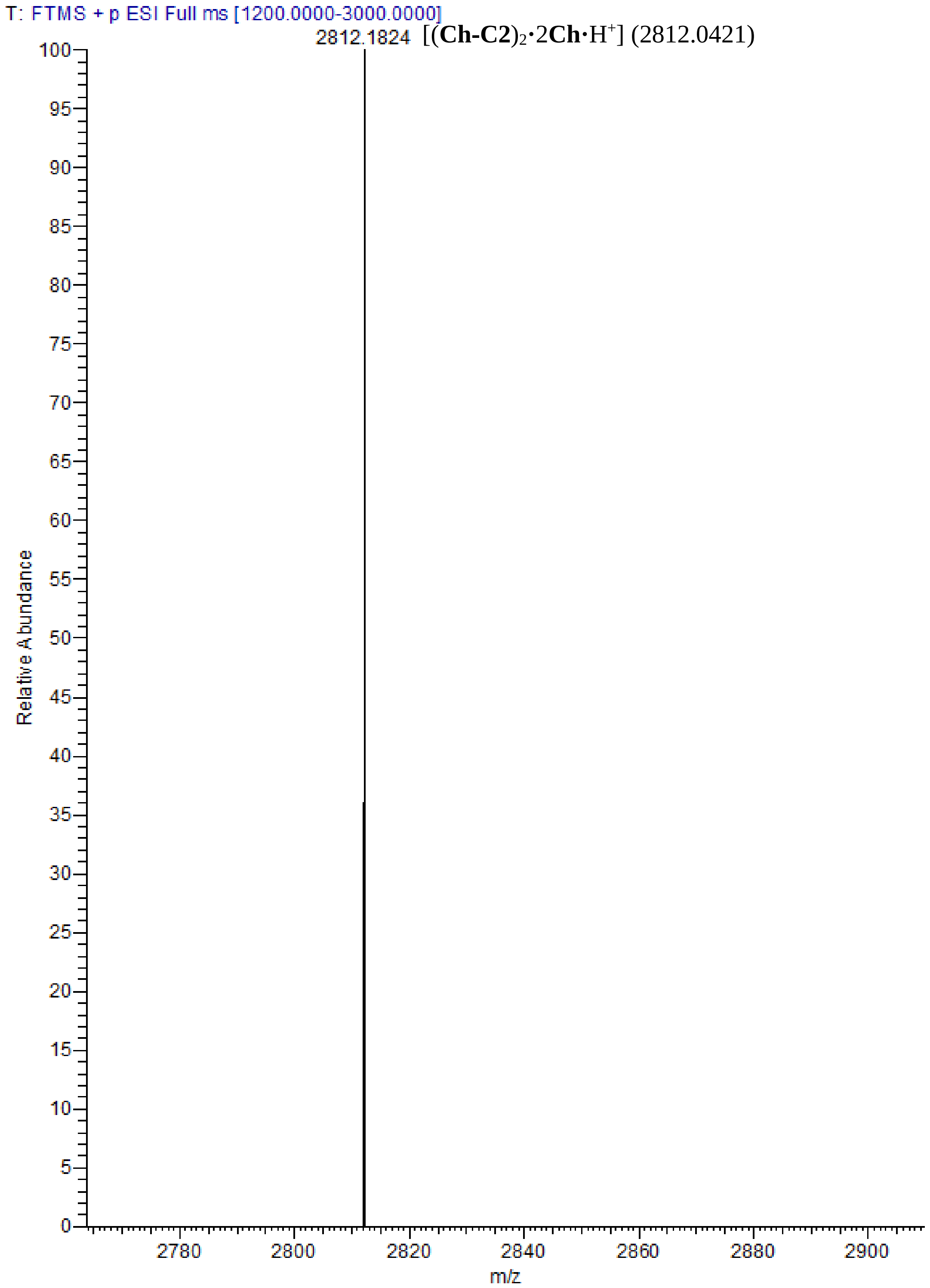

Supplementary Fig. 36 HRMS spectra characterization of channel self-assembly. The identified high-resolution mass spectrum of [2**Ch-C2**•2**Ch**•H]$^+$. ([2**Ch-C2**•2**Ch**•H]$^+_{cal.}$ = 2812.0421, [2**Ch-C2**•2**Ch**•H]$^+_{found}$ = 2812.1824)

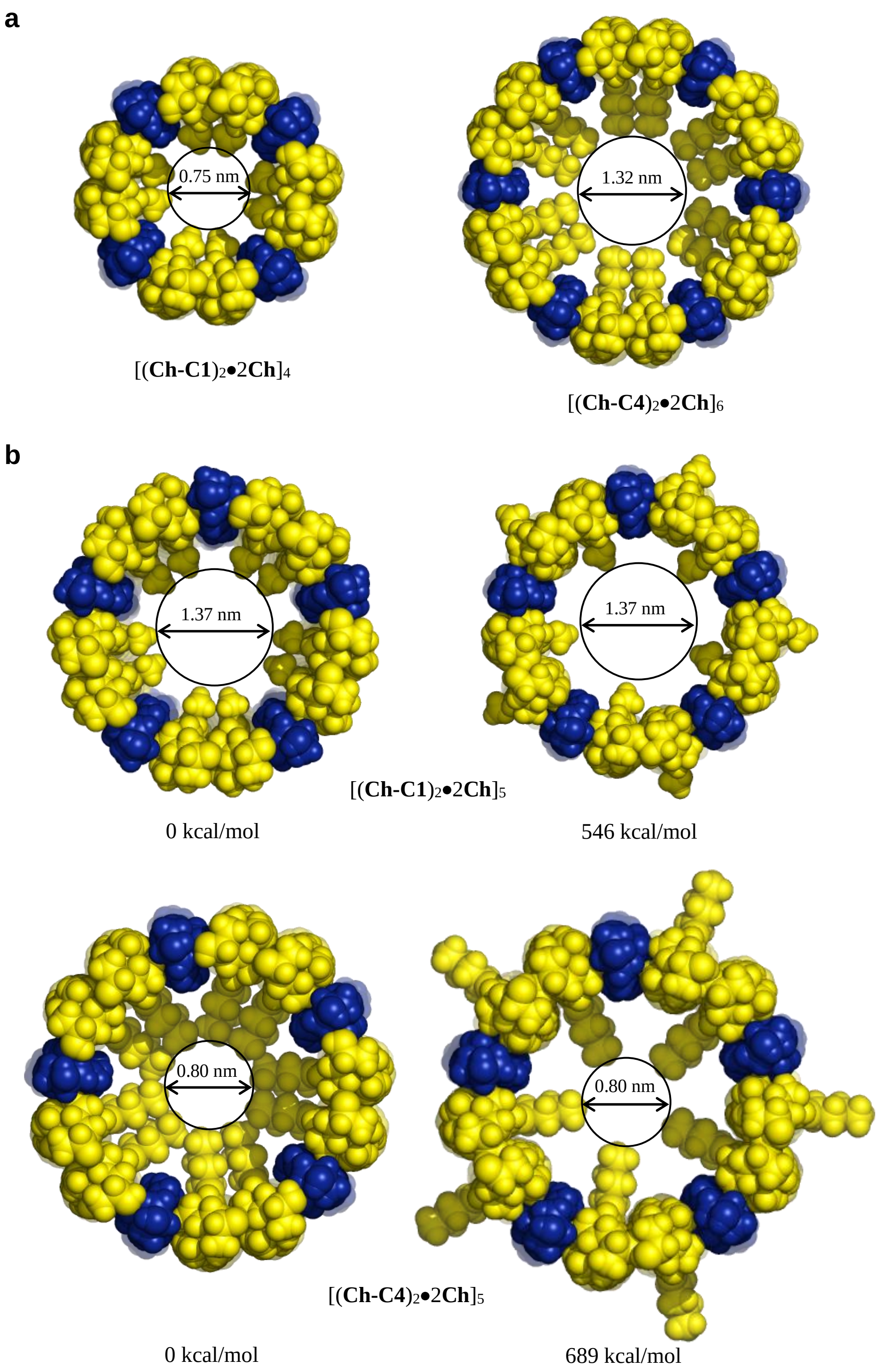

Supplementary Fig. 37 Molecular Dynamic Simulation-derived structures and relative energies. **a** Square- or hexagon-shaped ensembles give a pore size that is either too small or too large with respect to the experimentally determined pore sizes. **b** Ensembles with all side chains pointing inward are energetically more stable than those with half of side chains pointing outward.

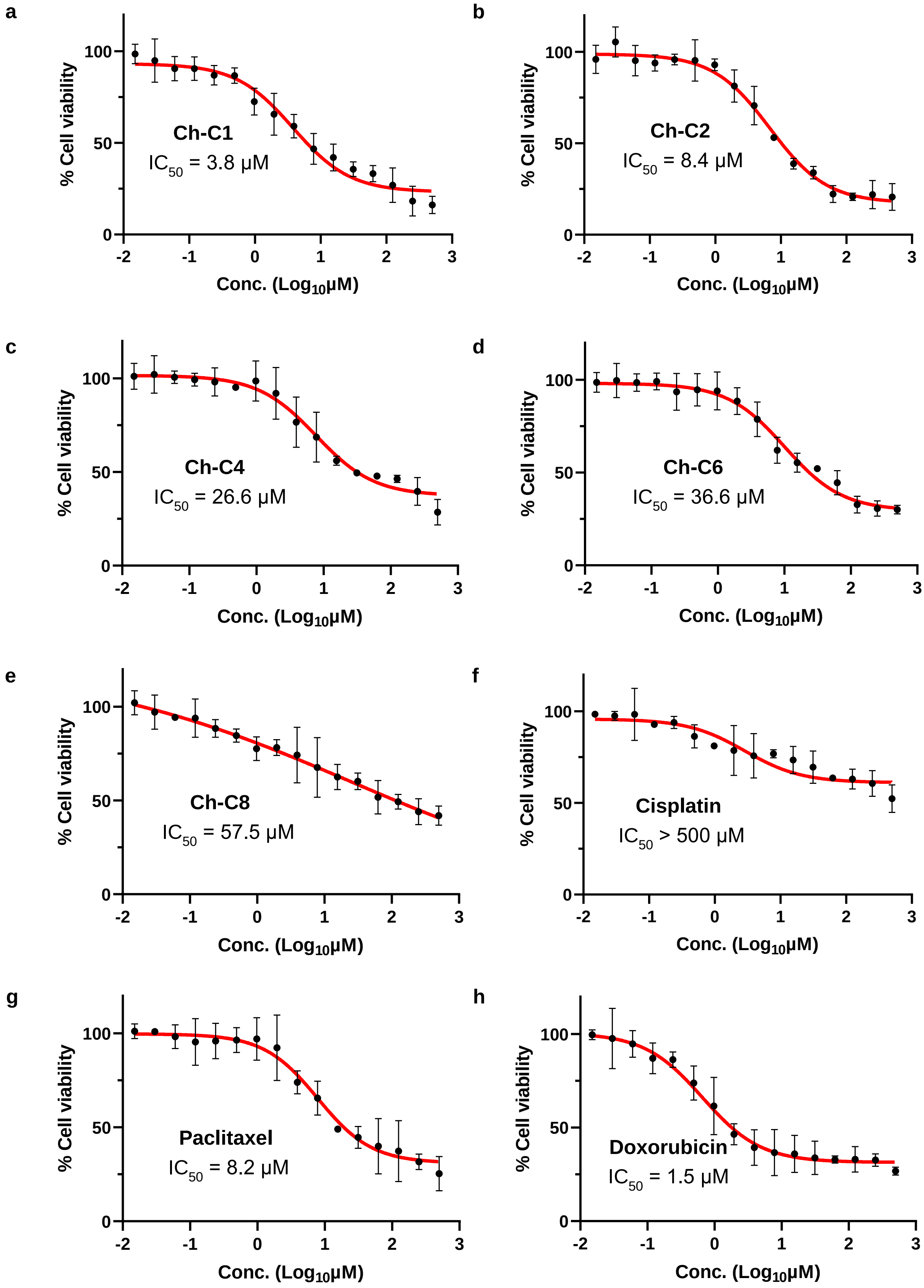

Supplementary Fig. 38 In vitro anticancer of nanopores. Viabilities of human hepatocellular carcinomas (HepG2) cells in the presence of various concentrations of **a Ch-C1**, **b Ch-C2**, **c Ch-C4**, **d Ch-C6**, **e Ch-C8**, **f** Cisplatin, **g** Paclitaxel and **h** Doxorubicin. Data are represented as mean values ± SD. n = 4 biologically independent experiments. Source data are provided as a Source Data file.

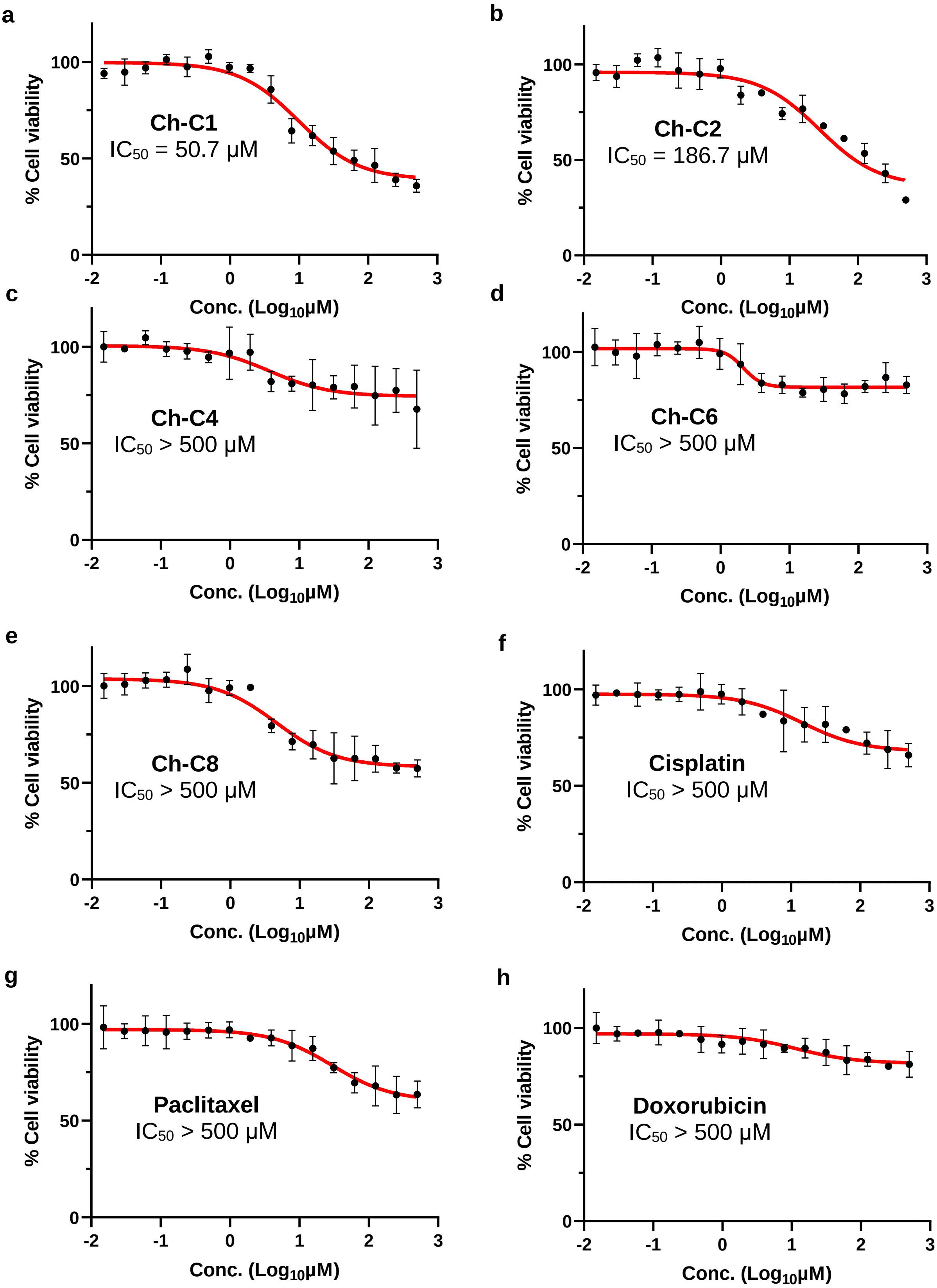

Supplementary Fig. 39 In vitro anticancer of nanopores. Viabilities of human primary glioblastoma (U87-MG) cells in the presence of various concentrations of **a Ch-C1**, **b Ch-C2**, **c Ch-C4**, **d Ch-C6**, **e Ch-C8**, **f** Cisplatin, **g** Paclitaxel and **h** Doxorubicin. Data are represented as mean values ± SD. n = 4 biologically independent experiments. Source data are provided as a Source Data file.

a

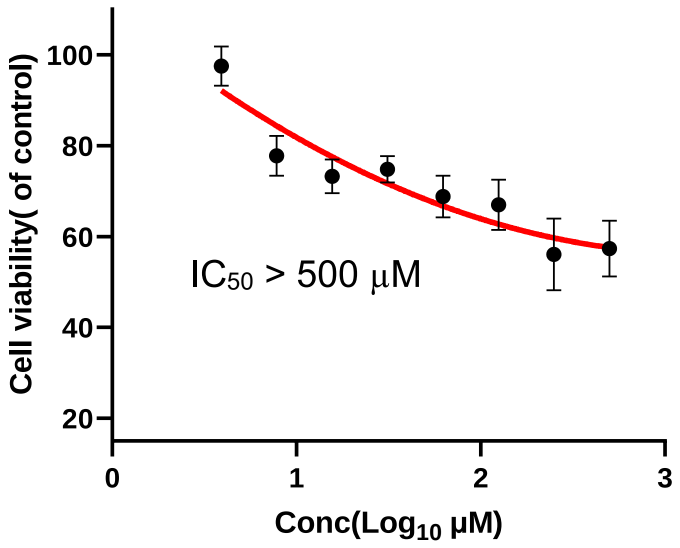

b

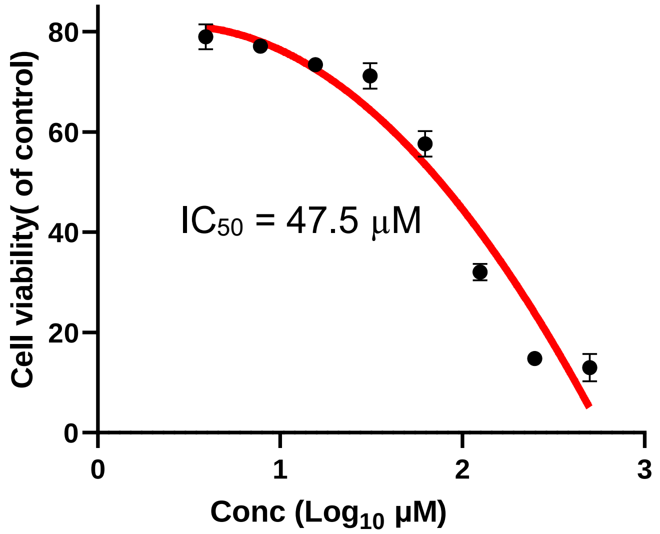

Supplementary Fig. 40 In vitro cytotoxicity of against normal cells. Viabilities of **a** human renal proximal tubular epithelial cells (HK-2) and **b** human normal liver cells (THLE-2) in the presence of various concentrations of **Ch-C1**. Data are represented as mean values ± SD. n = 4 biologically independent experiments. Source data are provided as a Source Data file.

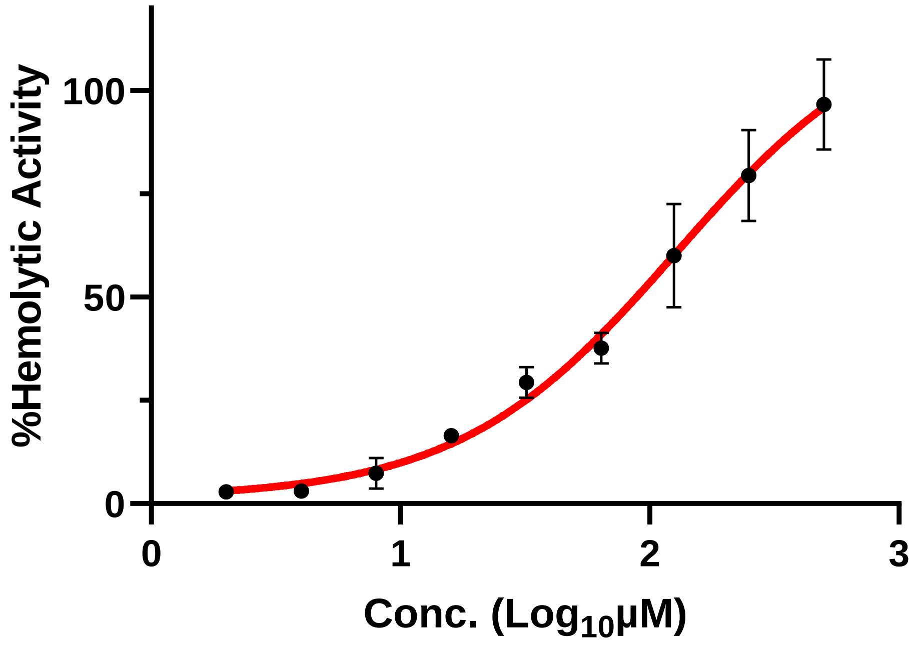

Supplementary Fig. 41 In vitro hemolytic activity of nanopores. Hemolysis of red blood cells in the presence of **Ch-C1** at various concentrations. Data are represented as mean values ± SD. n = 4 biologically independent experiments. Source data are provided as a Source Data file.

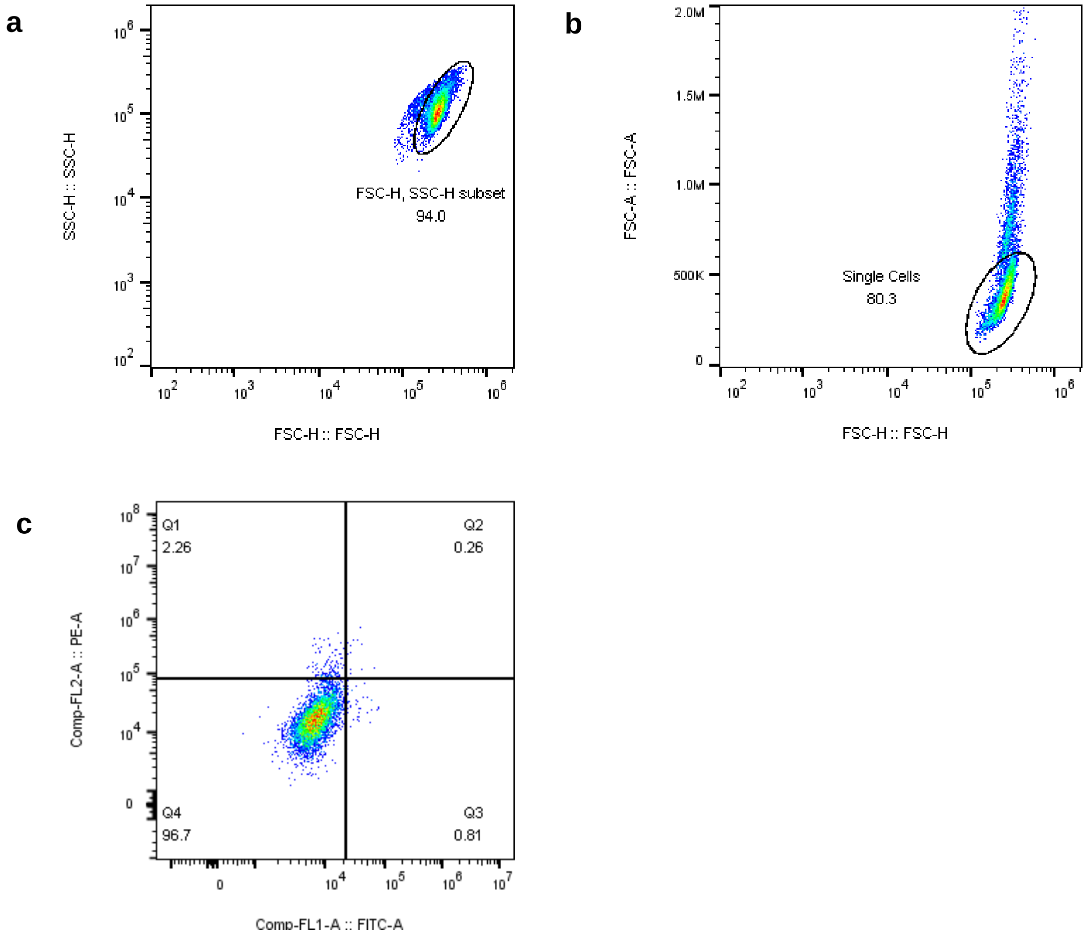

Supplementary Fig. 42 Example of gating strategy for apoptotic cell analysis performed on HepG2 cells. a Target cells were selected from a forward scatter-height vs side scatter-height dot plot, and amounts of cell debris were removed. **b** Single cells were subsequently selected in a forward scatter-area vs forward scatter height dot plot to eliminate the interference of adherent cells. **c** A quad gate is defined with a negative confidence zone based on the control group. According to the trend of cell population, the demarcation line of the tetrad can be set at the colony of negative and positive cells. As normal cultured cells also undergo natural apoptosis, this serves as a negative control of non-specific fluorescence signal (false positive), which is generally less than 3%.

^{1}H NMR and ^{13}C NMR Spectra

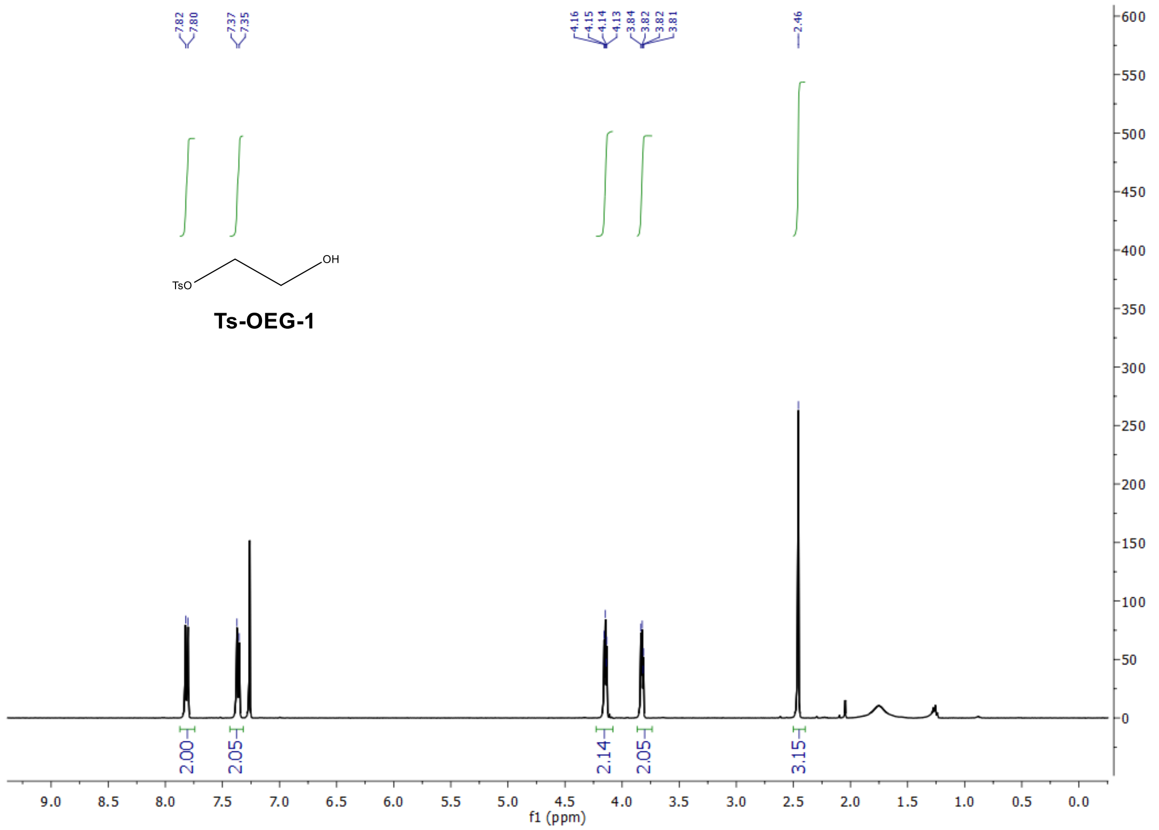

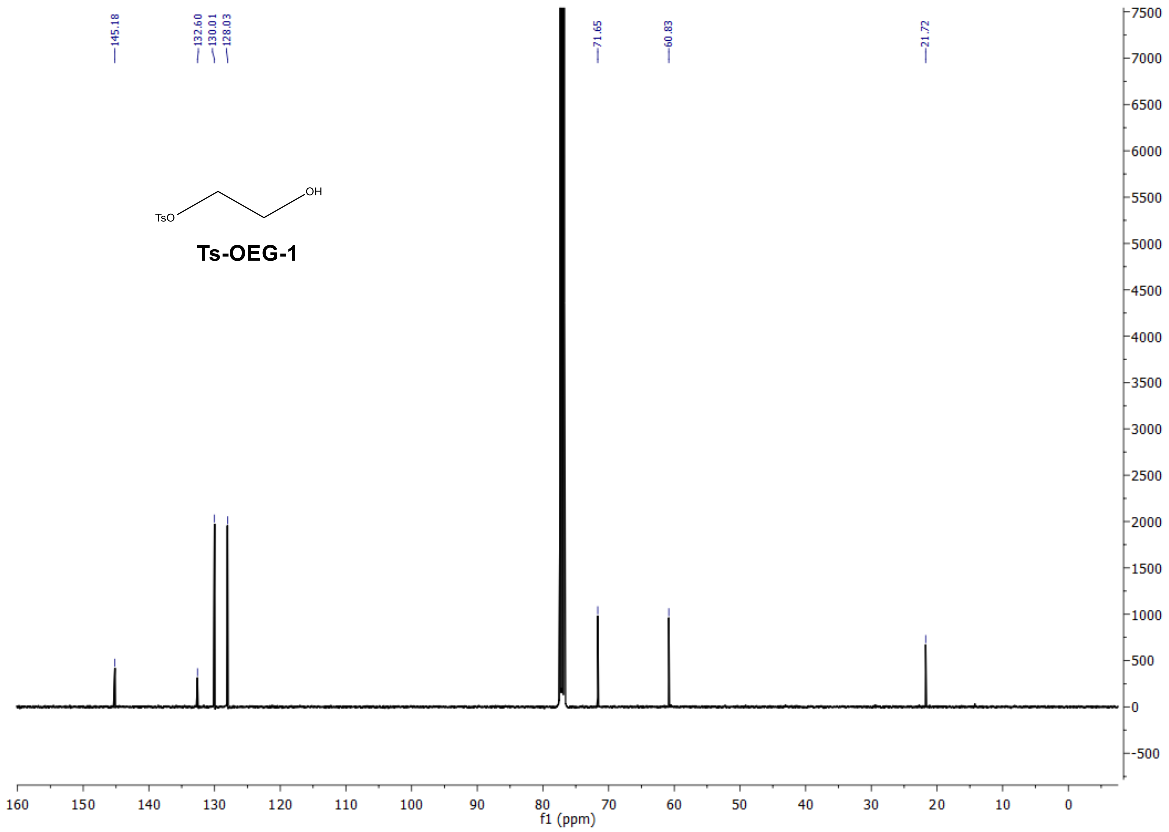

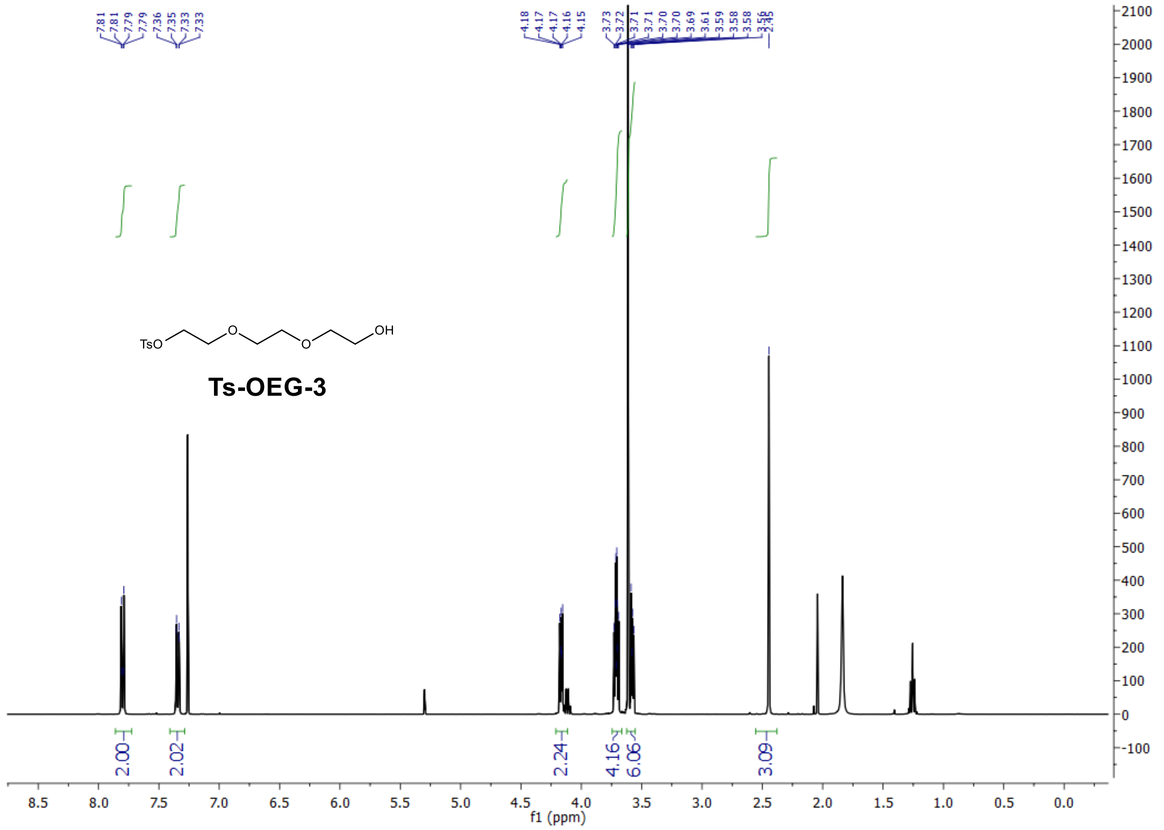
TsO
O
O
OH
Ts-OEG-3
f1 (ppm)

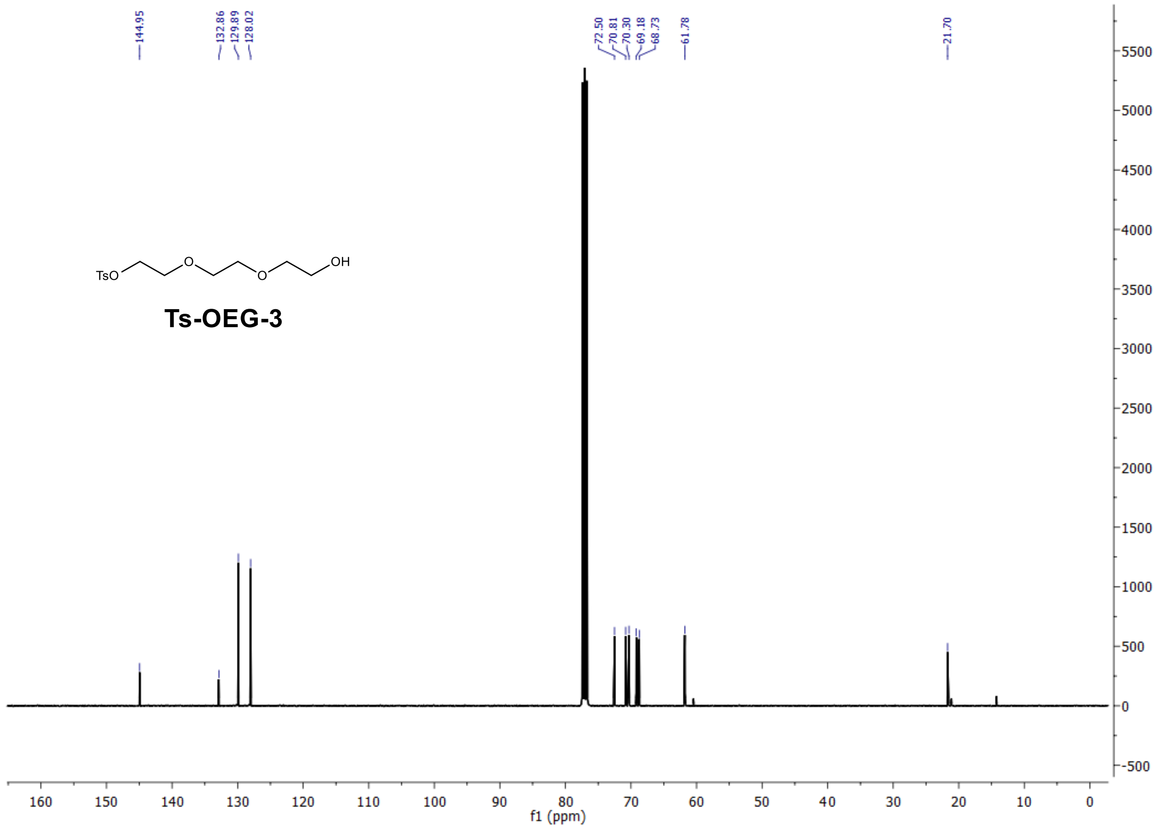
TsO
O
O
OH
Ts-OEG-3
f1 (ppm)

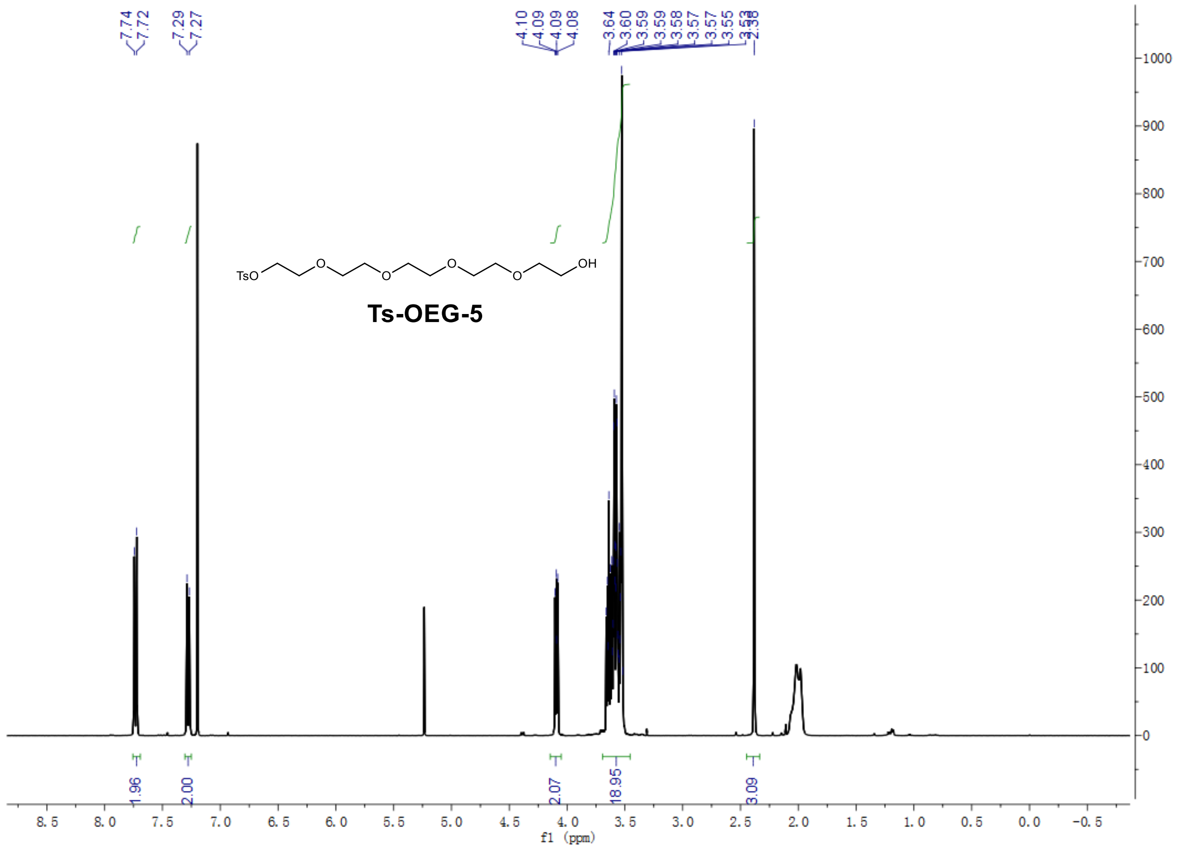
Ts-OEG-5
f1 (ppm)

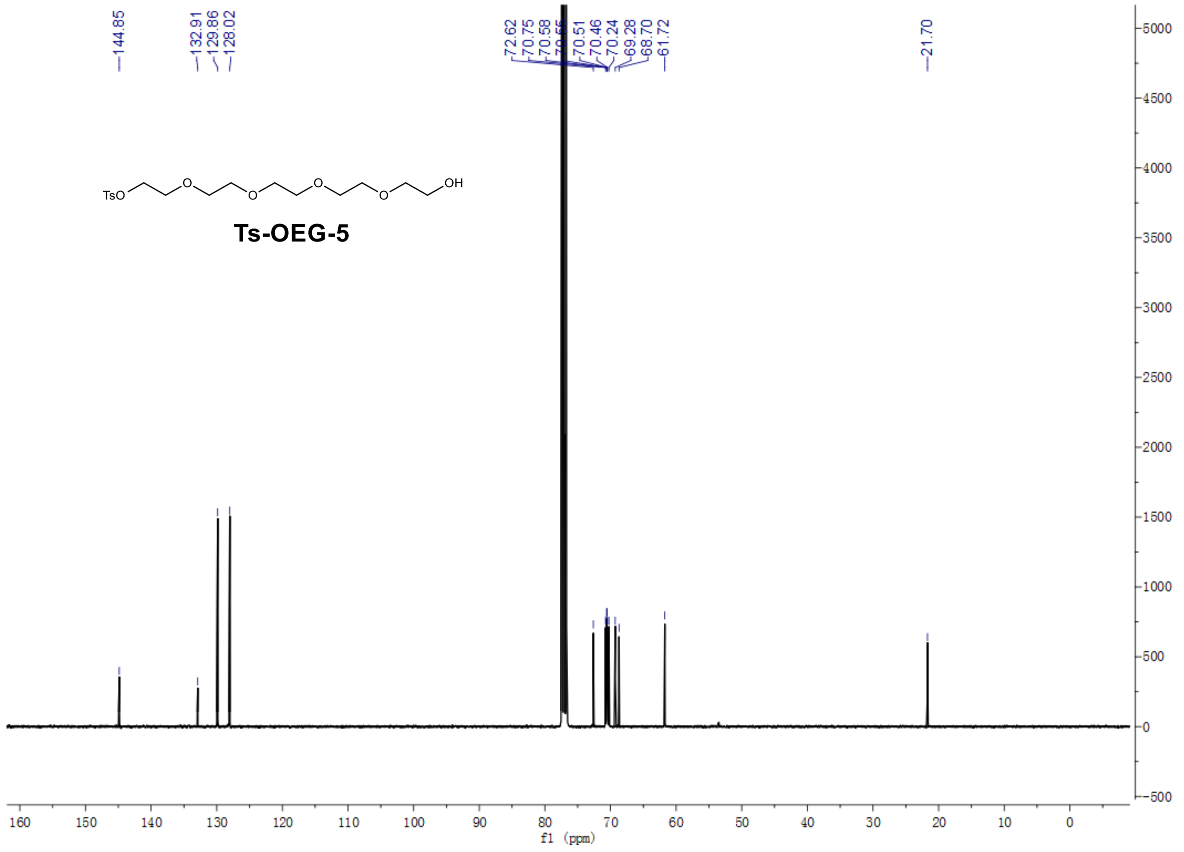
Ts-OEG-5
f1 (ppm)

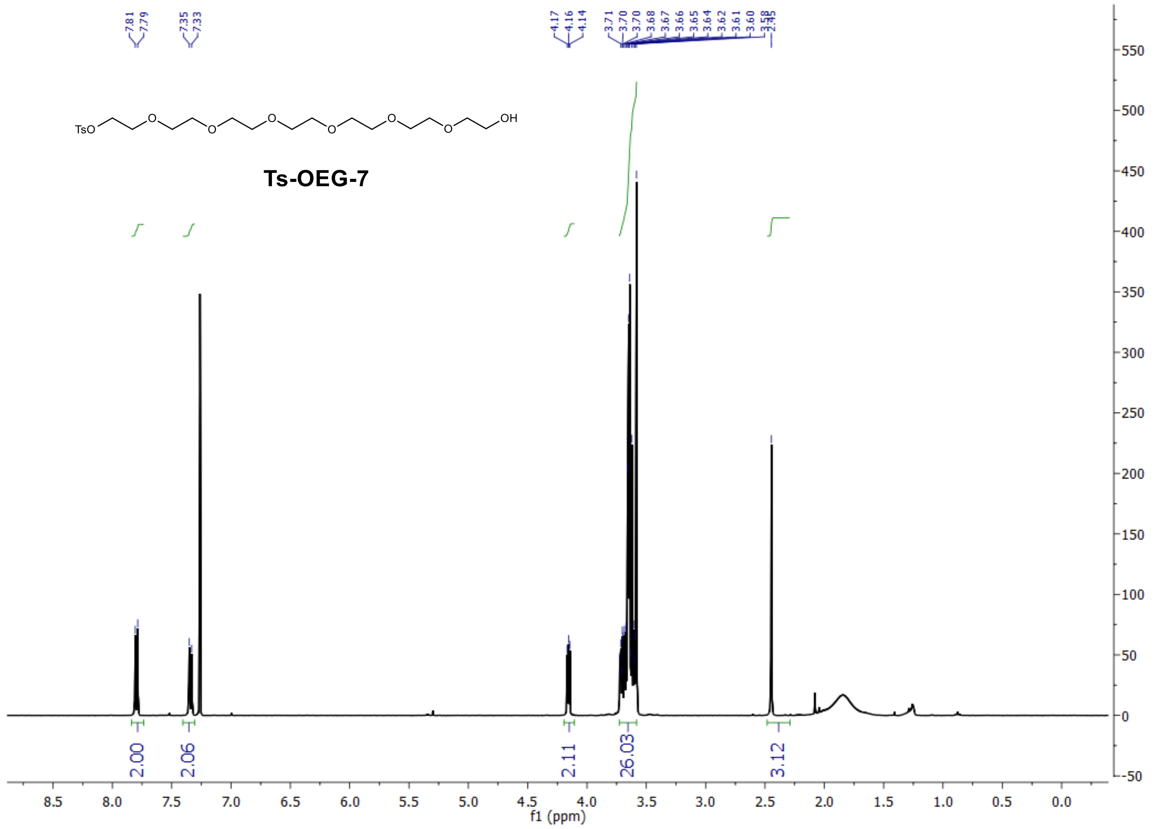

TsO
OH
Ts-OEG-7
7.81
7.79
7.35
7.33
4.17
4.16
4.14
3.71
3.70
3.70
3.68
3.67
3.66
3.65
3.64
3.62
3.61
3.60
3.58
2.45
2.00
2.06
2.11
26.03
3.12
f1 (ppm)

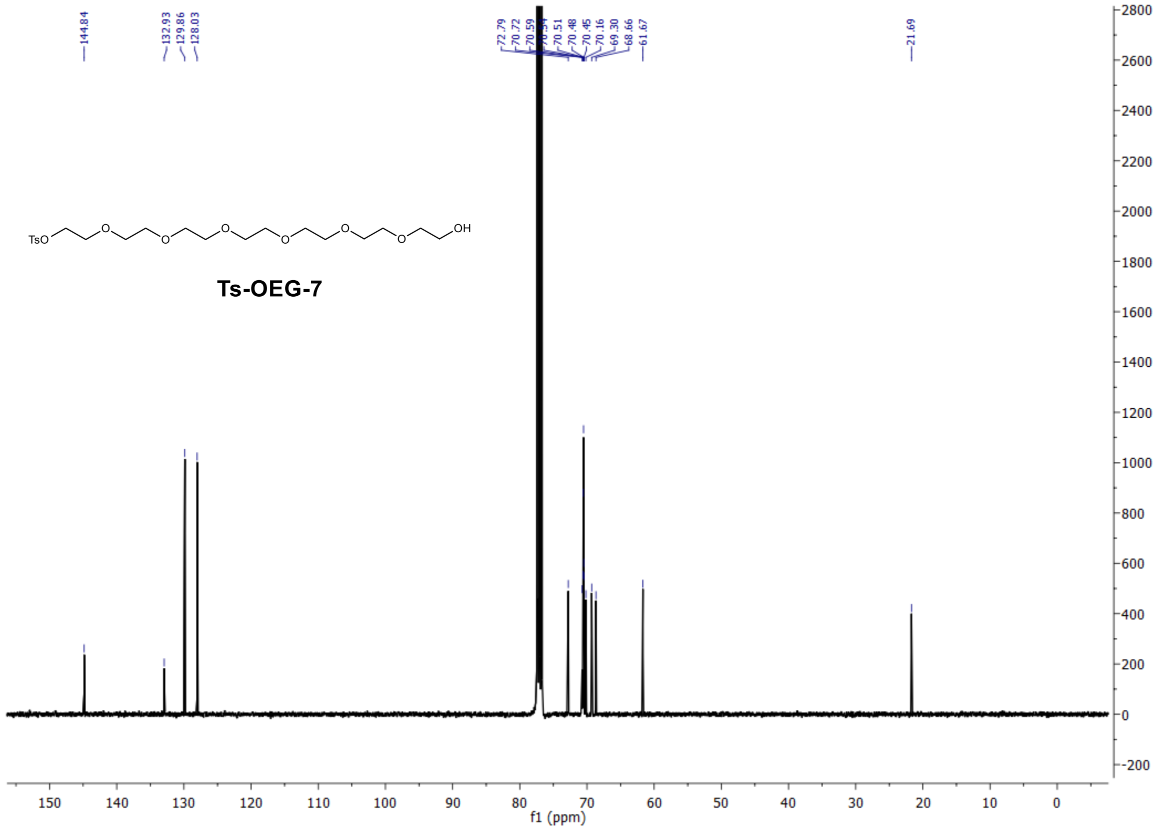

TsO
OH
Ts-OEG-7
144.84
132.93
129.86
128.03
72.79
70.72
70.59
70.51
70.48
70.45
70.16
69.30
68.66
61.67
21.69
f1 (ppm)

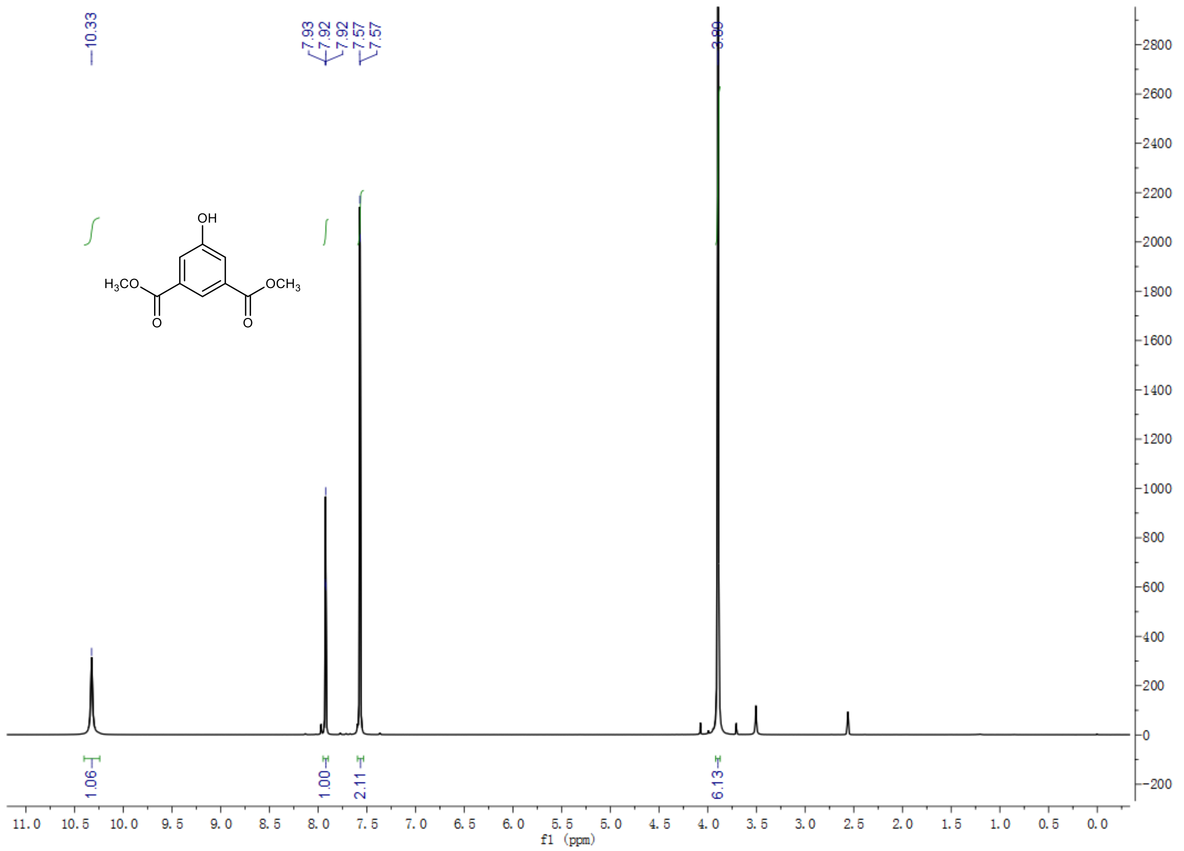

10.33
7.93
7.92
7.92
7.57
7.57
3.89
OH
H3CO
OCH3
O
O
1.06
1.00
2.11
6.13
f1 (ppm)

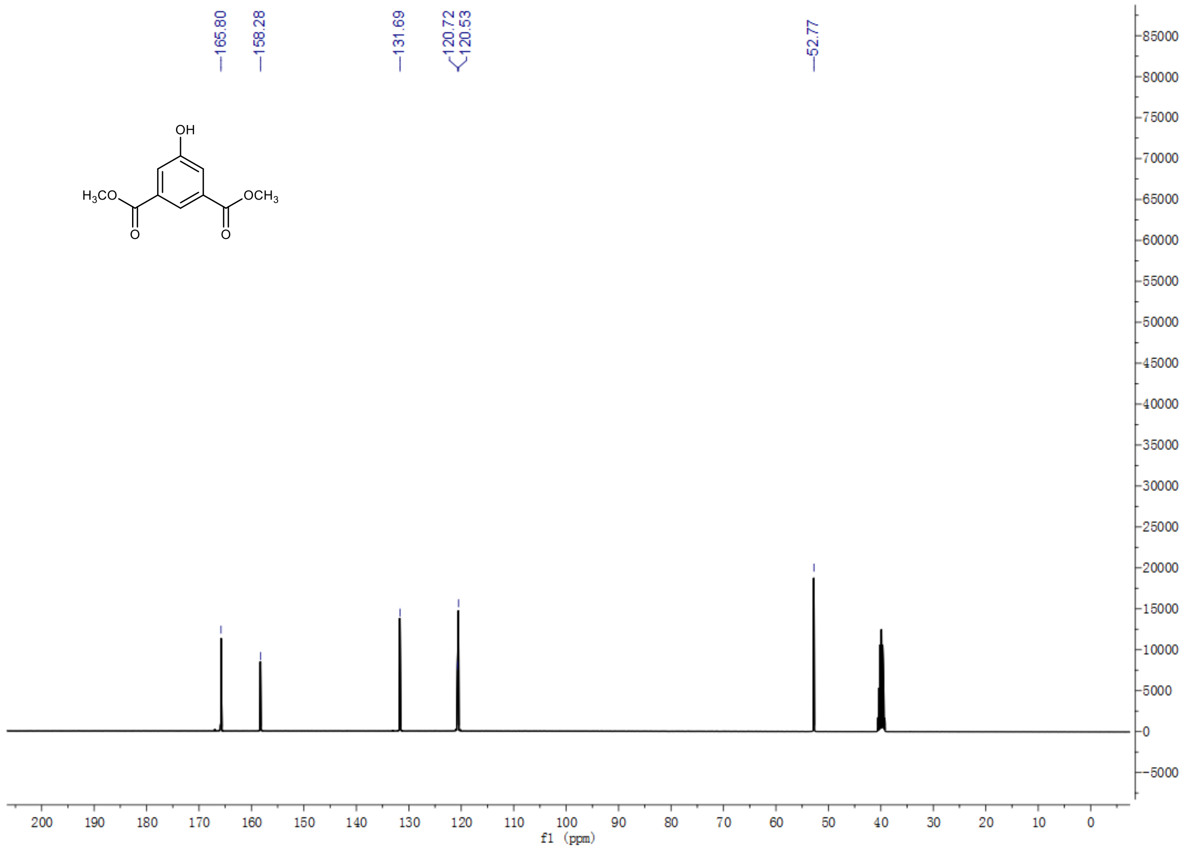

165.80
158.28
131.69
120.72
120.53
52.77
OH
H3CO
OCH3
O
O
f1 (ppm)

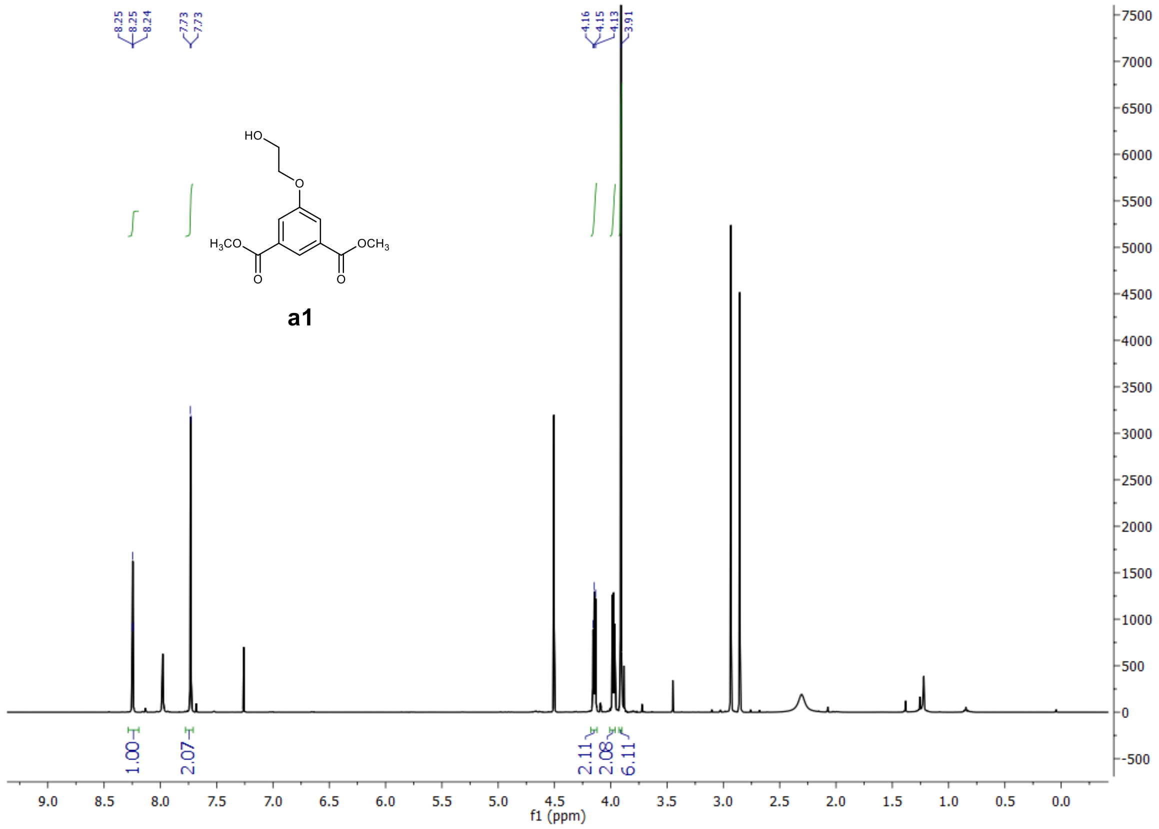

8.25
8.25
8.24
7.73
7.73
4.16
4.15
4.13
3.91
HO
O
H_3CO
OCH_3
O
O
1.00
2.07
2.11
2.08
6.11
f1 (ppm)

a1

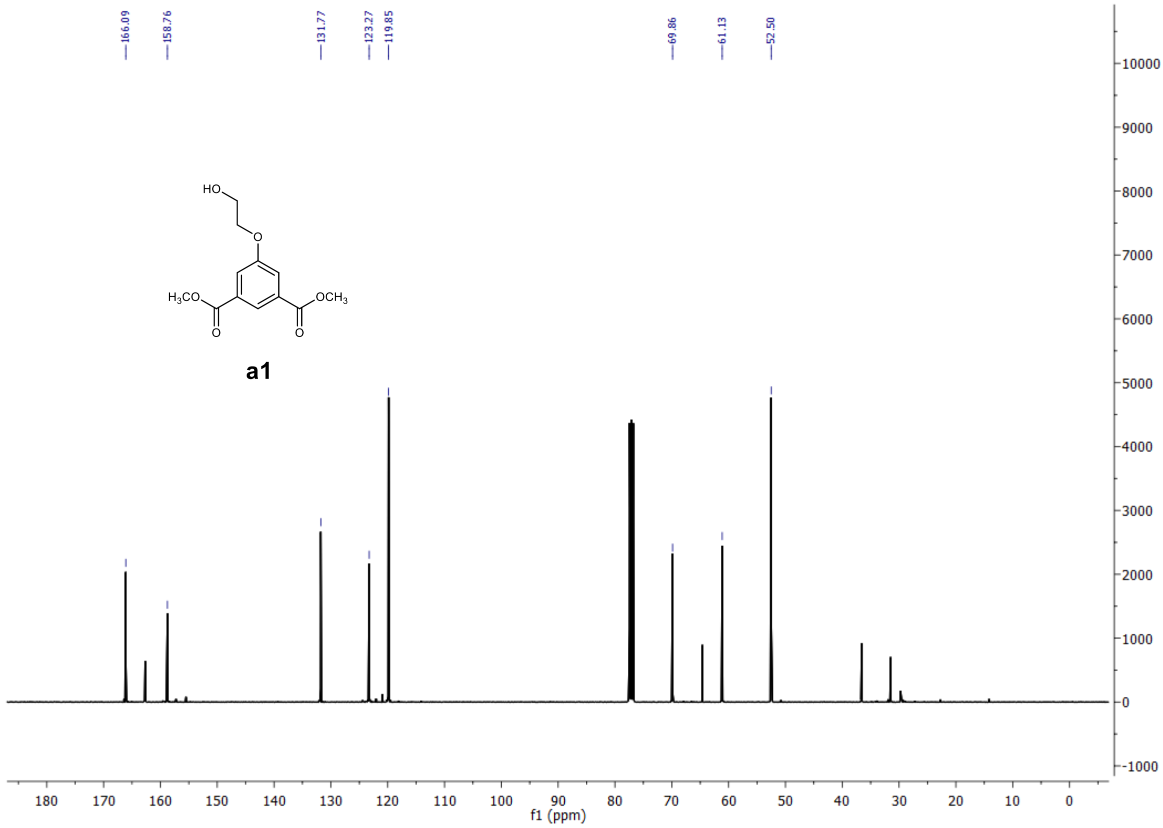

166.09
158.76
131.77
123.27
119.85
69.86
61.13
52.50
HO
O
H_3CO
OCH_3
O
O
f1 (ppm)

a1

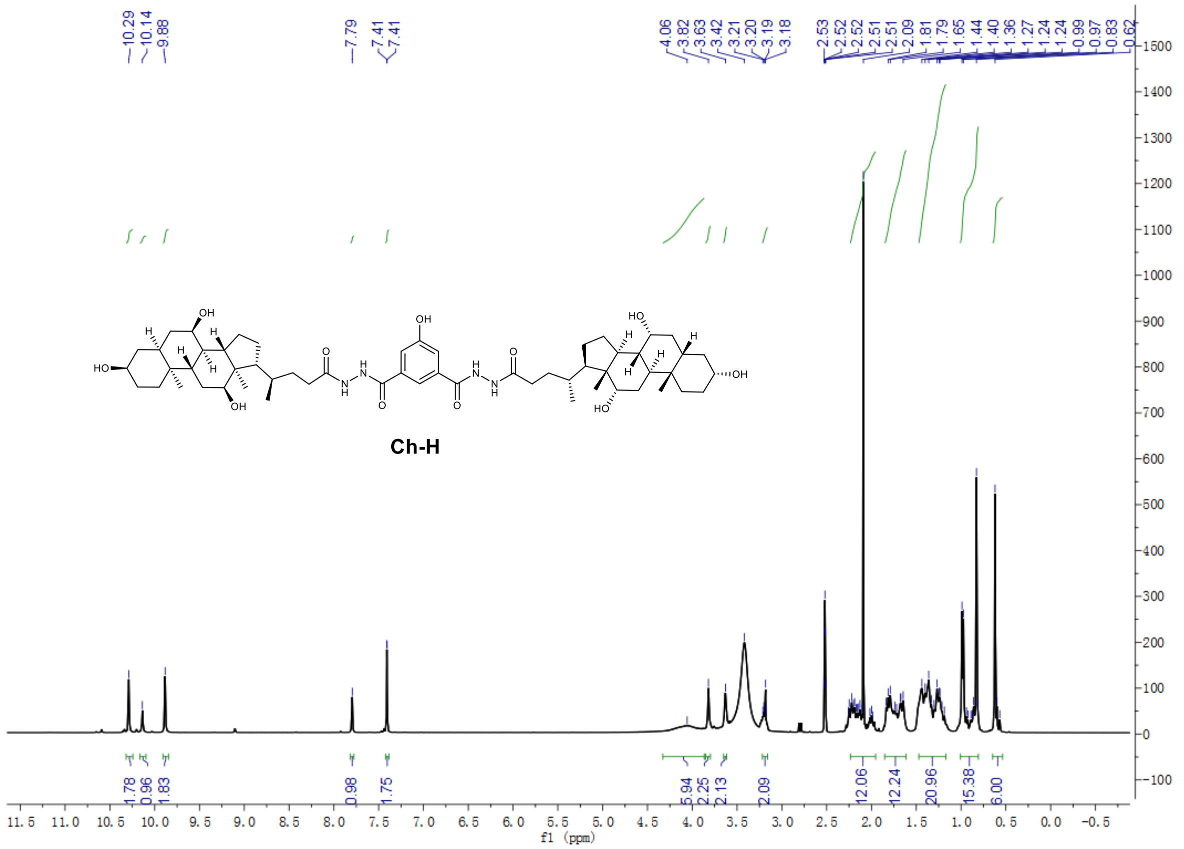
Ch-H
f1 (ppm)

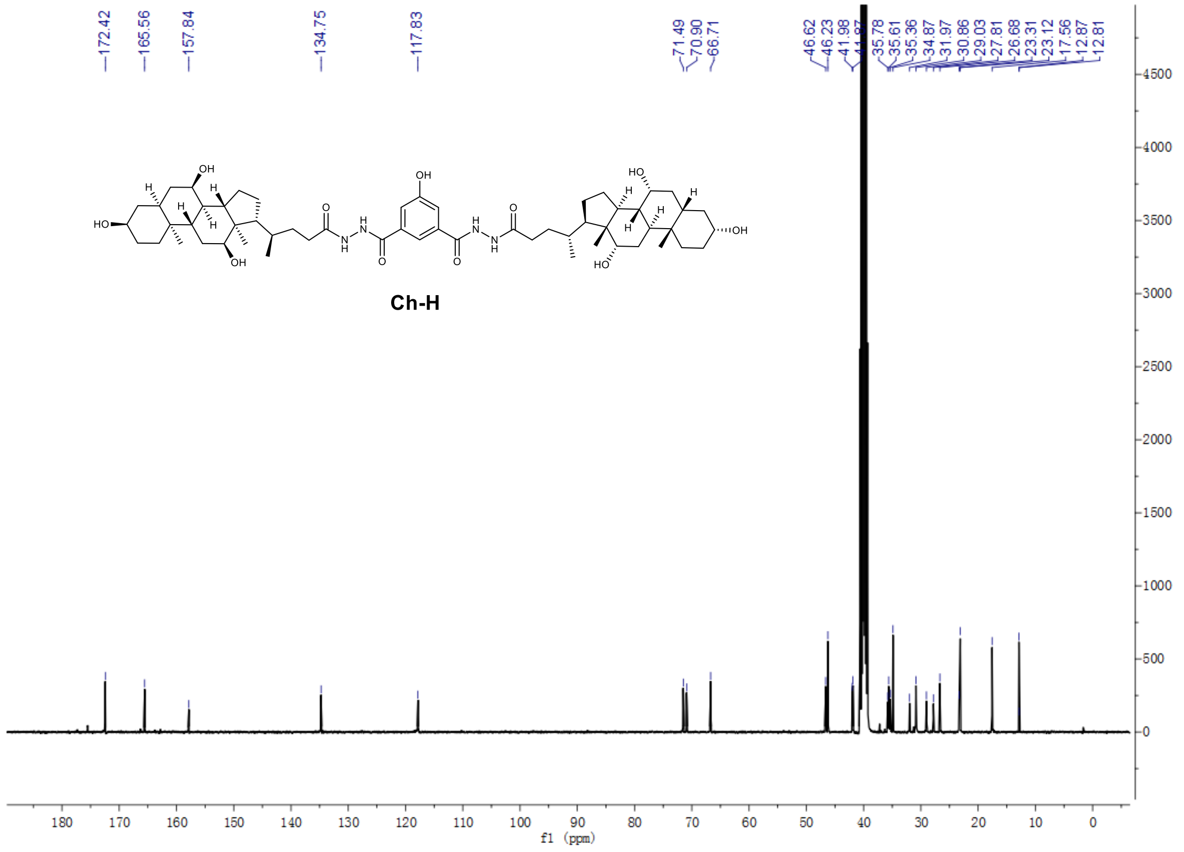
Ch-H
f1 (ppm)

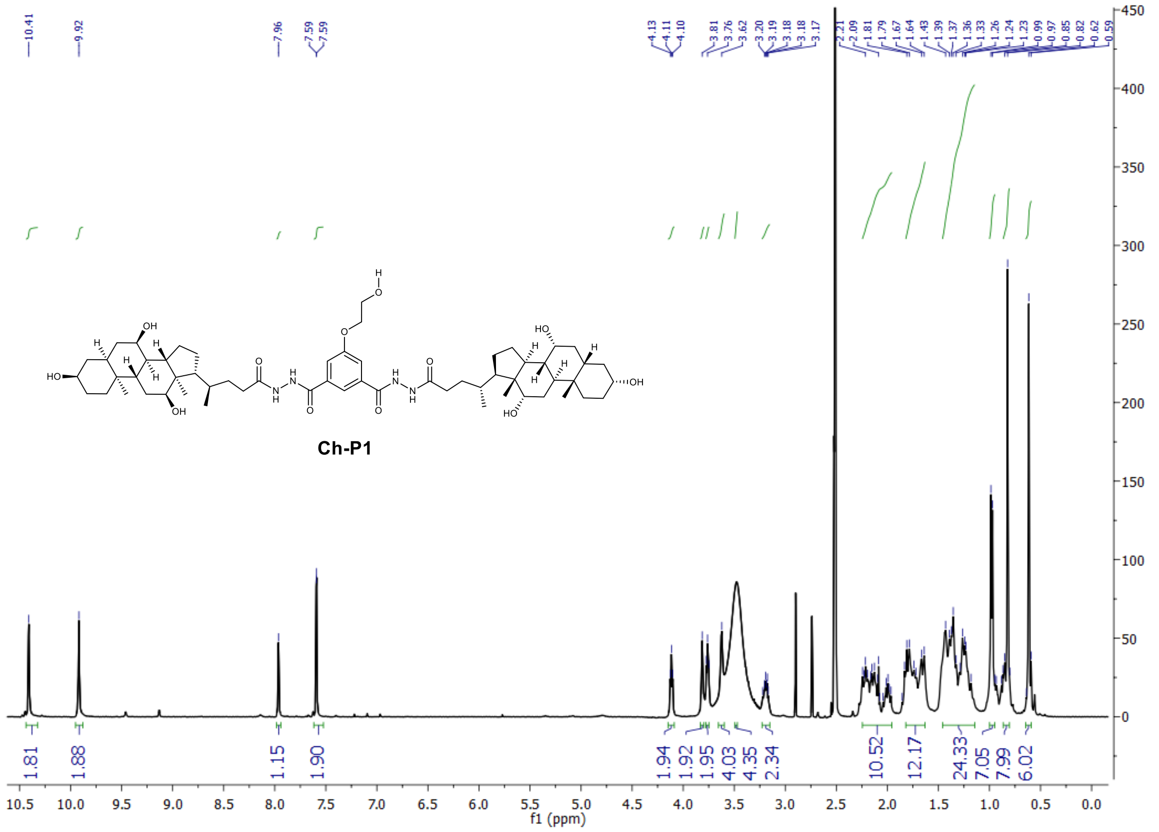
Ch-P1
f1 (ppm)

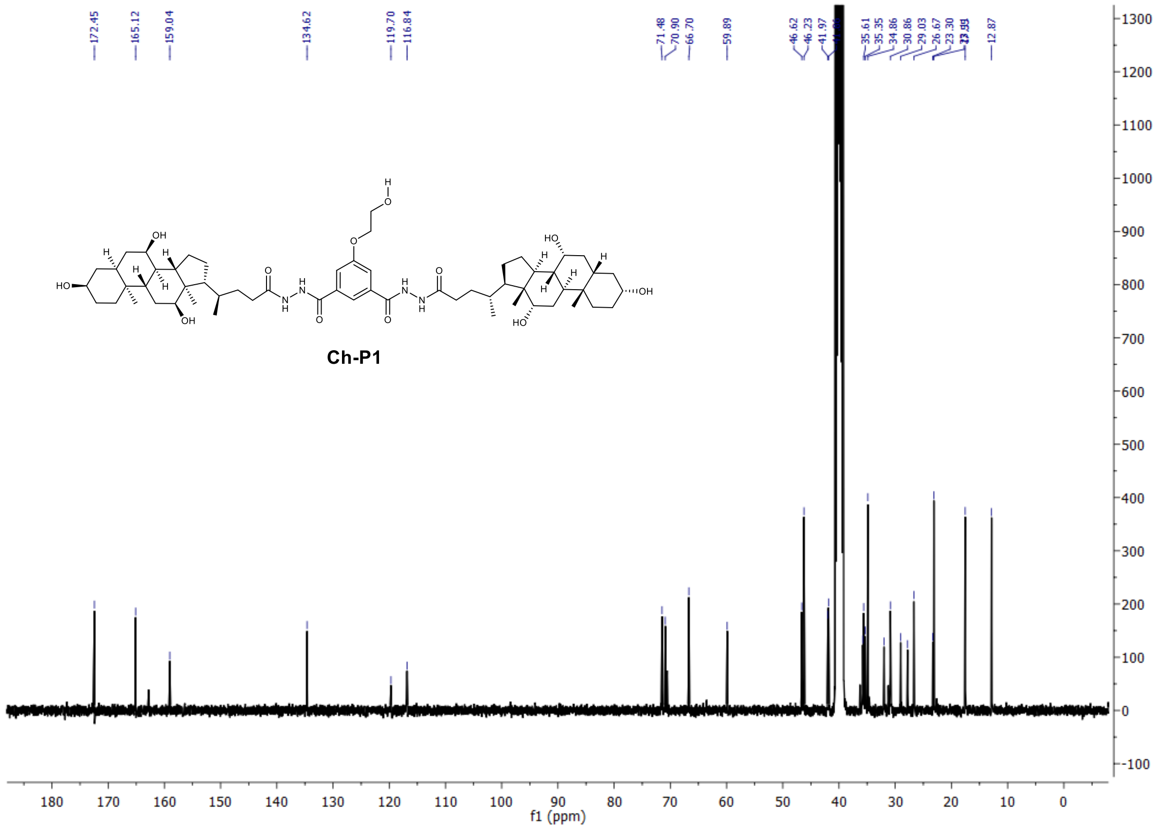
Ch-P1
f1 (ppm)

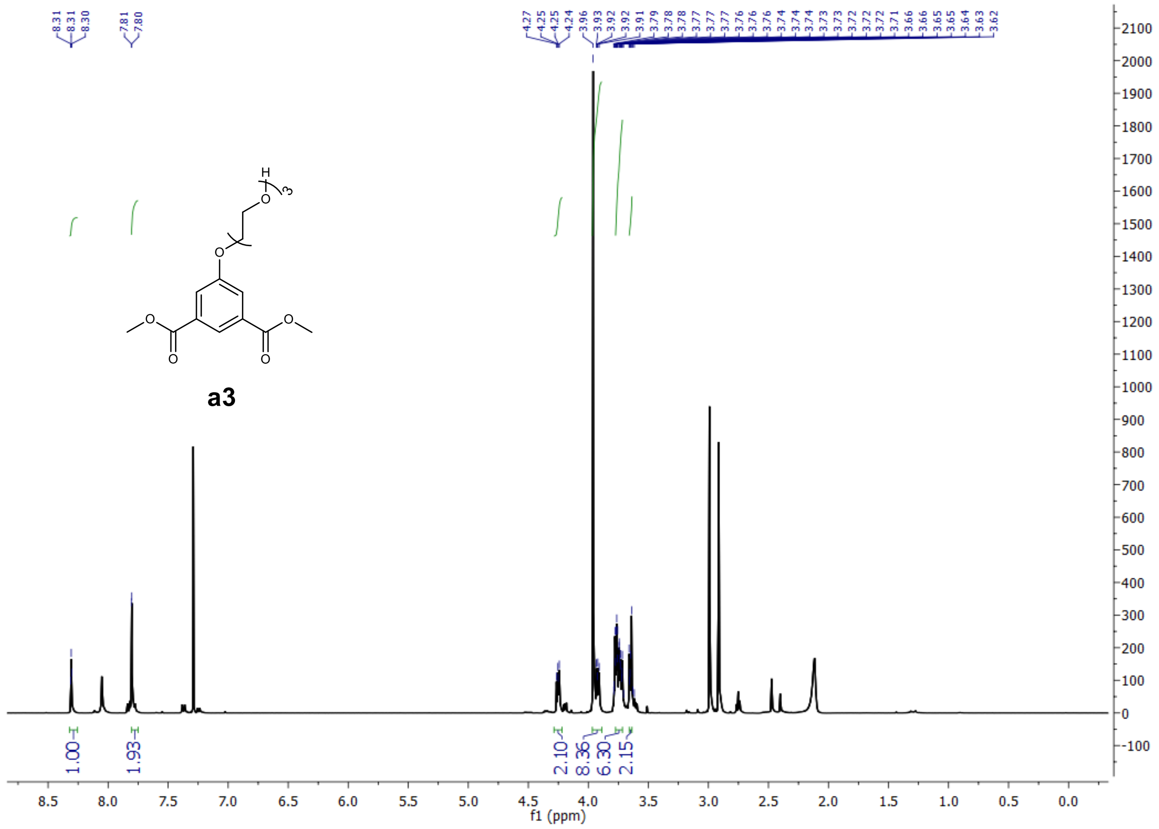
8.31
8.31
8.30
7.81
7.80
4.27
4.25
4.25
4.24
3.96
3.93
3.92
3.92
3.91
3.79
3.78
3.78
3.77
3.77
3.77
3.76
3.76
3.76
3.74
3.74
3.74
3.73
3.73
3.72
3.72
3.72
3.71
3.66
3.66
3.65
3.65
3.64
3.63
3.62
a3
1.00
1.93
2.10
8.36
6.30
2.15
f1 (ppm)

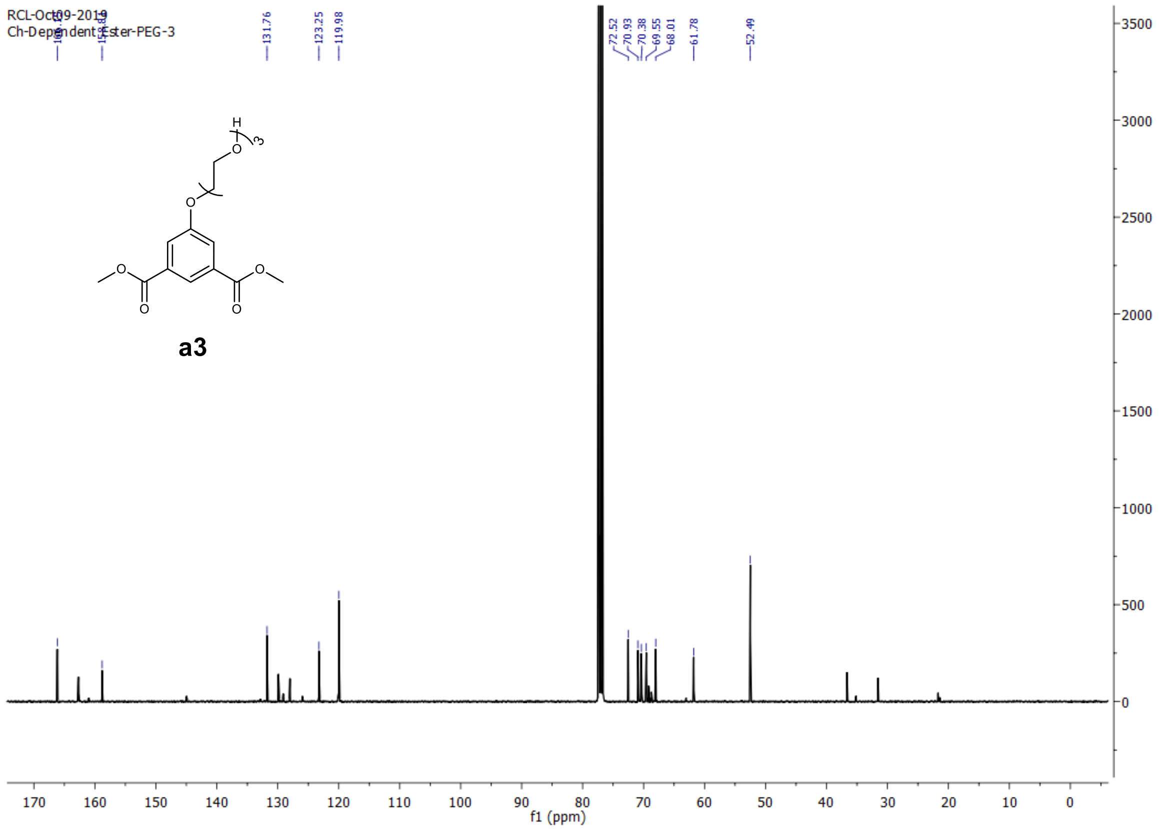
RCL-Oct09-2019
Ch-Dependent Ester-PEG-3
131.76
123.25
119.98
72.52
70.93
70.38
69.55
68.01
61.78
52.49
a3
f1 (ppm)

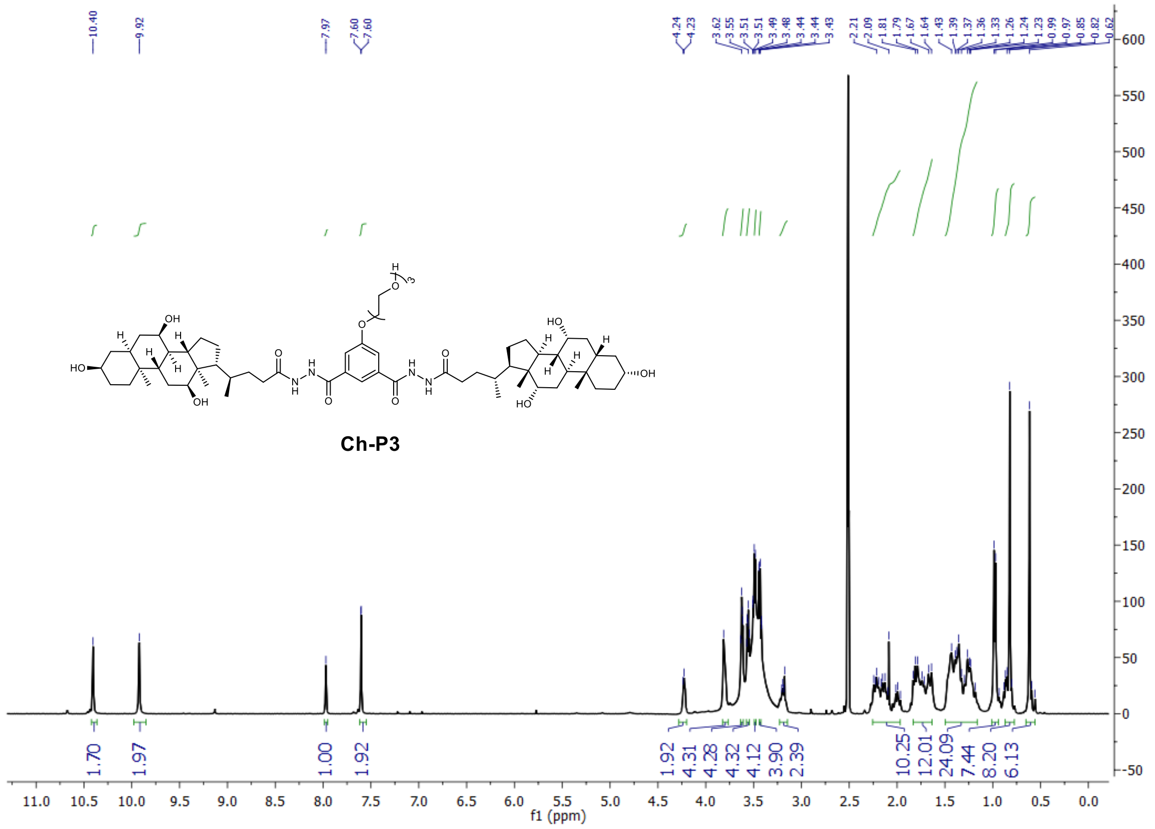

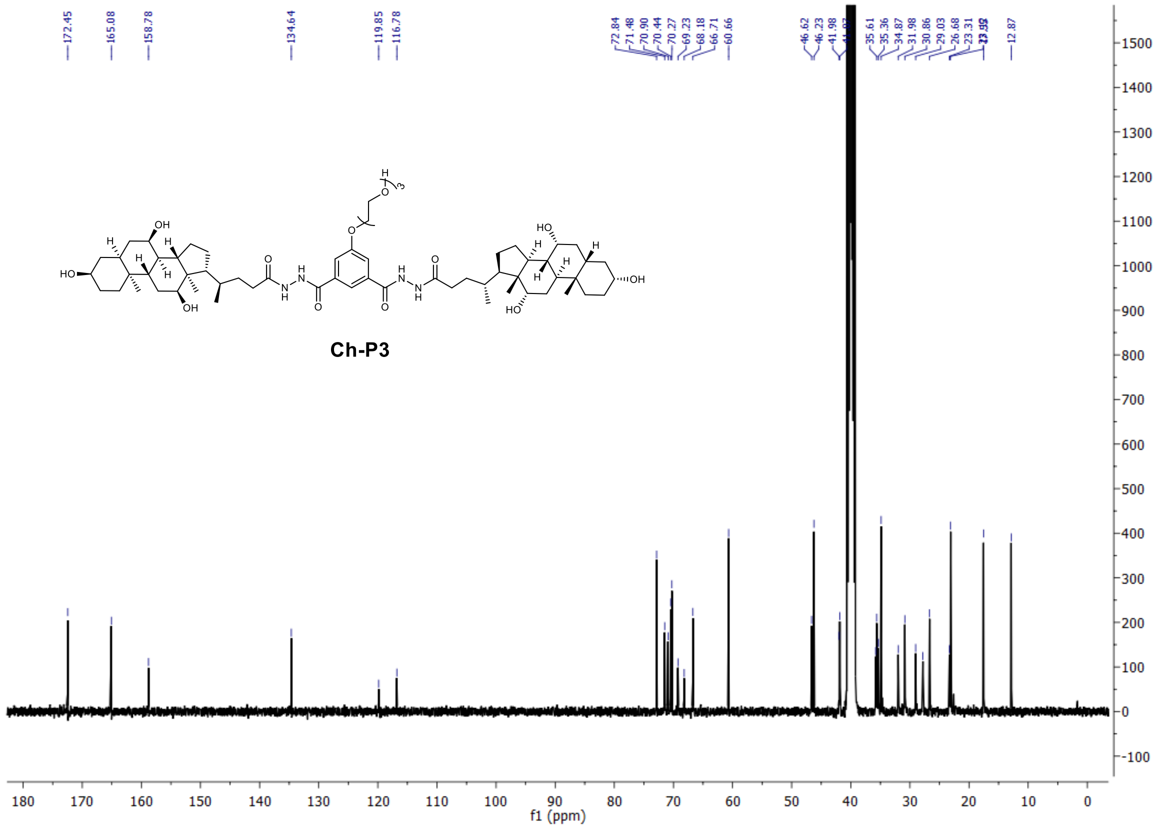

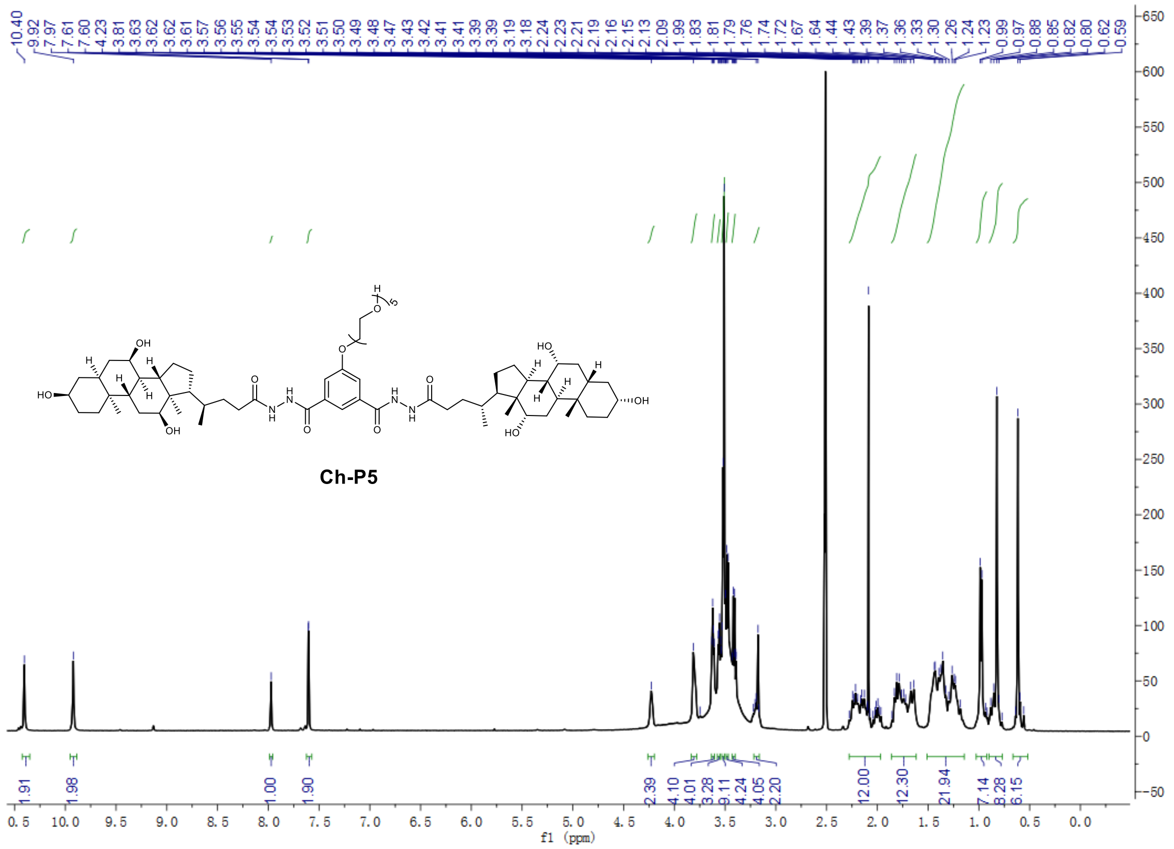
Ch-P5
f1 (ppm)

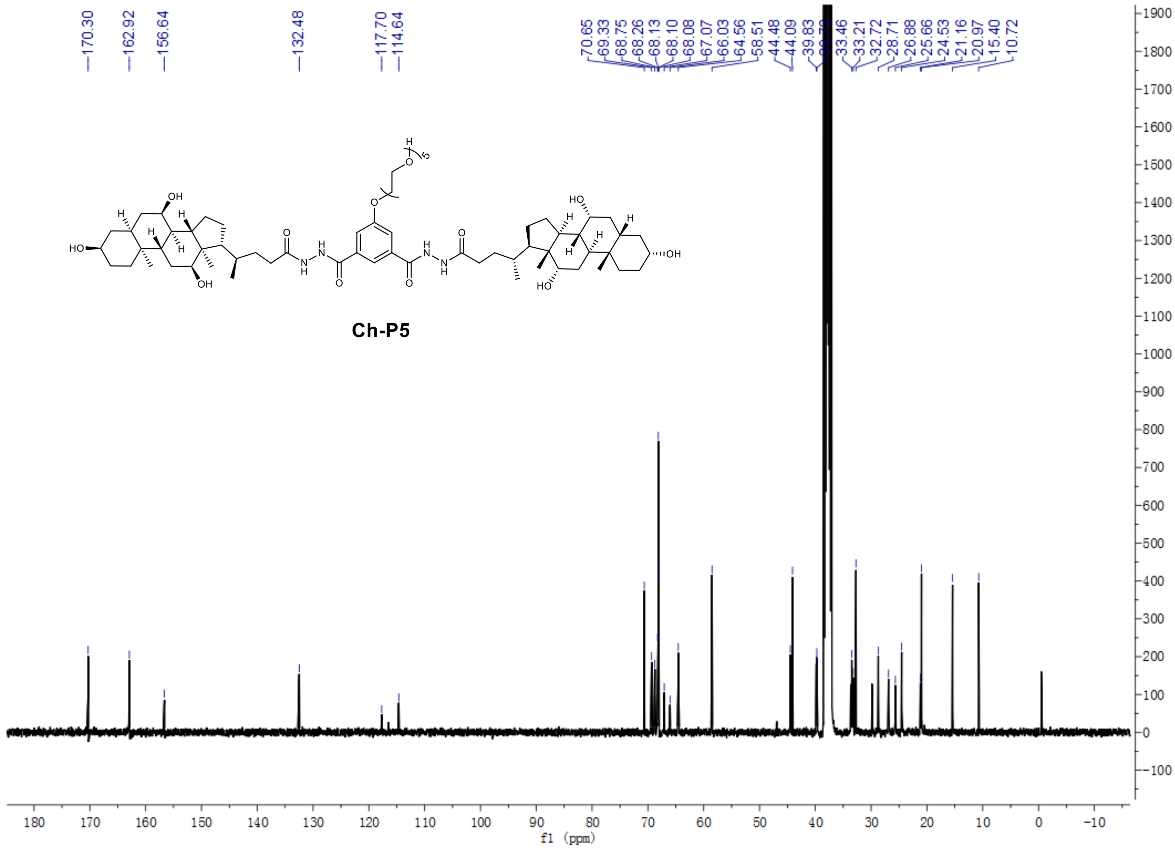
Ch-P5
f1 (ppm)

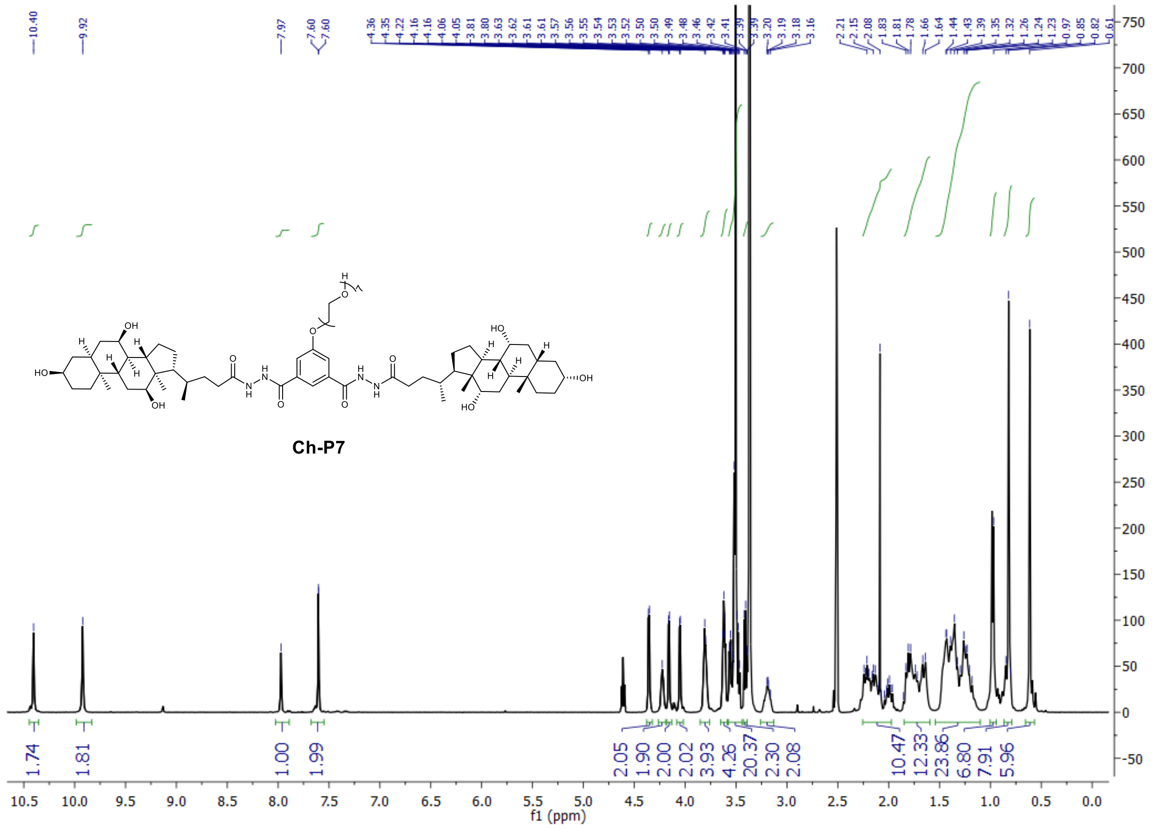

Ch-P7
f1 (ppm)

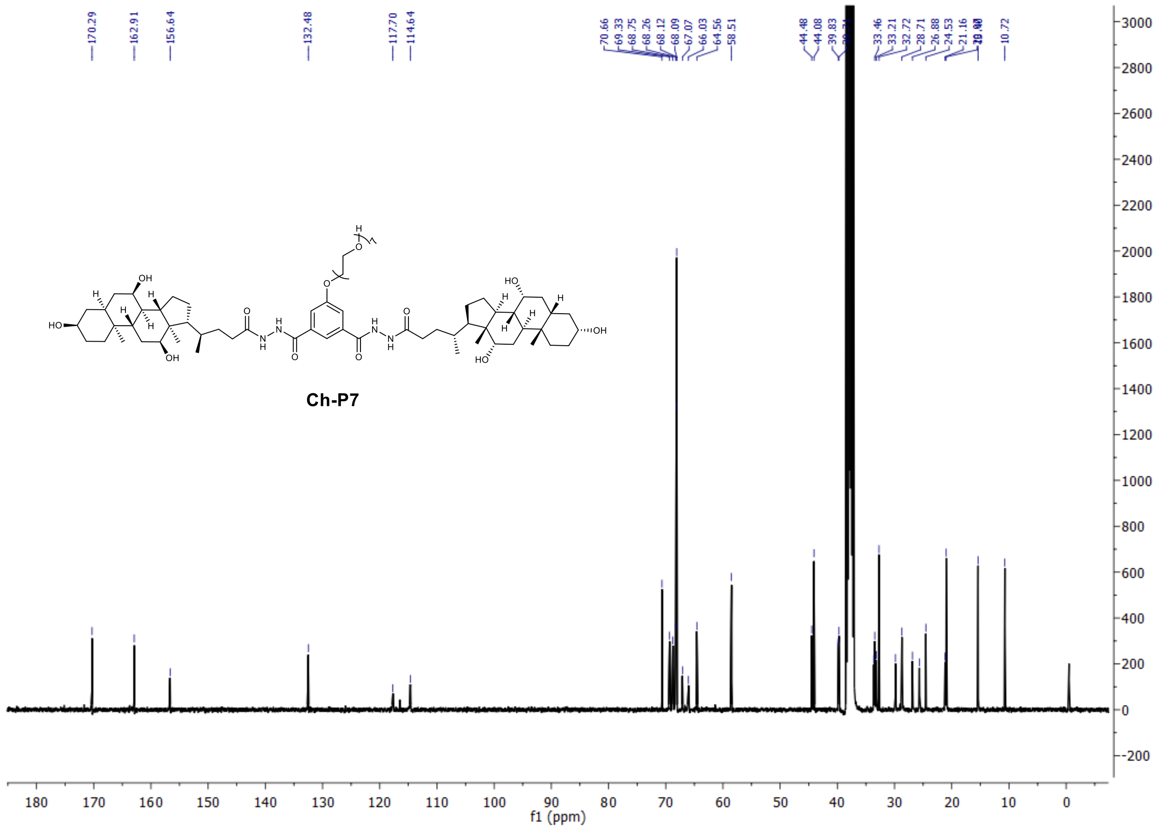

Ch-P7
f1 (ppm)

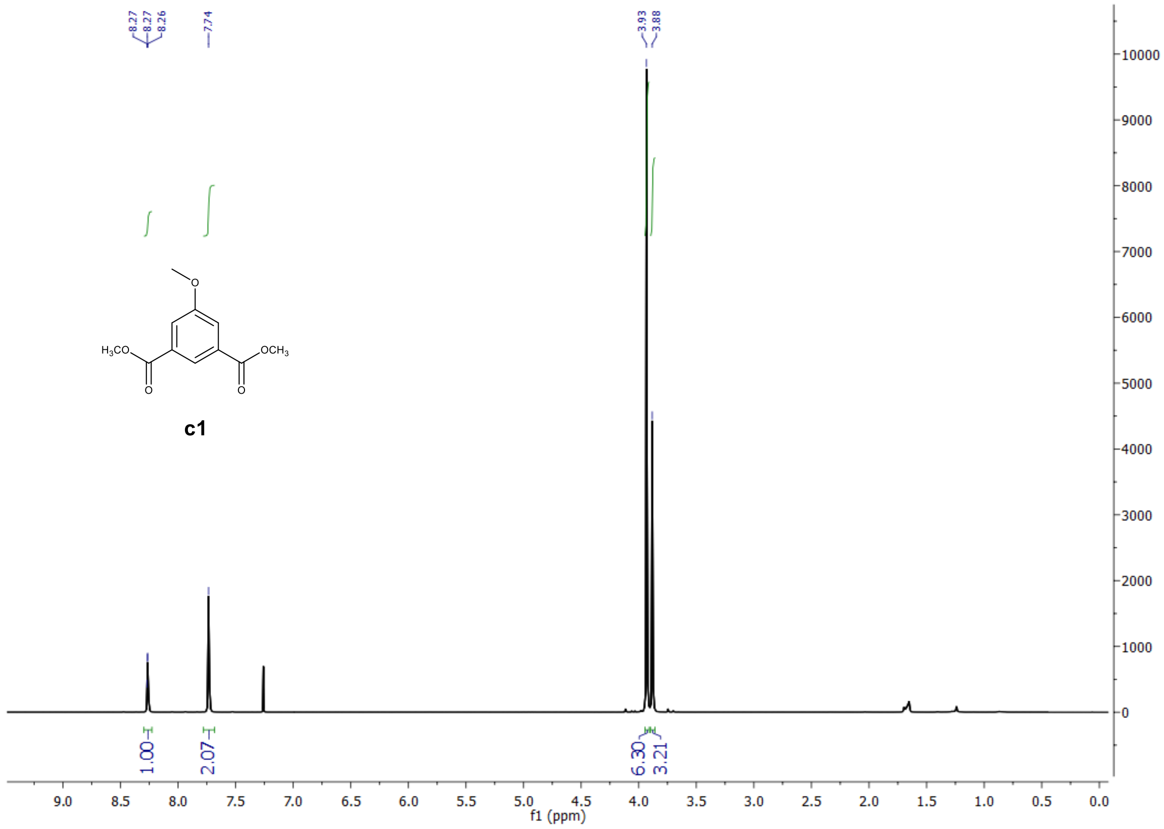
8.27
8.27
8.26
7.74
3.93
3.88
H3CO
OCH3
O
O
O
c1
1.00
2.07
6.30
3.21
f1 (ppm)

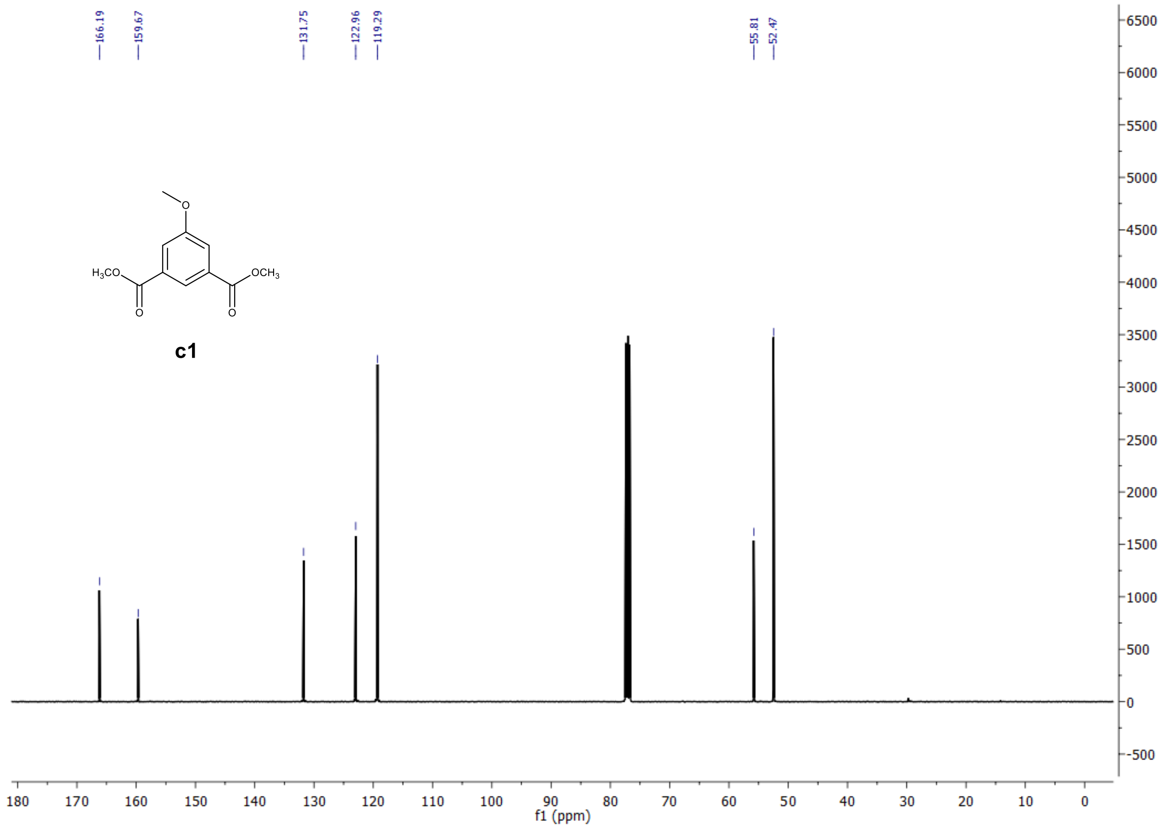
166.19
159.67
131.75
122.96
119.29
55.81
52.47
H3CO
OCH3
O
O
O
c1
f1 (ppm)

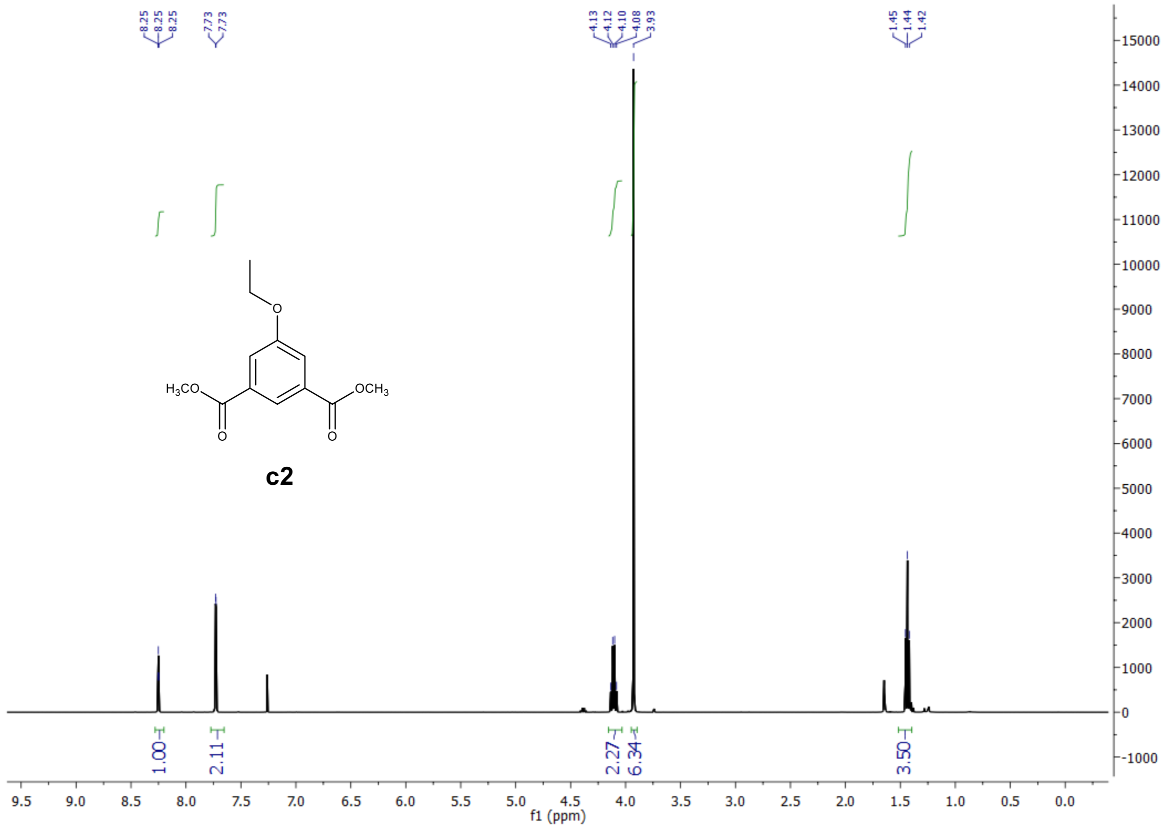
8.25
8.25
8.25
7.73
7.73
4.13
4.12
4.10
4.08
3.93
1.45
1.44
1.42
H3CO
OCH3
O
1.00
2.11
2.27
6.34
3.50
f1 (ppm)

c2

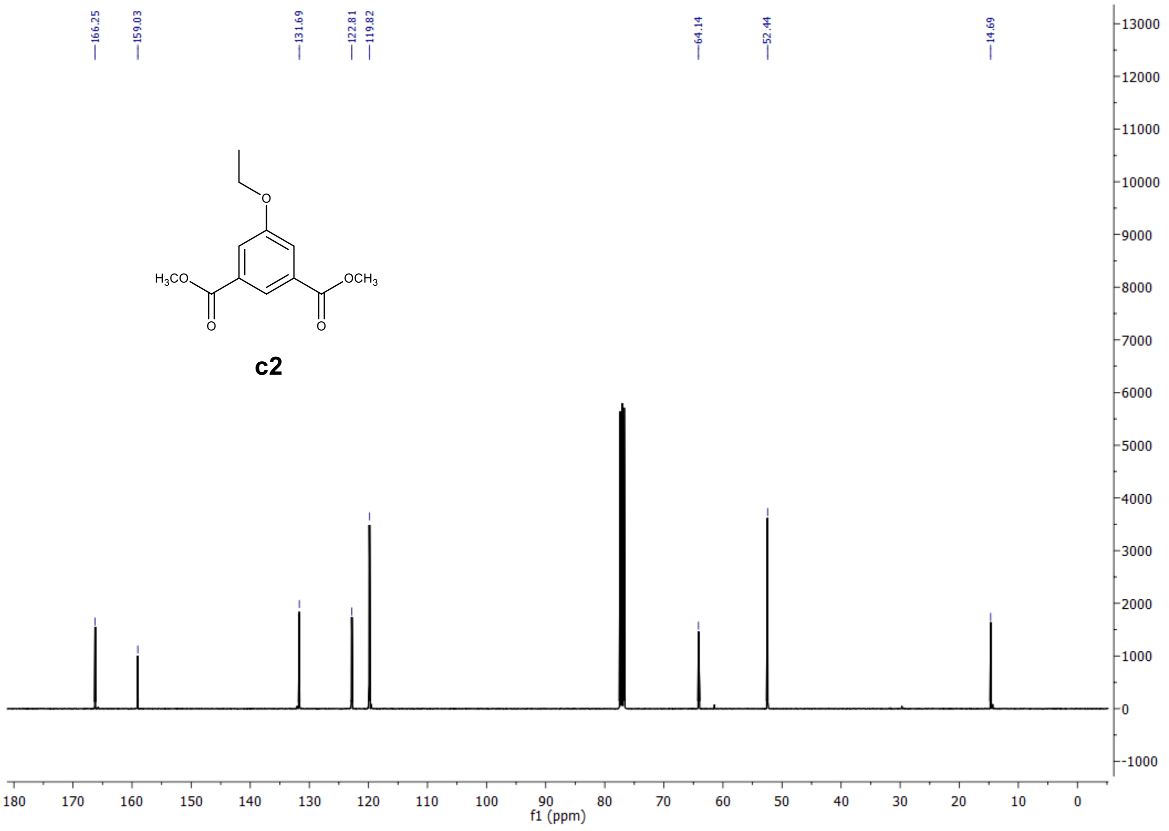
166.25
159.03
131.69
122.81
119.82
64.14
52.44
14.69
H3CO
OCH3
O
f1 (ppm)

c2

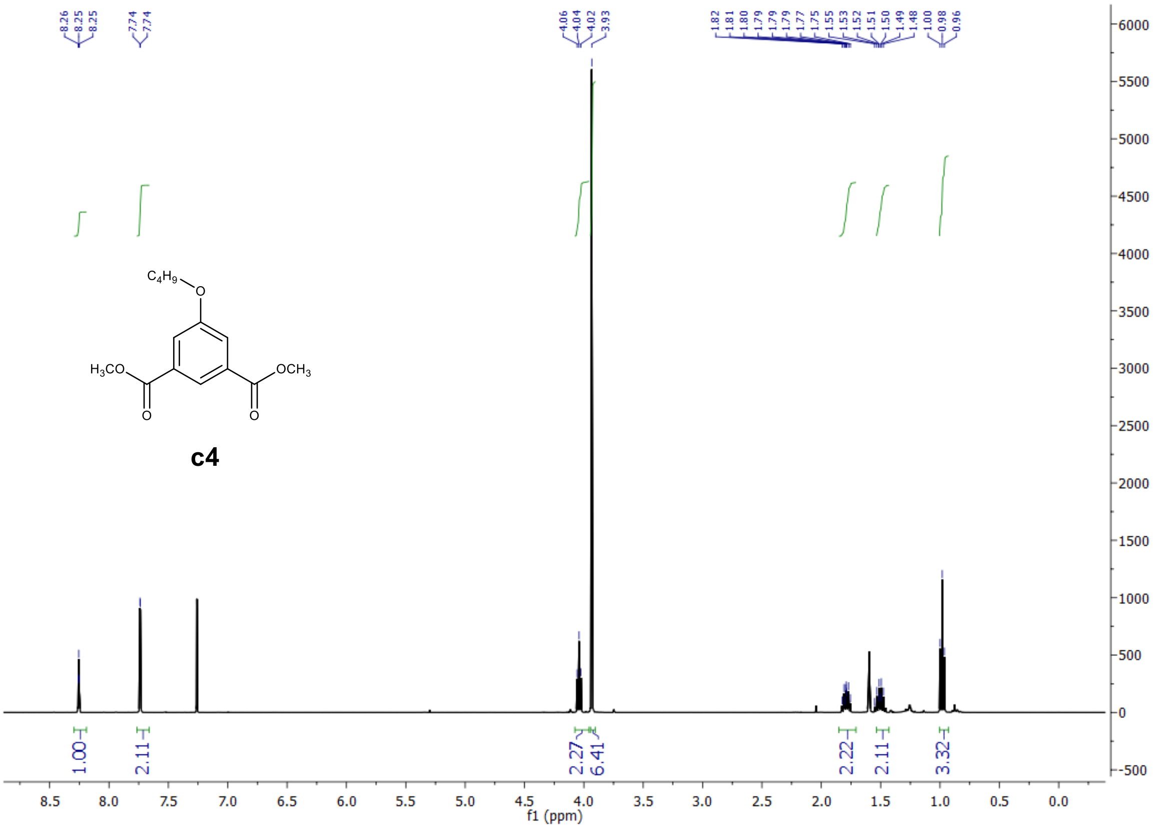
8.26
8.25
8.25
7.74
7.74
4.06
4.04
4.02
3.93
1.82
1.81
1.80
1.79
1.79
1.79
1.77
1.75
1.55
1.53
1.52
1.51
1.50
1.49
1.48
1.00
0.98
0.96
C_4H_9
O
H_3CO
OCH_3
O
O
c4
1.00
2.11
2.27
6.41
2.22
2.11
3.32
f1 (ppm)

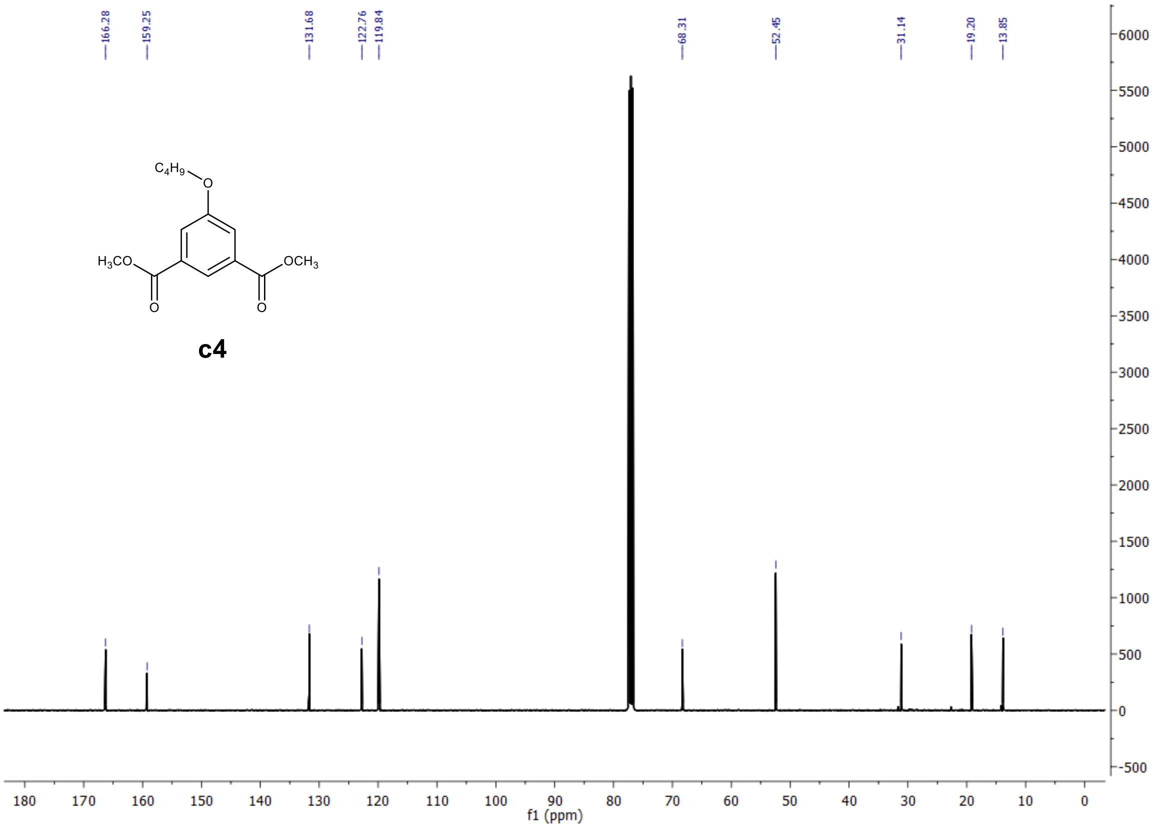
166.28
159.25
131.68
122.76
119.84
68.31
52.45
31.14
19.20
13.85
C_4H_9
O
H_3CO
OCH_3
O
O
c4
f1 (ppm)

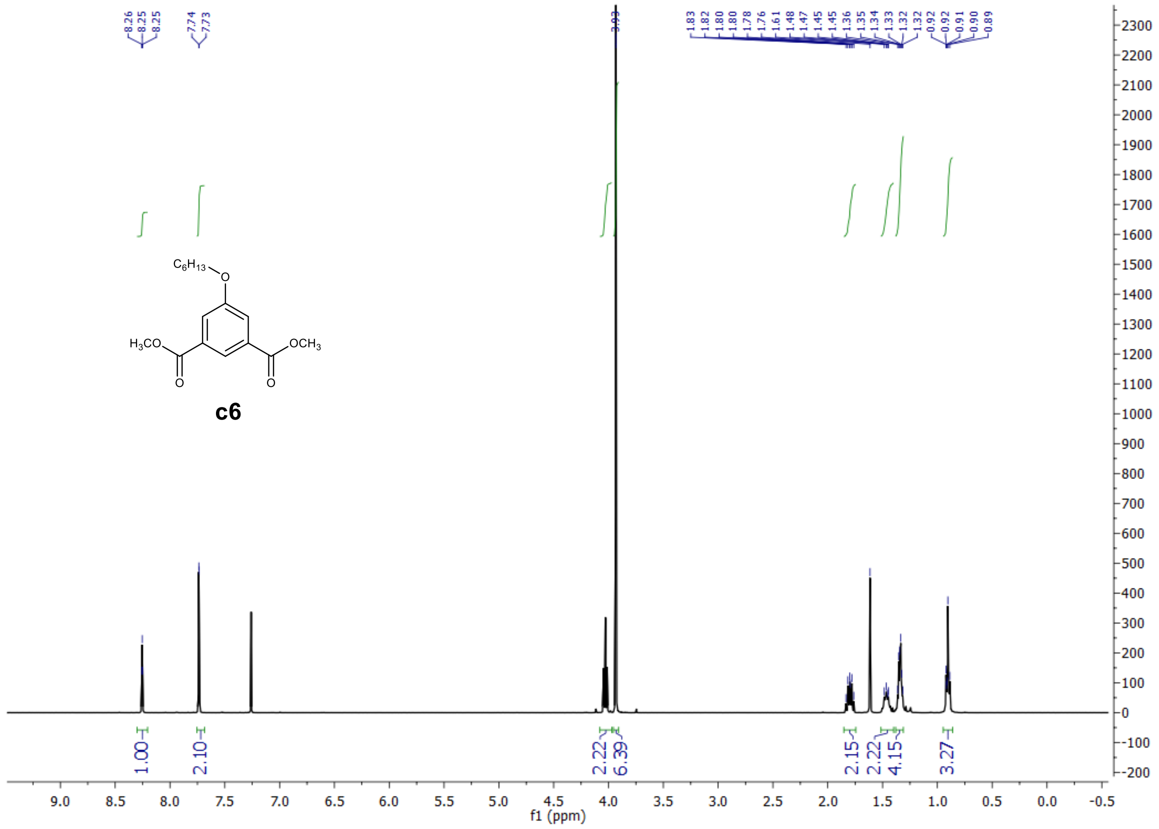
c6

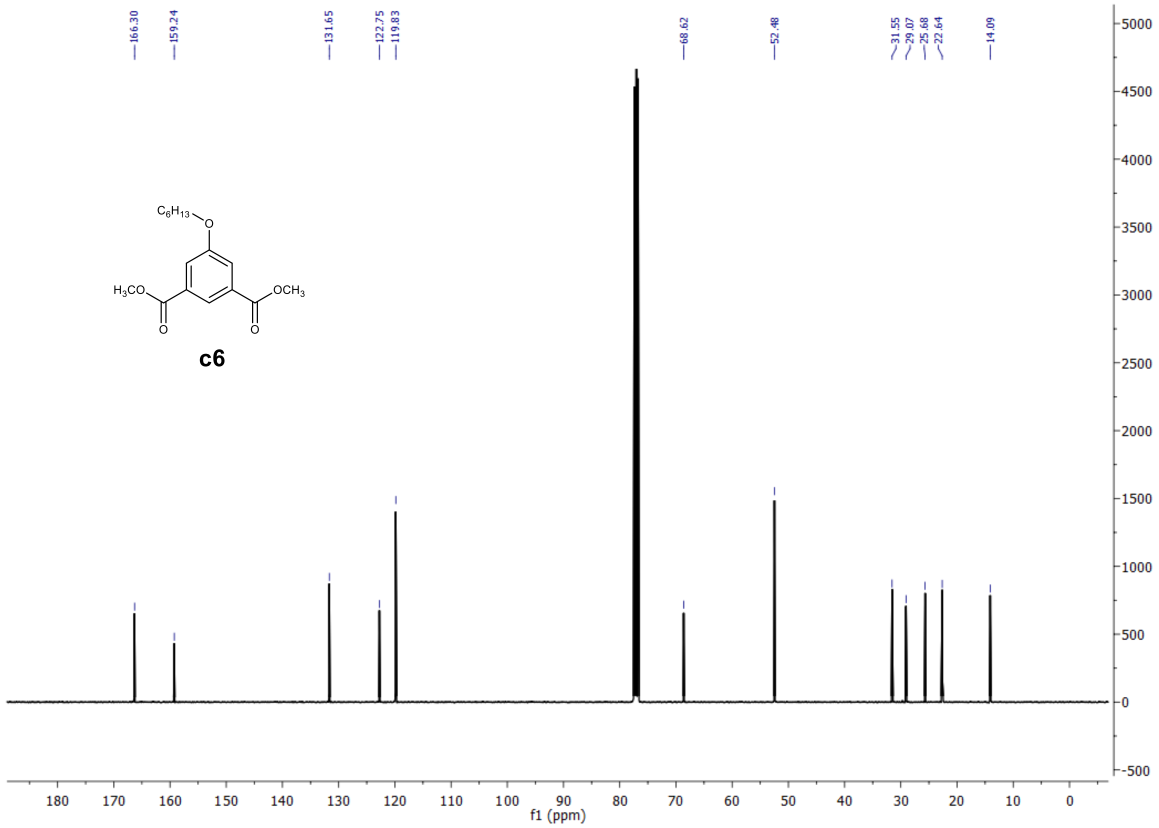
c6

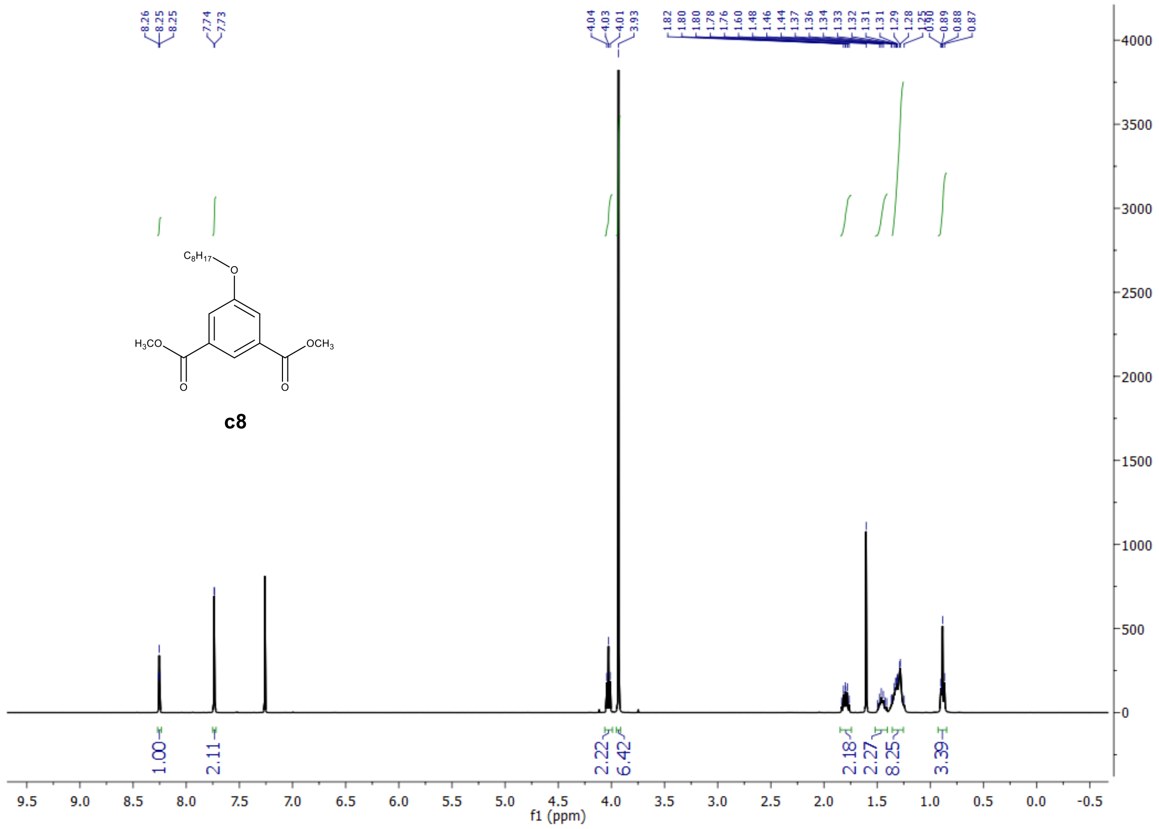

8.26
8.25
8.25
7.74
7.73
4.04
4.03
4.01
3.93
1.82
1.80
1.80
1.78
1.76
1.60
1.48
1.46
1.44
1.37
1.36
1.34
1.33
1.32
1.31
1.31
1.29
1.28
0.89
0.88
0.87
C_8H_{17}
O
H_3CO
OCH_3
c8
1.00
2.11
2.22
6.42
2.18
2.27
8.25
3.39
f1 (ppm)

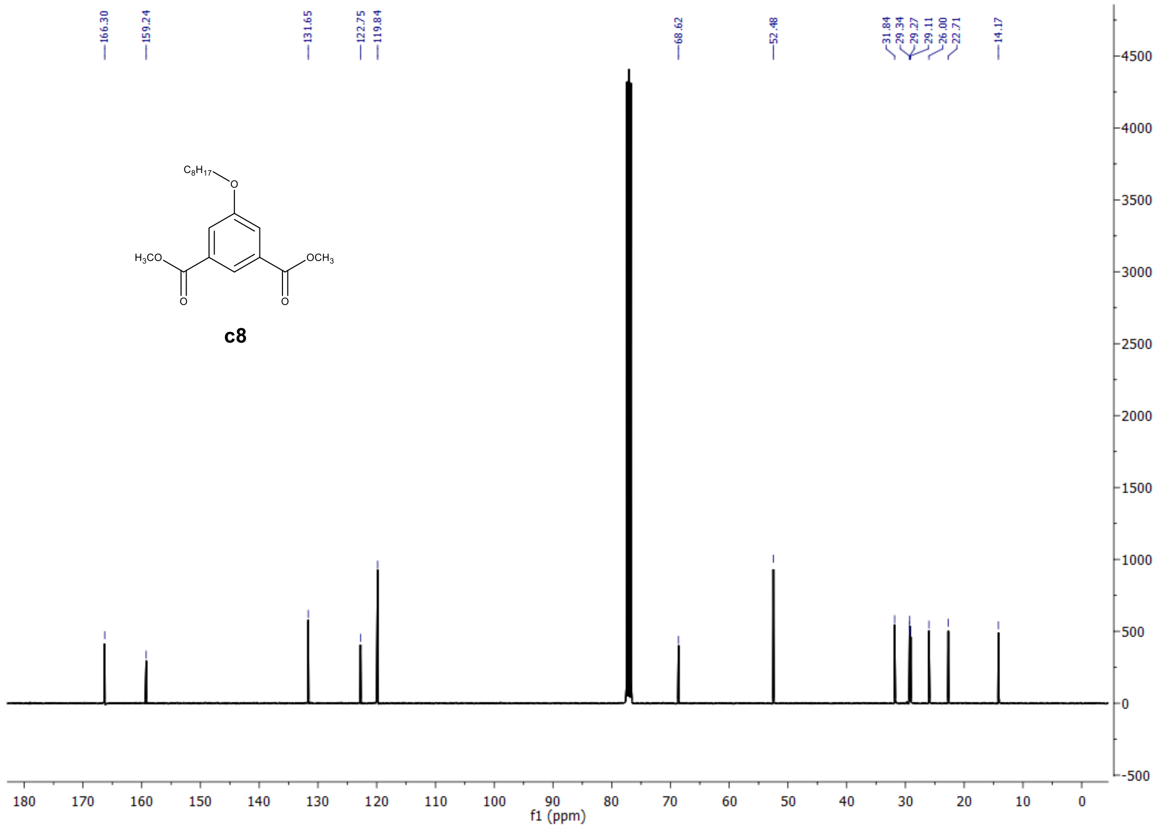

166.30
159.24
131.65
122.75
119.84
68.62
52.48
31.84
29.34
29.27
29.11
26.00
22.71
14.17
C_8H_{17}
O
H_3CO
OCH_3
c8
f1 (ppm)

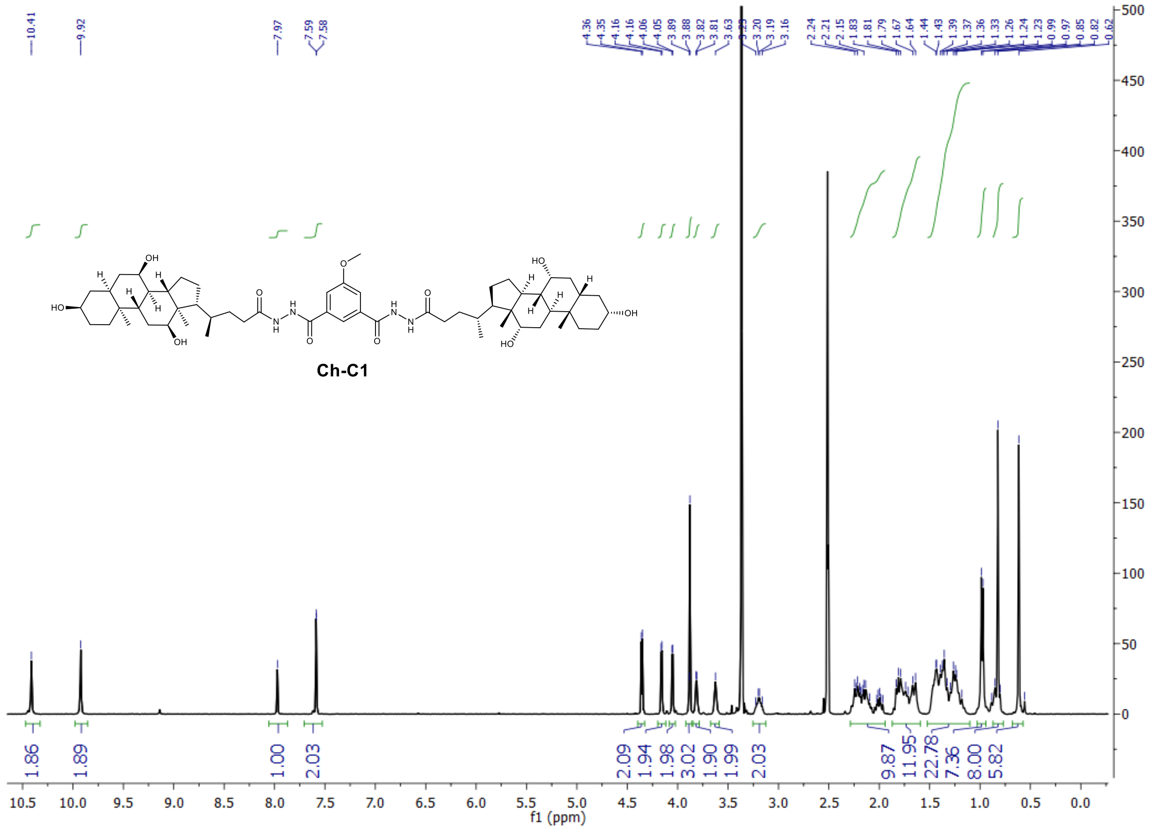
Ch-C1
f1 (ppm)

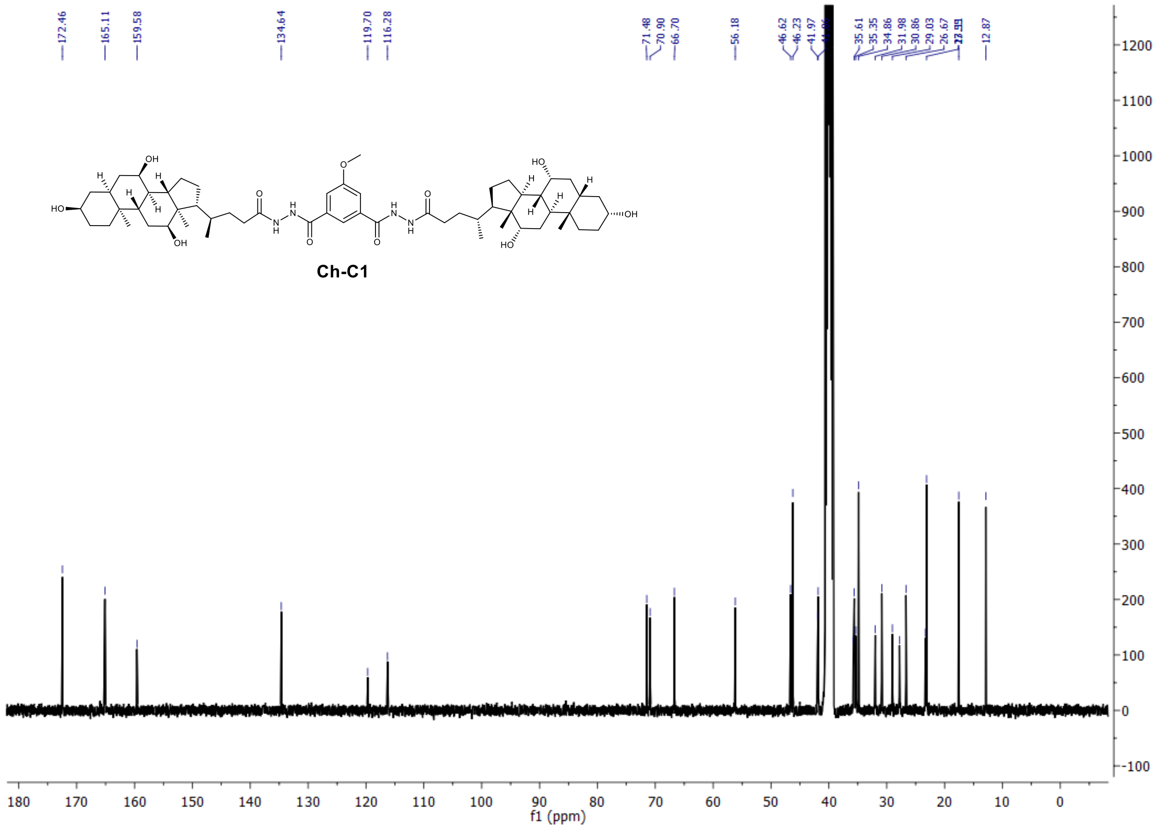
Ch-C1
f1 (ppm)

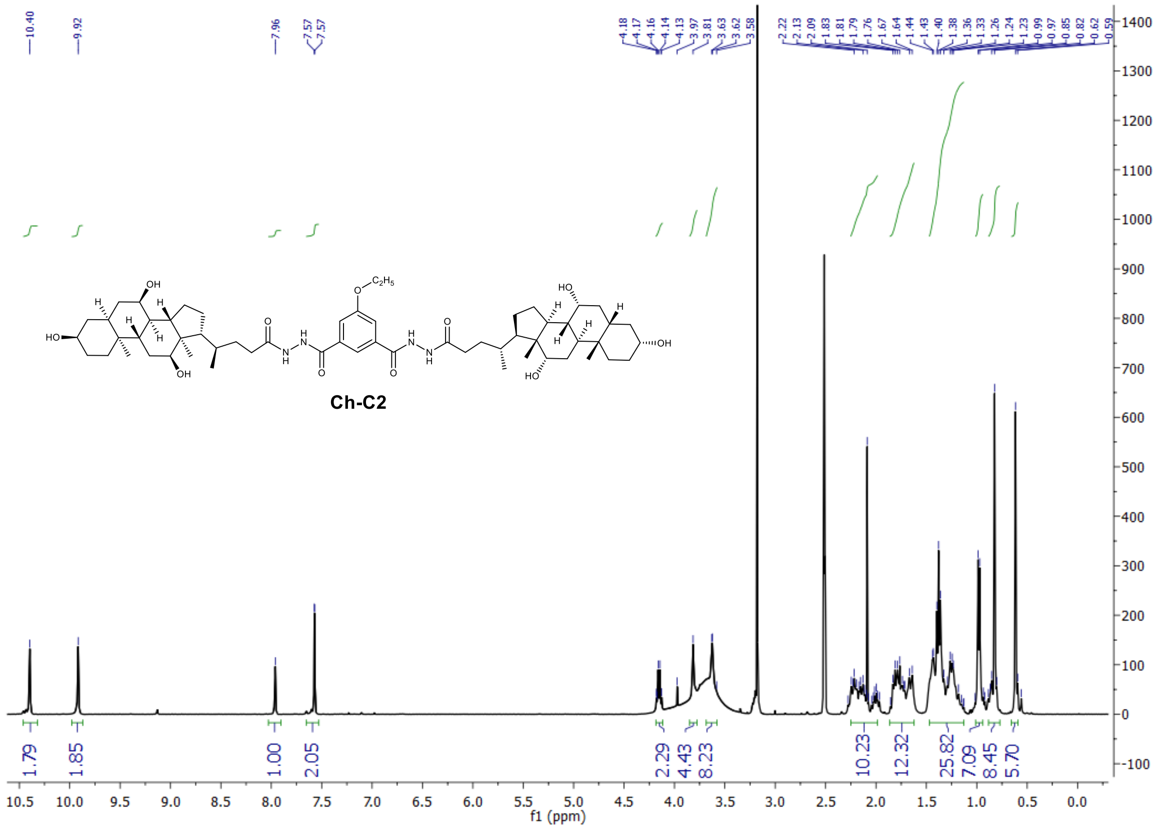
10.40
9.92
7.96
7.57
7.57
4.18
4.17
4.16
4.14
4.13
3.97
3.81
3.63
3.62
3.58
2.22
2.13
2.09
1.83
1.81
1.79
1.76
1.67
1.64
1.44
1.43
1.40
1.38
1.36
1.33
1.26
1.24
1.23
0.99
0.97
0.85
0.82
0.62
0.59
Ch-C2
1.79
1.85
1.00
2.05
2.29
4.43
8.23
10.23
12.32
25.82
7.09
8.45
5.70
f1 (ppm)

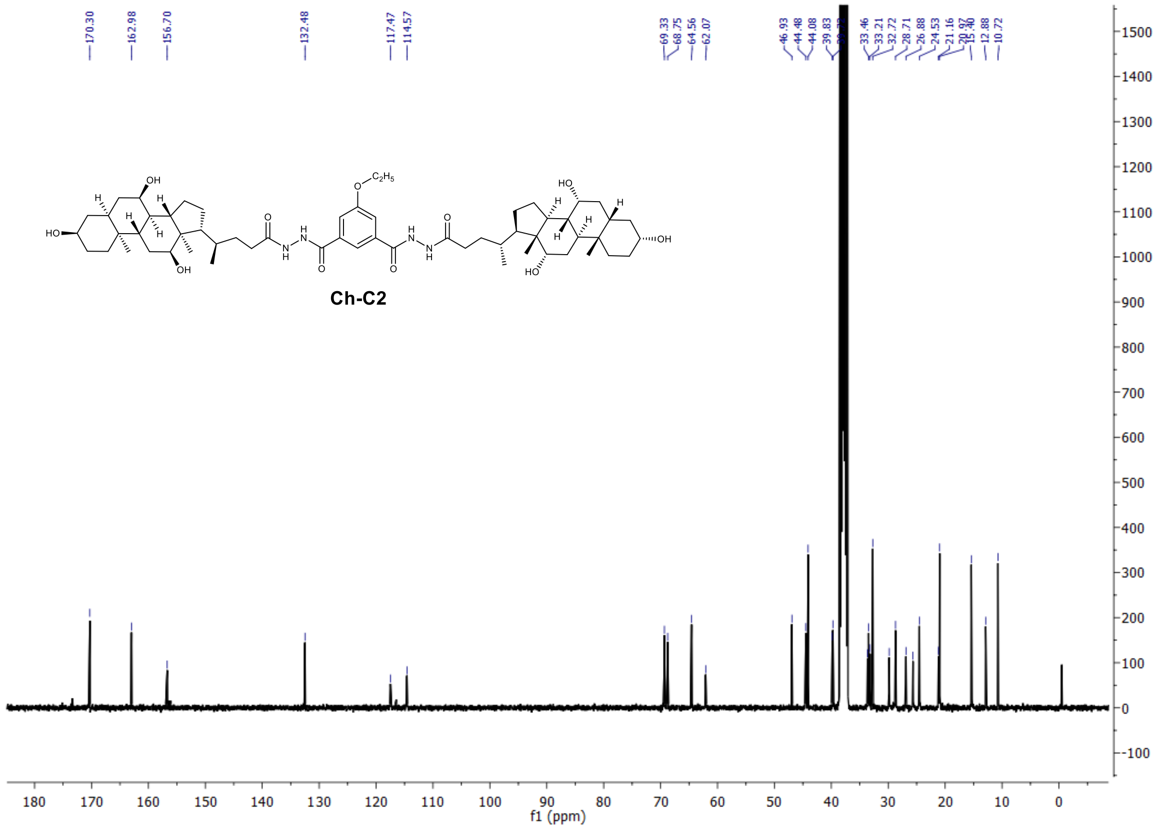
170.30
162.98
156.70
132.48
117.47
114.57
69.33
68.75
64.56
62.07
46.93
44.48
44.08
39.83
33.46
33.21
32.72
28.71
26.88
24.53
21.16
20.97
12.88
10.72
Ch-C2
f1 (ppm)

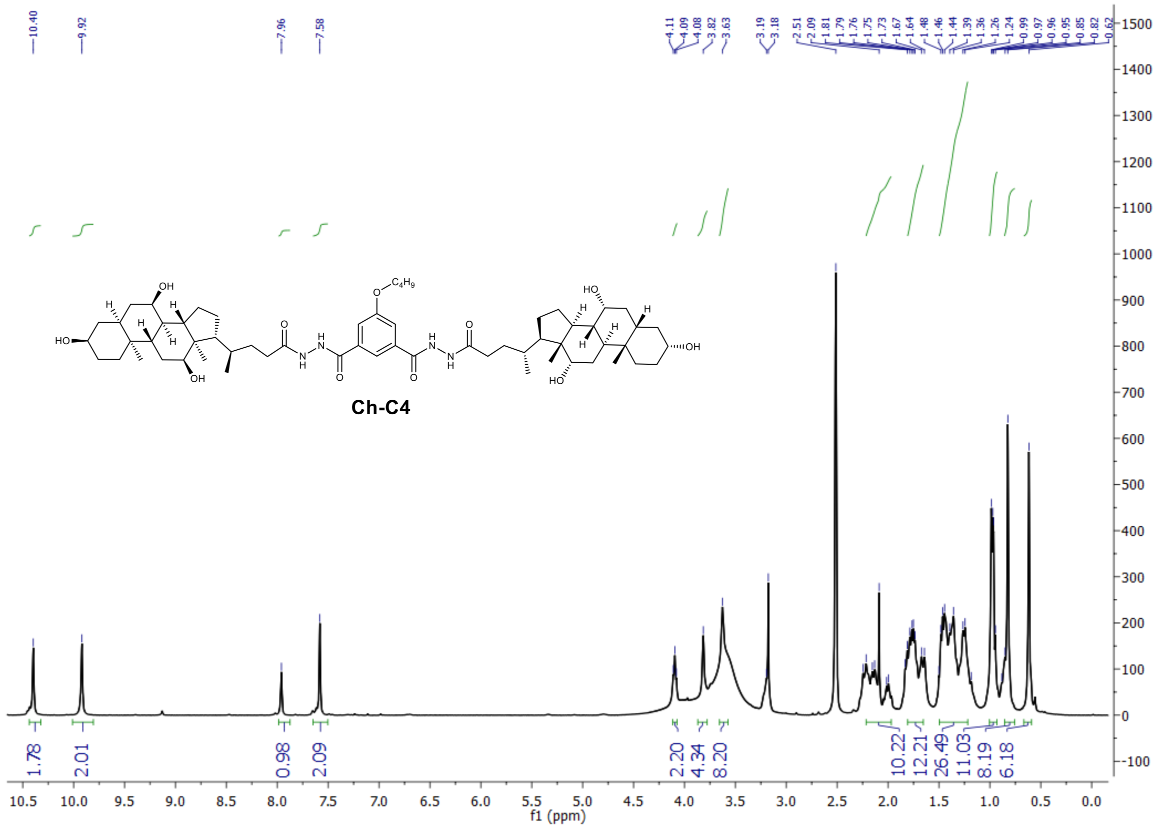

10.40
9.92
7.96
7.58
4.11
4.09
4.08
3.82
3.63
3.19
3.18
2.51
2.09
1.81
1.79
1.76
1.75
1.73
1.67
1.64
1.48
1.46
1.44
1.39
1.36
1.26
1.24
0.99
0.97
0.96
0.95
0.85
0.82
0.62
Ch-C4
1.78
2.01
0.98
2.09
2.20
4.34
8.20
10.22
12.21
26.49
11.03
8.19
6.18
f1 (ppm)

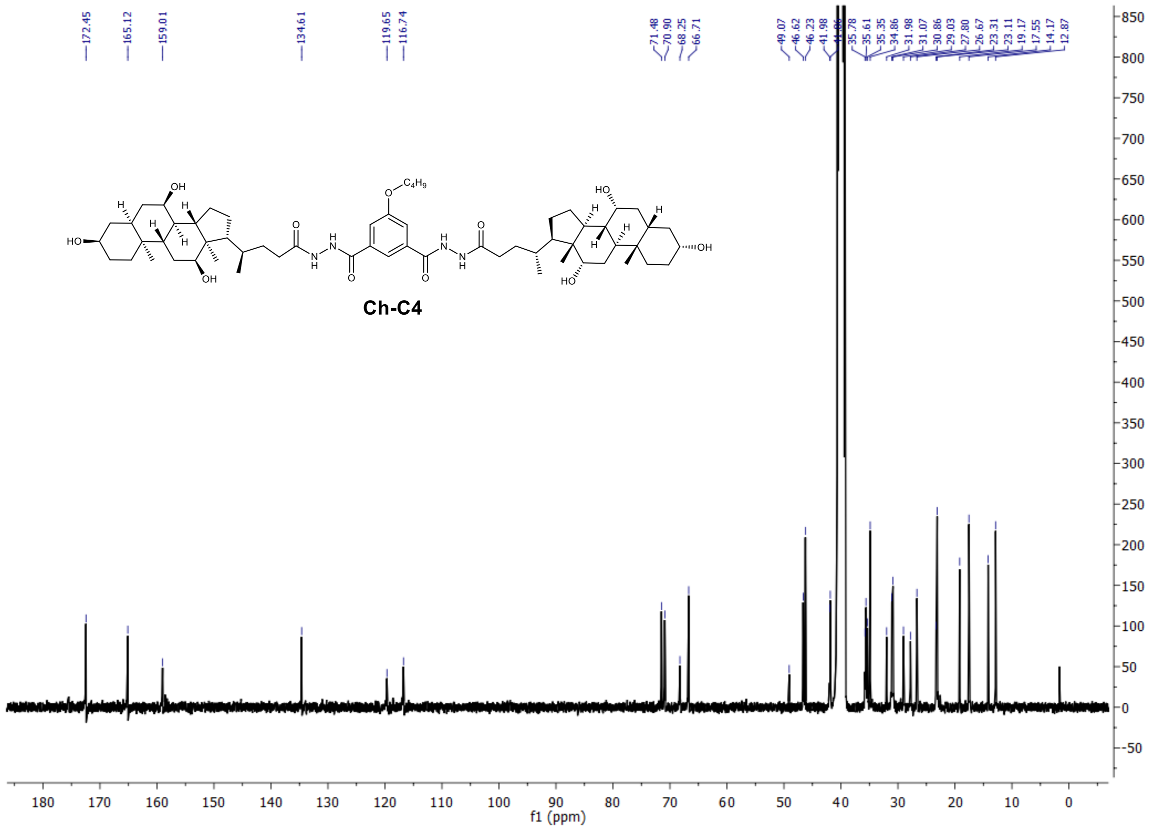

172.45
165.12
159.01
134.61
119.65
116.74
71.48
70.90
68.25
66.71
49.07
46.62
46.23
41.98
35.78
35.61
35.35
34.86
31.98
31.07
30.86
29.03
27.80
26.67
23.31
19.17
17.55
14.17
12.87
Ch-C4
f1 (ppm)

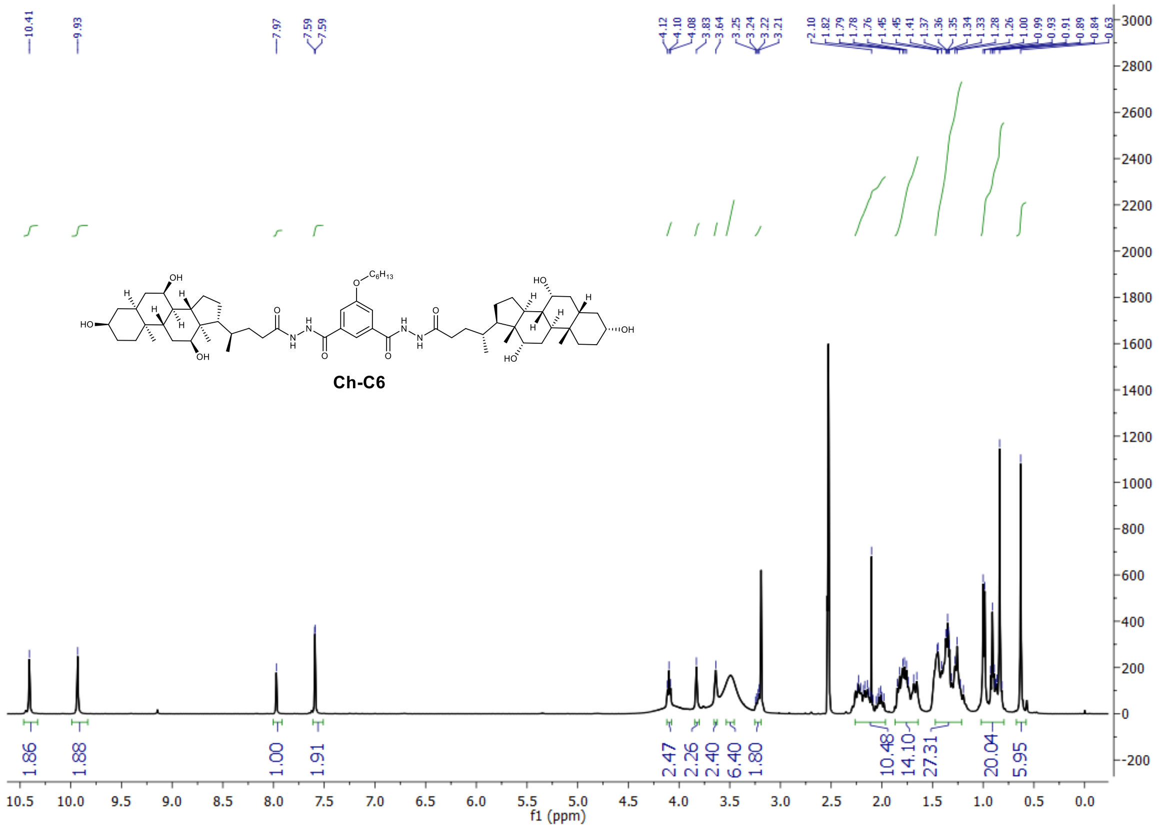

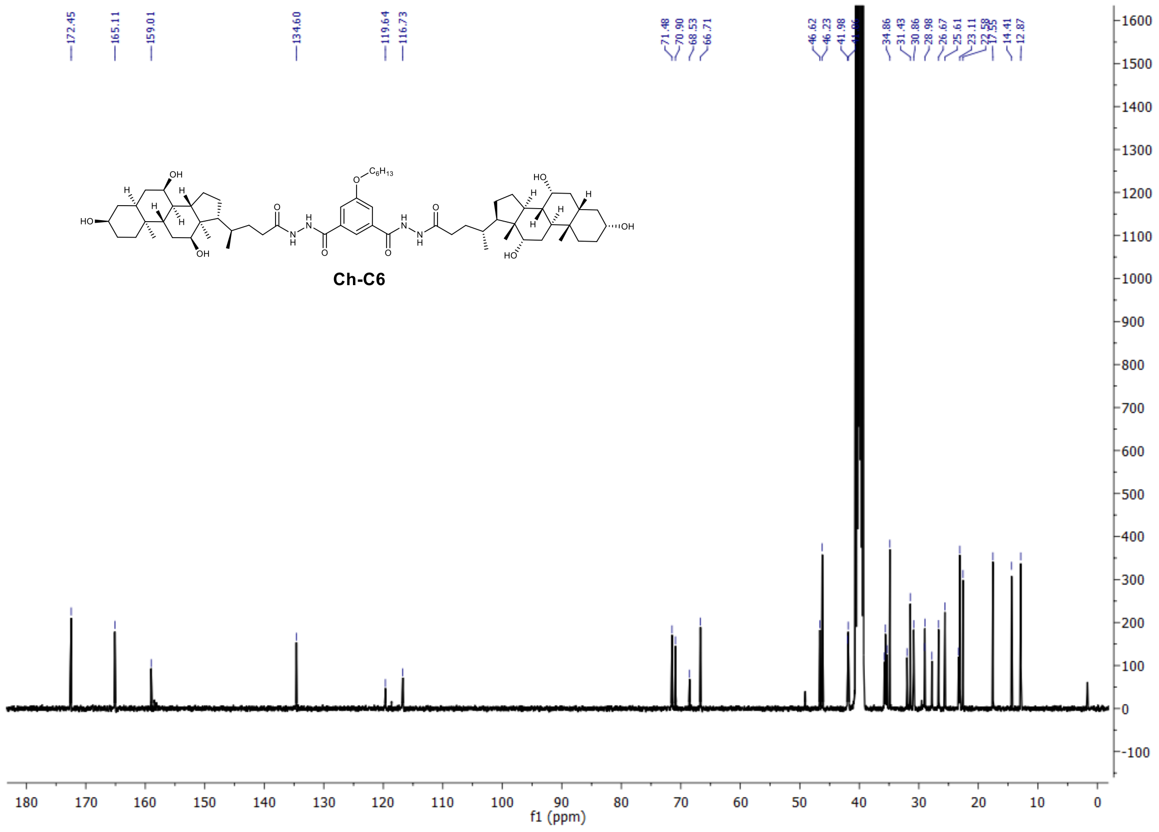

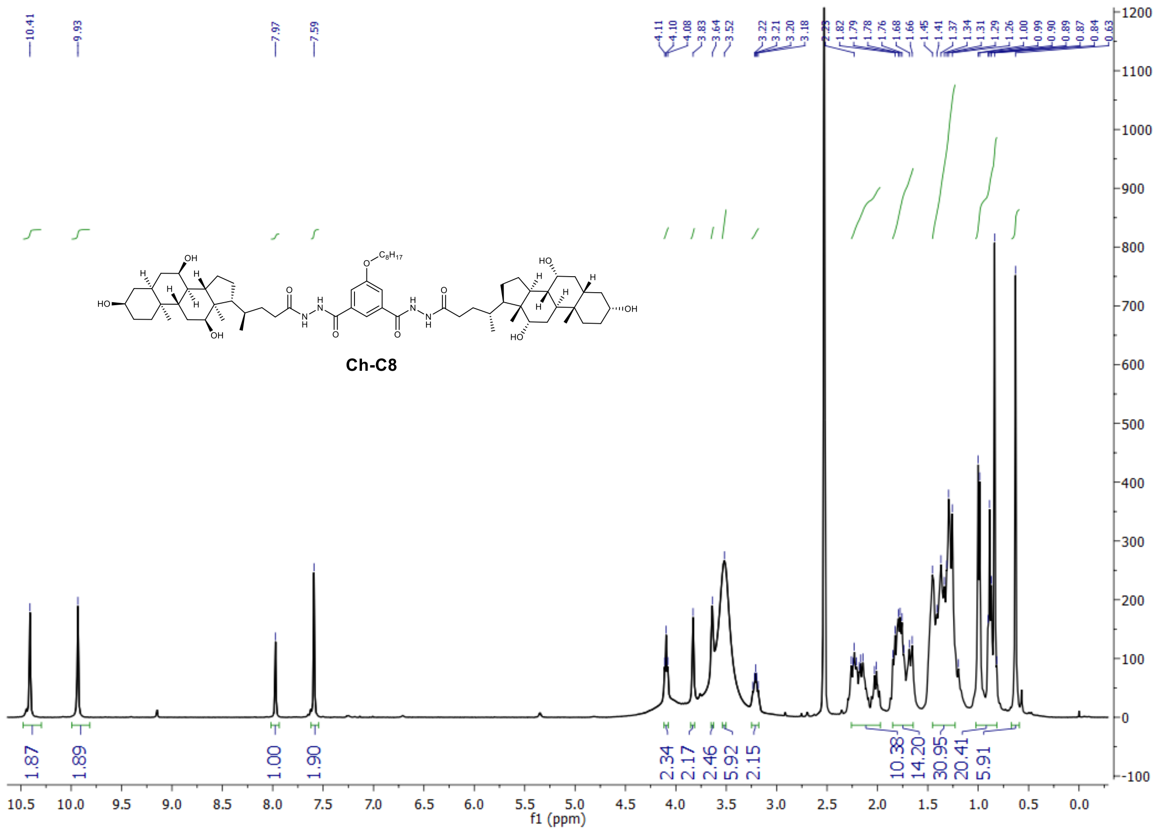

Ch-C8

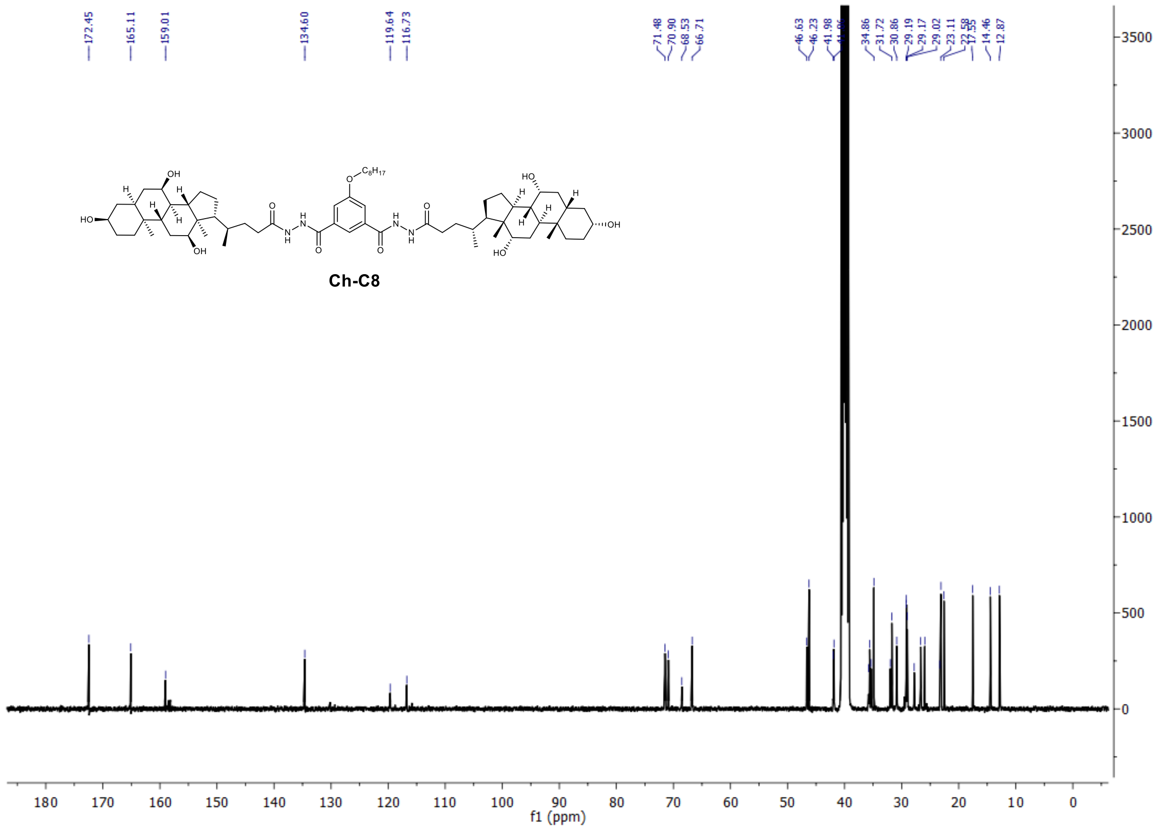

Ch-C8

Supplementary References

1 Wang, J., Wolf, R. M., Caldwell, J. W.; Kollman, P. A. & Case, D. A., Development and testing of a general amber force field. *J. Comput. Chem.* **25**, 1157 (2004).

2 Berendsen, H. J. C., Spoel, D. V. D. & Drunen, R. V., GROMACS: a message passing parallel molecular dynamics implementation. *Comput. Phys. Comm.* **95**, 43 (1995).

3 Hess, B., Bekker, H., Berendsen, H. J. C. & Fraaije, J. G. E. M., LINCS: a linear constraint solver for molecular simulations. *J. Comput. Chem.* **18**, 1463 (1997).

4 Jorgensen, W. L., Chandrasekhar, J., Madura, J. D., Impey, R. W. & Klein, M. L., Comparison of simple potential functions for simulating liquid water. *J. Chem. Phys.* **79**, 926 (1983).

5 Joung, I. S. & Cheatham III, T. E., Determination of alkali and halide monovalent ion parameters for use in explicitly solvated biomolecular simulations. *J. Phys. Chem. B* **112**, 9020 (2008).

6 Essmann, U., Perera, L. & Berkowitz, M. L., A smooth particle mesh Ewald method. *J. Chem. Phys.* **103**, 8577 (1995).

7 Dickson, C. J., Madej, B. D., Skjevik, Å. A., Betz, R. M., Teigen, K., Gould, I. R. & Walker, R. C., Lipid14: The Amber Lipid Force Field. *J. Chem. Theory Comput.* **10**, 865 (2014).
